# Parental legacy, demography, and admixture influenced the evolution of the two subgenomes of the tetraploid *Capsella bursa-pastoris* (Brassicaceae)

**DOI:** 10.1371/journal.pgen.1007949

**Published:** 2019-02-15

**Authors:** Dmytro Kryvokhyzha, Adriana Salcedo, Mimmi C. Eriksson, Tianlin Duan, Nilesh Tawari, Jun Chen, Maria Guerrina, Julia M. Kreiner, Tyler V. Kent, Ulf Lagercrantz, John R. Stinchcombe, Sylvain Glémin, Stephen I. Wright, Martin Lascoux

**Affiliations:** 1 Plant Ecology and Evolution, Department of Ecology and Genetics, Evolutionary Biology Centre and Science for Life Laboratory, Uppsala University, Uppsala, Sweden; 2 Department of Ecology and Evolution, University of Toronto, Toronto, Canada; 3 Department of Biological and Environmental Sciences, University of Gothenburg, Gothenburg, Sweden; 4 Computational and Systems Biology Group, Genome Institute of Singapore, Agency for Science, Technology and Research (A*Star), Singapore; 5 CNRS, Université de Rennes 1, ECOBIO (Ecosystémes, biodiversité, évolution) - UMR 6553, F-35000 Rennes, France; University of Cologne, GERMANY

## Abstract

Allopolyploidy is generally perceived as a major source of evolutionary novelties and as an instantaneous way to create isolation barriers. However, we do not have a clear understanding of how two subgenomes evolve and interact once they have fused in an allopolyploid species nor how isolated they are from their relatives. Here, we address these questions by analyzing genomic and transcriptomic data of allotetraploid *Capsella bursa-pastoris* in three differentiated populations, Asia, Europe, and the Middle East. We phased the two subgenomes, one descended from the outcrossing and highly diverse *Capsella grandiflora* (*Cbp*_*Cg*_) and the other one from the selfing and genetically depauperate *Capsella orientalis* (*Cbp*_*Co*_). For each subgenome, we assessed its relationship with the diploid relatives, temporal changes of effective population size (*N*_*e*_), signatures of positive and negative selection, and gene expression patterns. In all three regions, *N*_*e*_ of the two subgenomes decreased gradually over time and the *Cbp*_*Co*_ subgenome accumulated more deleterious changes than *Cbp*_*Cg*_. There were signs of widespread admixture between *C. bursa-pastoris* and its diploid relatives. The two subgenomes were impacted differentially depending on geographic region suggesting either strong interploidy gene flow or multiple origins of *C. bursa-pastoris*. Selective sweeps were more common on the *Cbp*_*Cg*_ subgenome in Europe and the Middle East, and on the *Cbp*_*Co*_ subgenome in Asia. In contrast, differences in expression were limited with the *Cbp*_*Cg*_ subgenome slightly more expressed than *Cbp*_*Co*_ in Europe and the Middle-East. In summary, after more than 100,000 generations of co-existence, the two subgenomes of *C. bursa-pastoris* still retained a strong signature of parental legacy but their evolutionary trajectory strongly varied across geographic regions.

## Author summary

Allopolyploid species have two or more sets of chromosomes that originate from hybridization of different species. It remains largely unknown how the two genomes evolve in the same organism and how strongly their evolutionary trajectory depends on the initial differences between the two parental species and the specific demographic history of the newly formed allopolyploid species. To address these questions, we analyzed the genomic and gene expression variation of the shepherd’s purse, a recent allopolyploid species, in three regions of its natural range. After ∼100,000 generations of co-existence within the same species, the two subgenomes had still retained part of the initial difference between the two parental species in the number of deleterious mutations reflecting a history of mating system differences. This difference, as well as differences in patterns of positive selection and levels of gene expression, also strongly depended on the specific histories of the three regions considered. Most strikingly, and unexpectedly, the allopolyploid species showed signs of hybridization with different diploid relatives or multiple origins in different parts of its range. Regardless if it was hybridization or multiple origins, this profoundly altered the relationship between the two subgenomes in different regions. Hence, our study illustrates how both the genomic structure and ecological arena interact to determine the evolutionary trajectories of allopolyploid species.

## Introduction

Allopolyploidy, the origin of polyploids from two different ancestral lineages, poses serious evolutionary challenges since the presence of two divergent sub-genomes may lead to perturbation of meiosis, conflicts in gene expression regulation, protein-protein interactions, and transposable element suppression [1–3]. Whole genome duplication also masks new recessive mutations thereby decreasing selection efficacy [4, 5]. This relaxation of selection, together with the strong speciation bottleneck and shift to self-fertilization that often accompany polyploidy [6], ultimately increases the frequency of deleterious mutations retained in the genome [7, 8]. All of these consequences of allopolyploidy can have a negative impact on fitness and over evolutionary time may contribute to the patterns of duplicate gene loss, a process referred to as diploidization [5, 9, 10]. Yet, allopolyploid lineages often not only establish and persist but may even thrive and become more successful than their diploid progenitors and competitors, with larger ranges and higher competitive ability [11–20]. The success of allopolyploids is usually explained by their greater evolutionary potential. Having inherited two genomes that evolved separately, and sometimes under drastically different conditions, allopolyploids should have an increased genetic toolbox, assuming that the two genomes do not experience severe conflicts [21–23]. This greater evolutionary potential of allopolyploids can be further enhanced by genomic rearrangements, alteration of gene expression and epigenetic changes [4, 5, 24–30].

All of these specific features come into play during the demographic history of allopolyploids. Demographic processes occurring when a species extends its range, such as successive bottlenecks or periods of rapid population growth in the absence of competition, are expected to have a profound impact on evolutionary processes, especially in populations at the front of the expansion range. Species that went through repeated bottlenecks during their range expansion are expected to have reduced genetic variation and higher genetic load than more ancient central populations [31, 32]. Similarly, range expansions can also lead to contact and admixture with related species. Such admixture can in turn shift the evolutionary path of the focal species. Finally, range expansion will expose newly formed allopolyploid populations to divergent selective pressures, providing the possibility of differentially exploiting duplicated genes, and creating asymmetrical patterns of adaptive evolution in different parts of the range.

**Fig 1:**
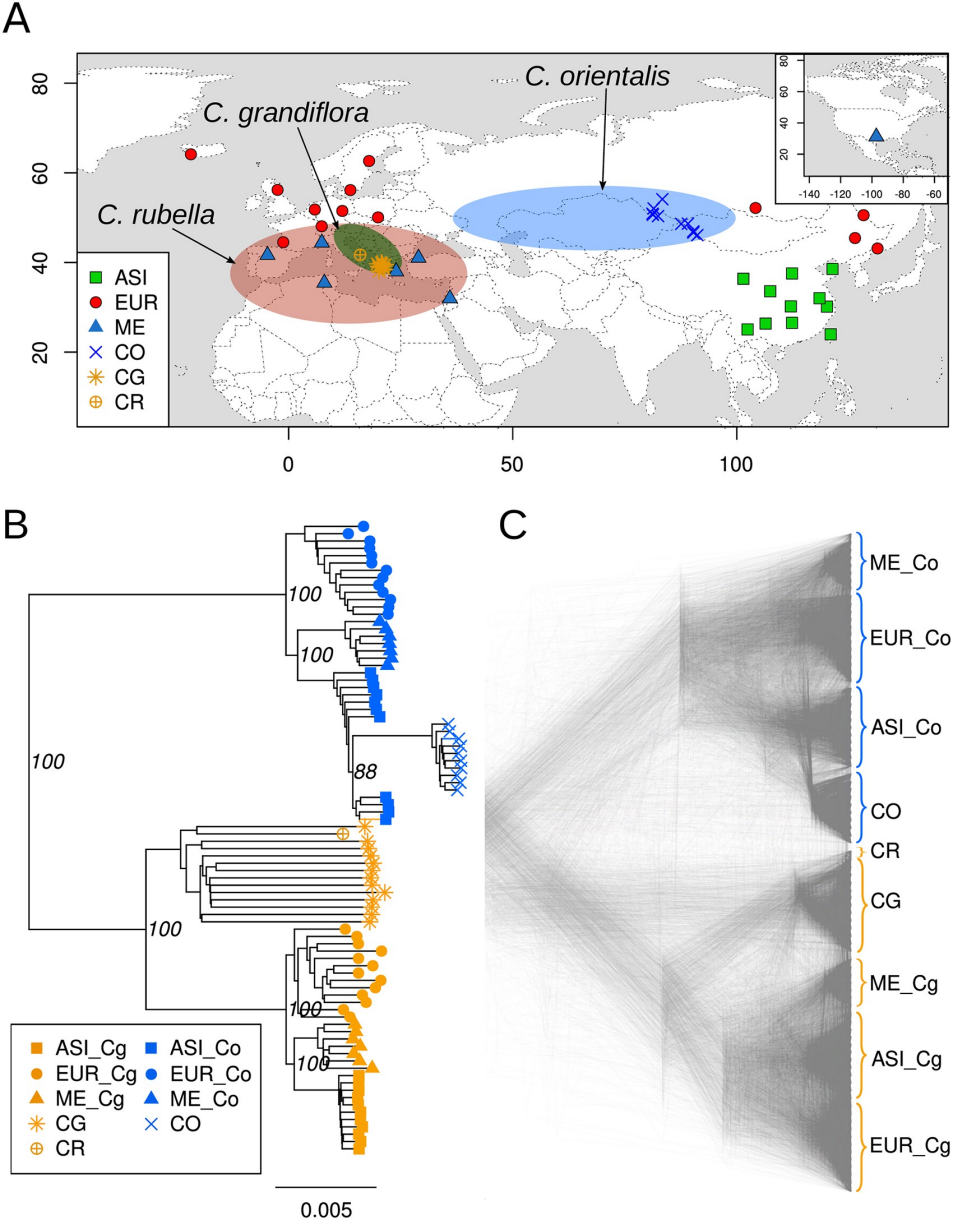
Distribution ranges, sampling locations and phylogenetic relationships of *Capsella* species used in this study. (A) Approximative distribution ranges of *C. orientalis*, *C. grandiflora*, and *C. rubella* and sampling locations of *C. bursa-pastoris*. *C. bursa-pastoris* has a worldwide distribution, so its distribution range is not specifically depicted. ASI, EUR ME, CO, CG, CR indicate Asian, European and Middle Eastern populations of *C. bursa-pastoris*, *C. orientalis*, *C. grandiflora*, and *C. rubella*, respectively. The distribution ranges are defined according to Hurka et al. [38]. (B) Whole genome NJ tree showing the absolute divergence between different populations of *C. bursa-pastoris* at the level of subgenomes. The *Cbp*_*Co*_ and *Cbp*_*Cg*_ subgenomes are marked with Co and Cg. The bootstrap support based on 100 replicates is shown only for the major clades. The root *N. paniculata* is not shown. (C) Density tree visualizing of 1002 NJ trees reconstructed with 100 Kb sliding windows.

In this paper, we aim to characterize the evolution of the genome of a recent allopolyploid species during its range expansion. In particular, we explore whether the two subgenomes have similar or different evolutionary trajectories in hybridization, selection and gene expression. The widespread allopolyploid *C. bursa-pastoris* is a promising system for studying the evolution of polyploidy, with available information on its two progenitor diploid species and their current distribution. *C. bursa-pastoris*, a selfing species, originated from the hybridization of the *Capsella orientalis* and *Capsella grandiflora / rubella* lineages some 100-300 kya [10]. *C. orientalis* is a genetically depauperate selfer occurring across the steppes of Central Asia and Eastern Europe. In contrast, *C. grandiflora* is an extremely genetically diverse obligate outcrosser which is primarily confined to a small distribution range in the mountains of Northern Greece and Albania. The fourth relative, *C. rubella*, a selfer recently derived from *C. grandiflora*, occurs around the Mediterranean Sea. There is evidence for unidirectional gene flow from *C. rubella* to *C. bursa-pastoris* [33]. Among all *Capsella* species, only *C. bursa-pastoris* has a worldwide distribution (Fig 1A), which may be partially due to extremely recent colonization and associated with human population movements [34]. A recent study reveals that in Eurasia, *C. bursa-pastoris* is divided into three genetic clusters—Middle East, Europe, and Asia—with low gene flow among them and strong differentiation both at the nucleotide and gene expression levels [34, 35]. Reconstruction of the colonization history using unphased genomic data suggested that *C. bursa-pastoris* spread from the Middle East towards Europe and then into Eastern Asia. This colonization history resulted in a typical reduction of nucleotide diversity with the lowest diversity being in the most distant Asian population [34].

Whether adaptation on the two distinct non-recombining [36, 37] subgenomes of *C. bursa-pastoris* contributed to its rapid population expansion and how they were in return affected by it, remains unclear. Previous studies either ignored the population history of *C. bursa-pastoris* or failed to consider the two subgenomes separately. In a recent study that does not consider the population demographic history within *C. bursa-pastoris*, Douglas et al. [10] concluded that there is no strong sign of diploidization in *C. bursa-pastoris* and most of its variation is the result of the legacy from the parental lineages with some relaxation of purifying selection caused by both the transition to self-fertilization and the greater masking of deleterious mutations. Kryvokhyzha et al. [35] considered population history but did not separate the two subgenomes, and showed that variation in gene expression among Asian, European and Middle Eastern accessions strongly reflects the population history with most of the differences among populations explained by genetic drift. We extend these previous studies by analyzing the genome-wide expression and polymorphism patterns of the two subgenomes of *C. bursa-pastoris* in 31 accessions sampled across its natural range in Eurasia. We demonstrate that the two subgenomes follow distinct evolutionary trajectories in different populations and that these trajectories are influenced by both range expansion and hybridization with diploid relatives. Our study illustrates the need to account for demographic and ecological differences among populations when studying the evolution of subgenomes of allopolyploid species.

## Results

### Phasing subgenomes

The disomic inheritance of *C. bursa-pastoris* [36, 37] allowed us to successfully phase most of the heterozygous sites in the 31 samples analyzed in this study (Fig 1A, S1 Table). Out of 7.1 million high confidence SNPs, our phasing procedure produced an alignment of 5.4 million phased polymorphic sites across the 31 accessions of *C. bursa-pastoris*. Scaling these phased SNPs to the whole genome resulted in the alignment of 80.6 Mb that had the same level of heterozygosity as the unphased data. The alignment of these whole genome sequences of *C. bursa-pastoris* with 13 sequences of *C. grandiflora*, 10 sequences of *C. orientalis*, one sequence of *C. rubella* (the reference), and one sequence of *N. paniculata* used here as an outgroup, yielded 12.8 million polymorphic sites that we used in all analyses. The information for each accession is provided in S1 Table.

We analyzed the structure of the phased data with phylogenetic analyses. The separation of the two subgenomes was strongly supported in the reconstructed whole genome tree (Fig 1B). The grouping with a corresponding parental species was also maintained in the phylogenetic analyses of each subgenome separately (S1 Fig) as expected given assumptions of the phasing approach. The tree consisted of two highly supported (100% bootstrap) major clades grouping *C. grandiflora* and the *C. grandiflora / rubella* lineage descended subgenome of *C. bursa-pastoris* (hereafter the *Cbp*_*Cg*_ subgenome), on the one hand, and *C. orientalis* and the *C. orientalis* lineage descended subgenome of *C. bursa-pastoris* (hereafter the *Cbp*_*Co*_ subgenome), on the other hand. We also analyzed phylogenetic signals at a finer genomic scale using a sliding window approach with 100-kb window size (Fig 1C). Exclusive monophyly of *C. orientalis* with the *Cbp*_*Co*_ subgenome, and *C. grandiflora* and *C. rubella* with *Cbp*_*Cg*_ subgenome was detected in 95% and 83% of trees, respectively (S2 Fig).

Comparing linkage disequilibrium (LD) decay within and across subgenomes—e.g. comparing *r*^2^ between SNP1 and SNP2 in *Cbp*_*Cg*_ and *r*^2^ between SNP1 in *Cbp*_*Cg*_ and SNP2 in *Cbp*_*Co*_—suggested largely consistent phasing within each subgenome after accounting for population history (S3 Fig). Within homeologues (particularly *Cbp*_*Cg*_) we observed high LD in the Middle Eastern and European samples with gradual decay as genetic distance increases. LD decayed more rapidly in the Asian samples than in the other populations (possibly due to less population structure and expansion) but also showed the expected decay within both subgenomes. In contrast, *r*^2^ with SNPs from the other subgenome or randomly selected from either subgenome was substantially lower and did not show any decay within 10 KB, particularly in the Middle Eastern and European samples. High LD and gradual decay within, and not between, subgenomes suggested phasing was largely effective in separating the two subgenomes and preserving signals of their demographic history.

The genomic data analyzed in this study were phased using the computational phasing of reads mapped to the *C. rubella* reference (HapCUT method) and was validated with mapping the same reads to the recent assembly of *C. bursa-pastoris* [37] (HomeoRoq method, see Material and methods). The final alignment between the alternatively phased dataset comprised 800 K SNPs. This alignment was relatively small because mapping to two subgenomes resulted in 2x smaller coverage and subsequent genotype calling, filtering, phasing, and alignment cumulatively further reduced this size (S4 Fig). Phylogenetic trees reconstructed for 10 K sliding windows showed 100% split between *Cbp*_*Cg*_ and *Cbp*_*Co*_ subgenomes (S5 Fig). Using a smaller window of 1 K resulted in 83% of trees with mutual monophyly of *Cbp*_*Cg*_ and *Cbp*_*Co*_ subgenomes (S6 Fig). The other 17% still showed a split between *Cbp*_*Cg*_ and *Cbp*_*Co*_ for the majority of samples, but some single samples were not consistent with the mutual monophyly of the two subgenomes. This could be either due to incomplete lineage sorting or potential local phasing errors. Nevertheless, the major clustering into two subgenomes and three populations was largely maintained in the 17% of trees (S7 Fig). In all trees, the same samples phased with two alternative methods (HapCUT and HomeoRoq) clustered together for most of the European samples. The Middle Eastern cluster showed slightly less consistent clustering, and the Asian samples showed clustering into two groups according to the phasing method. The cause of these discrepancies is difficult to define because errors are possible in both datasets. To localize the problematic regions, we also compared the phased dataset analyzed in this study (HapCUT phased) with the assembly sequences of *C. bursa-pastoris* [37]. This comparison covered almost all positions of the dataset analyzed in this study (10.9Mb out of 12.7Mb). Most of the discrepancies were located in the vicinity of pericentromeric regions (S8 Fig)), where assembling reads is problematic. In summary, there was a high concordance of the results obtained with the different phasing methods and those that differed are unlikely to have affected the main results of the study.

### A geographically structured differentiation between subgenomes

**Fig 2:**
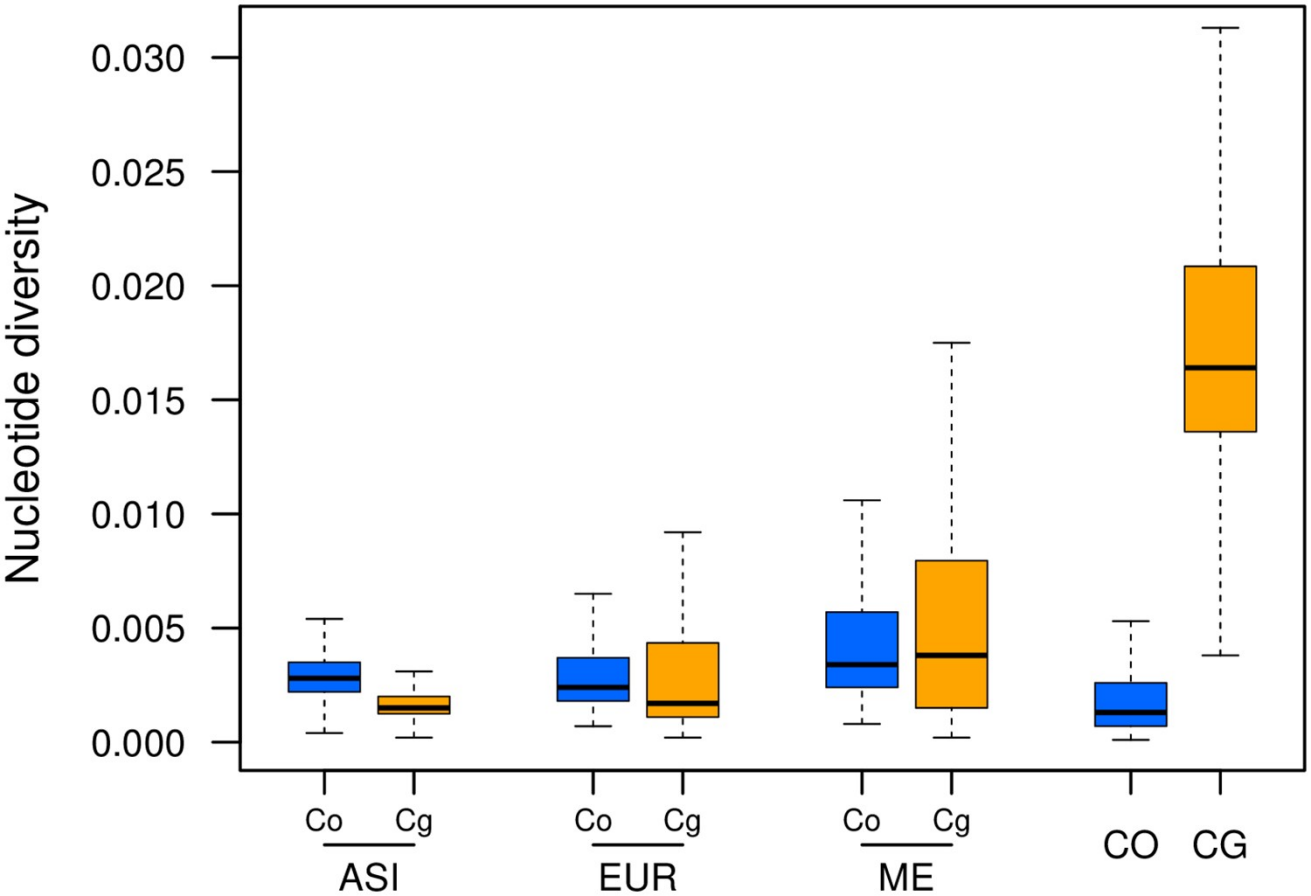
Variation in nucleotide diversity (*π*) between populations of *C. bursa-pastoris* and parental species. This boxplot shows the distributions of *π* estimated along the genome using 100 Kb sliding windows. Co and Cg indicate *C. orientalis* and *C. grandiflora / rubella* descendant subgenomes, respectively. ASI, EUR ME, CO and CG correspond to Asian, European and Middle Eastern populations of *C. bursa-pastoris*, *C. orientalis*, and *C. grandiflora*, respectively.

For both subgenomes, the three *C. bursa-pastoris* populations, Asia (ASI), Europe (EUR) and Middle East (ME), constituted well-defined phylogenetic clusters (Fig 1B and 1C). However, the relationships of each subgenome with its parental species differed. The *Cbp*_*Cg*_ subgenome formed a monophyletic clade with *C. grandiflora* at its base. In contrast, the *Cbp*_*Co*_ subgenome was paraphyletic with *C. orientalis* that clustered within the ASI group instead of being outside of all *C. bursa-pastoris* *Cbp*_*Co*_ subgenomes (this was observed in both phasing methods S9 Fig). This clustering was unexpected and suggested potential gene flow between the ASI group and *C. orientalis* or multiple origins of the *Cbp*_*Co*_ subgenome. Nucleotide diversity was higher on the *Cbp*_*Cg*_ subgenome than on the *Cbp*_*Co*_ subgenome for both EUR and ME (Fig 2, S10 Fig, S2 Table), though the difference was significant only for EUR (p-values: 0.005 and 0.154 for EUR and ME, respectively). The opposite pattern was observed for ASI (Fig 2): there the nucleotide diversity in the *Cbp*_*Co*_ subgenome was significantly higher than in the *Cbp*_*Cg*_ subgenome (p-value < 0.0001). Interestingly, the diversity of the *Cbp*_*Co*_ subgenome in all populations was significantly higher than the diversity of its parental species, *C. orientalis* (p-value < 0.0001).

### A 10-fold or greater decrease in *N*_*e*_ in all selfers but not in *C. grandiflora*

**Fig 3:**
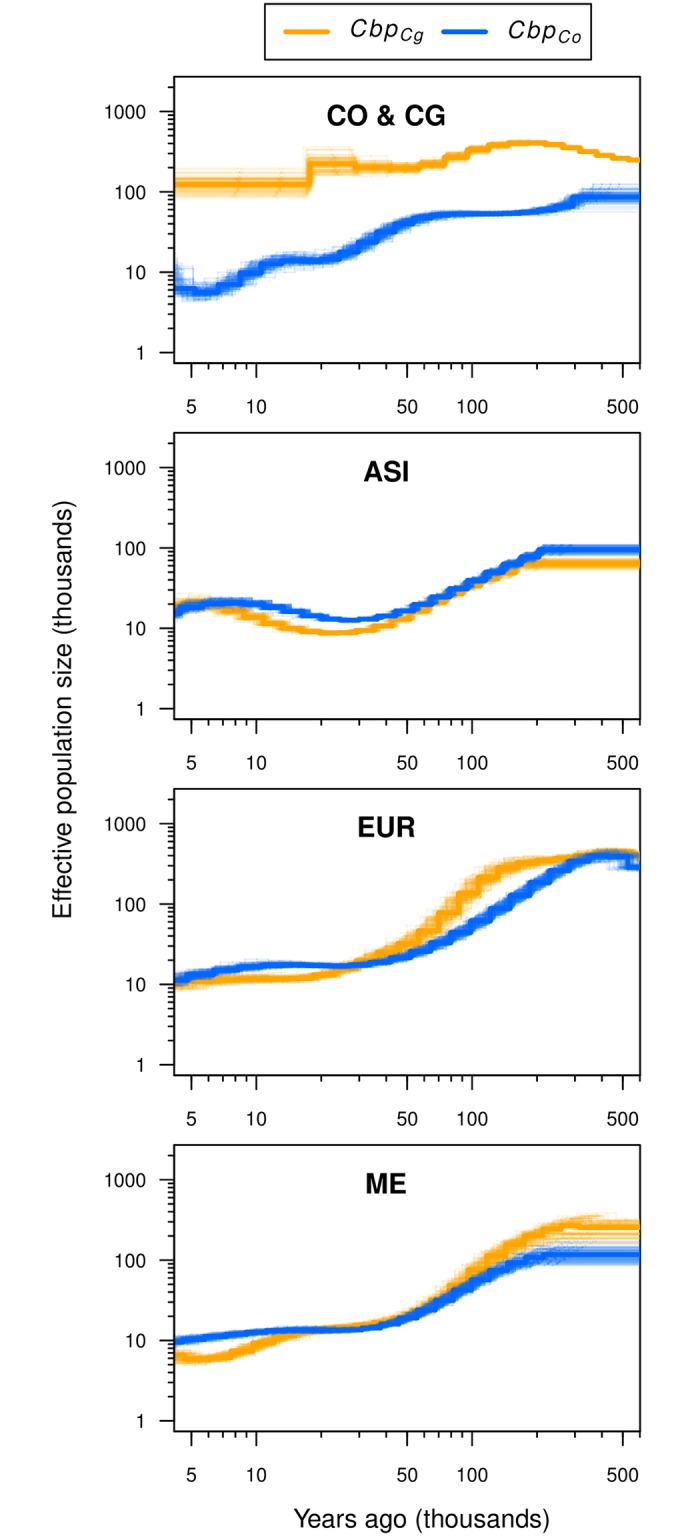
Population size histories of *C. bursa-pastoris* and its parental species. Effective population sizes were inferred with *PSMC* using whole-genome sequences from a pair of haplotypes per population (thick lines) and 100 bootstrap replicates (thin lines). The estimates for different pairs were similar and shown in S11 Fig. Co and Cg specify the *Cbp*_*Co*_ and *Cbp*_*Cg*_ subgenomes of *C. bursa-pastoris* and corresponding parental species in the CO & CG plot. ASI, EUR, ME, CO & CG indicate Asian, European and Middle Eastern populations of *C. bursa-pastoris*, and *C. orientalis* and *C. grandiflora*, respectively. The axes are in log scale and the most recent times where *PSMC* is less reliable were excluded.

To reconstruct the changes in effective population size (*N*_*e*_) over time in the three *C. bursa-pastoris* populations and the two ancestral species, we used a pairwise sequentially Markovian coalescent model (*PSMC*). First, we reconstructed the demographic histories of *C. orientalis* and *C. grandiflora* (Fig 3). In *C. grandiflora*, *N*_*e*_ was mostly constant with some slight decrease in the recent past, but the *N*_*e*_ of *C. orientalis* decreased continuously towards the present. In *C. bursa-pastoris*, despite a simultaneous rapid range expansion, *N*_*e*_ of EUR and ME populations also gradually decreased starting from around 100-200 kya. The ASI population showed a similar pattern but with population size recovery around 5-10 kya and a subsequent decrease to the same *N*_*e*_ as in EUR and ME. The *N*_*e*_ patterns of the two subgenomes were similar within each population. Overall, the *N*_*e*_ history of *C. bursa-pastoris* was most similar to that of its selfing ancestor, *C. orientalis*. We also verified these *PSMC* results with *SMC++*, which can consider more than two haploid genomes and incorporates linkage disequilibrium (LD) in coalescent hidden Markov models [39]. The general trend was globally the same but the recent decline of *C. orientalis* was sharper and fluctuations in *N*_*e*_ were more pronounced (S11 Fig). In summary, the overall pattern of *N*_*e*_ change over time was mostly the same between the two subgenomes and between the three populations of *C. bursa-pastoris* and it was largely similar to the pattern observed for the diploid selfer *C. orientalis*.

### Phylogenetic relationships between *C. bursa-pastoris* and its parental species vary among populations

**Fig 4:**
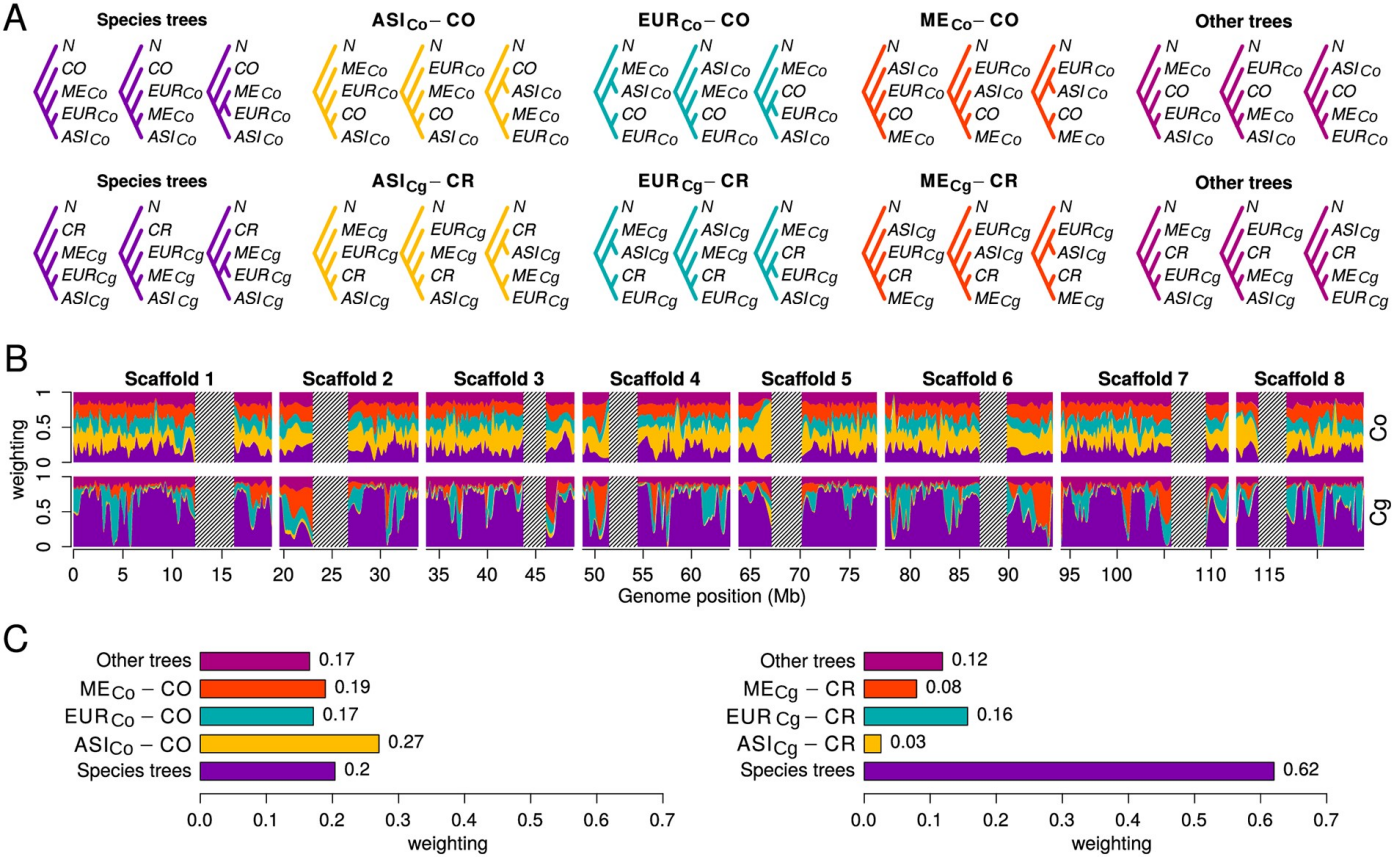
Topology weighting of the three populations of *C. bursa-pastoris* and parental species. (A) Fifteen possible rooted topologies for the three groups of *C. bursa-pastoris* in one subgenome and the corresponding parental species. The topologies are grouped into five main groups. The *Cbp*_*Co*_ and *Cbp*_*Cg*_ subgenomes are marked with Co and Cg. ASI, EUR ME, CO, CR, N indicate Asian, European and Middle Eastern populations of *C. bursa-pastoris*, *C. orientalis*, *C. rubella*, and *N. paniculata*, respectively. (B) Topology weightings for 100 SNP windows plotted along 8 main scaffolds with loess smoothing (span = 1Mb). The tentative centromeric regions are shaded and only eight major scaffolds (chromosomes) are shown. (C) Average weighting for the five main topology groups. The topology groups are in the same order (left-right and bottom-up) and colors in all three plots.

To quantify the relationships between populations of *C. bursa-pastoris* and the two parental species, we applied a topology weighting method that calculates the contribution of each individual group topology to a full tree [40]. We looked at the topologies joining each subgenome of *C. bursa-pastoris* and a corresponding parental lineage. There are 15 possible topologies for three populations of *C. bursa-pastoris*, a parental species, and the root. We grouped these topologies into five main groups: species trees—topologies that place a parental lineage as a basal branch to *C. bursa-pastoris*; three groups that join one of the populations of *C. bursa-pastoris* with a parental lineage and potentially signifies admixture; and all other trees that place a parental lineage within *C. bursa-pastoris* but do not relate it with a particular population of *C. bursa-pastoris* (Fig 4A).

These topology weightings varied along the subgenomes and illustrated distinct patterns between the two subgenomes (Fig 4B). In the *Cbp*_*Co*_ subgenome, the largest average weighting was for the topology grouping the ASI population of *C. bursa-pastoris* with *C. orientalis* (Fig 4C), and the species topology had the second largest average weighting. The difference between the average weighting in these two topology groups was statistically significant (S3 Table). In contrast, the species topologies weighting dominated in the *Cbp*_*Cg*_ subgenome, regardless if *C. rubella* or *C. grandiflora* were used as a parental lineage (Fig 4C, S12 Fig, S4 and S5 Tables). The topology uniting the *Cbp*_*Cg*_ subgenome of the EUR population with *C. rubella* had the largest topology weighting among the topologies indicating admixture between these clusters (Fig 4C). Thus, the two subgenomes differed substantially in the pattern of topology weighting and there were signs of a potential admixture of EUR and ASI with *C. rubella* and *C. orientalis*, respectively.

### *C. bursa-pastoris* subgenomes show signs of admixture with diploid relatives

The phylogenetic grouping of *C. orientalis* with the Asian *Cbp*_*Co*_ subgenome, together with topology weighting results and the relatively elevated nucleotide diversity in this subgenome, suggested the possibility of gene flow between *C. orientalis* and *C. bursa-pastoris* in the ASI population. To test this hypothesis, and at the same time to check for possibilities of gene exchange between *C. bursa-pastoris* and other *Capsella* species, we conducted two complementary tests of admixture.

We first used the ABBA-BABA test, a coalescent-based method that relies on the assumption that alleles under incomplete lineage sorting are expected to be equally frequent in two descendant populations in the absence of admixture between any of them and a third population that diverged earlier on from the same common ancestor [41, 42]. The deviation from equal frequency is measured with the *D*-statistic, which ranges between 0 and 1, with 0 indicating no gene flow and 1 meaning complete admixture. The ABBA-BABA test also provides an estimate of the fraction of the genome that is admixed by comparing the observed difference in ABBA-BABA with the difference expected under a scenario of complete admixture (*f*-statistic). We estimated *D* and *f* for triplets including one diploid species and two populations of *C. bursa-pastoris* represented by the most related subgenome to that species (Table 1). *N. paniculata* was the outgroup in all tests. The *D*-statistic were significantly different from 0 in most of the tests, so we considered all three combinations per test group (see Table 1) to determine the pairs that were the most likely to be admixed. The largest fraction of admixture was identified for the pair of the ASI *Cbp*_*Co*_ subgenome and *C. orientalis* with an estimate of *f* indicating that at least 14% of the ASI *Cbp*_*Co*_ subgenome is admixed. The second largest proportion of admixture was detected between *C. rubella* and the EUR *Cbp*_*Cg*_ subgenome with *f* estimate of at least 8%. The estimates for tests with *C. grandiflora* were the smallest but similar to those obtained for *C. rubella*. The latter may reflect the strong genetic similarity between these two species rather than real gene flow between *C. grandiflora* and *C. bursa-pastoris* which, based on crosses, seems unlikely. Indeed, we crossed individuals from the three populations of *C. bursa-pastoris* with their three diploid relatives to test for the presence of reproductive barriers. Regardless of the direction of the crosses, all crosses between *C. rubella* and the three populations of *C. bursa-pastoris* produced viable F1 seeds. In contrast, crosses involving *C. grandiflora* mostly failed and the abortion rate approached 100%. Details on these crosses are provided in S1 Appendix. The admixture with *C. rubella* in EUR and *C. orientalis* in ASI was also supported by the unphased and complete (i.e. no missing genotypes) genomic data (S6 and S7 Tables). Finally, it should be pointed out that given that evidence for *C. bursa-pastoris* monophyly is weak, it is also possible that the signals of admixture with the parental species that we are detecting here actually reflect introgression from an independently-arisen *C. bursa-pastoris* into either *Cbp*_*Co*_ or *Cbp*_*Cg*_ subgenomes.

**Table 1:** Results of the ABBA-BABA tests assessing admixture between *C. bursa-pastoris* and *C. orientalis*, *C. grandiflora* and *C. rubella*.

P_1_	P_2_	P_3_	*D* ± error	*Z*-score	*P*-value	*f* ± error (%)
EUR_Co	ASI_Co	CO	0.29 ± 0.03	8.62	<0.0001	22.9 ± 2.5
ME_Co	ASI_Co	CO	0.18 ± 0.04	4.80	<0.0001	14.0 ± 2.8
EUR_Co	ME_Co	CO	0.17 ± 0.03	5.70	<0.0001	11.7 ± 2.4
ASI_Cg	EUR_Cg	CG	0.19 ± 0.01	15.45	<0.0001	19.8 ± 2.2
ASI_Cg	ME_Cg	CG	0.17 ± 0.02	10.14	<0.0001	12.6 ± 2.0
ME_Cg	EUR_Cg	CG	0.06 ± 0.01	5.14	<0.0001	6.1 ± 1.2
ASI_Cg	EUR_Cg	CR	0.61 ± 0.02	26.74	<0.0001	20.1 ± 2.1
ASI_Cg	ME_Cg	CR	0.49 ± 0.03	14.55	<0.0001	10.6 ± 1.6
ME_Cg	EUR_Cg	CR	0.26 ± 0.05	4.84	<0.0001	7.9 ± 1.7

P_1_, P_2_, and P_3_ refer to the three populations used in the ABBA-BABA tests. A significantly positive *D* indicates admixture between P_2_ and P_3_. *f* provides an estimate of the fraction admixture. *Z*-score and *P*-value were estimated with the block jack-knife method. The error term corresponds to a standard error. ASI, EUR and ME are the three populations of *C. bursa-pastoris* with _Co and _Cg indicating different subgenomes. CO, CG, and CR stand for *C. orientalis*, *C. grandiflora*, *C. rubella*, respectively. Every test group is separated by a horizontal line.

We then used *HAPMIX*, a haplotype-based method, which enables capture of both large and fine-scale admixture, as well as an absolute estimate of the proportion of the genome that was admixed. For the analysis of the *Cbp*_*Cg*_ subgenome of *C. bursa-pastoris*, the highest levels of admixture were found consistently across regions to be with the diploid *C. rubella*. In Europe, genomic regions showed an average 18% of admixture with *C. rubella*, followed by 11% in the Middle East, and just 2% in Asia (S8 Table, S13A Fig). All three populations also showed signs of admixture with *C. grandiflora* but to a reduced extent compared to *C. rubella* (7% in Europe, 6% in the Middle East, 0.2% in Asia). *C. rubella* is highly similar to *C. grandiflora*, and as noted above, we expect difficulties in discerning between the two, suggesting that much of the signal of admixture with *C. grandiflora* could in fact be due to admixture with *C. rubella*. Of the regions putatively admixed with *C. grandiflora*, 78%-96% of sites called as admixed overlapped with those from *C. rubella*, none of which occurred in unique regions for *C. grandiflora*. Because of this, and in combination with the reduced genome-wide probability of admixture with the diploid *C. grandiflora* compared to *C. rubella* (e.g. 0.11 compared to 0.24 in Europe), we argue that the signals of admixture with the diploid *C. grandiflora* were likely an artifact of its similarity with the regions of *C. rubella* admixture. These findings in accord with the ABBA-BABA results imply that the *Cbp*_*Cg*_ subgenome has experienced significant admixture with *C. rubella* in Europe, and to a lesser extent in the Middle East.

For the analysis of the *Cbp*_*Co*_ subgenome, signals of admixture with the diploid *C. orientalis* were present in all three populations. In the ME population, genomic regions showed an average 18-21% admixture depending on the reference populations used (S8 Table, S13B Fig). Using the Middle East population for the analysis of the *Cbp*_*Co*_ subgenomes of EUR and ASI, since it was the least admixed in the *HAPMIX* results, yielded 15% *C. orientalis* admixture in Asia, and 14% in Europe. These findings suggest admixture of the diploid *C. orientalis* with the *Cbp*_*Co*_ subgenome across all three geographic regions. Assuming these levels of admixture accurately reflect reality, we do not have a non-admixed reference population to use for *HAPMIX*, and are thus violating a key assumption of the method. *HAPMIX* inferences for the *Cbp*_*Co*_ subgenome should therefore be taken with caution but we note that the results for ASI and ME are generally congruent with the admixture pattern obtained with ABBA-BABA.

### Selection and gene expression

#### Deleterious mutation accumulation in subgenomes reflects parental legacy

We first estimated the nucleotide diversity at 0-fold (*π*_0_) and 4-fold (*π*_4_) degenerate sites and then the ratio *π*_0_/*π*_4_ as a measure of purifying selection. Low values of *π*_0_/*π*_4_ would indicate more efficient purifying selection [43]. As expected, *π*_0_/*π*_4_ was much lower in *C. grandiflora* than in *C. orientalis*. In *C. bursa-pastoris*, purifying selection was more efficient in the *Cbp*_*Cg*_ subgenome than in the *Cbp*_*Co*_ subgenome in both EUR and ME. However, the opposite was observed in the ASI population. For both subgenomes, the ASI population had the highest value of *π*_0_/*π*_4_ even if compared with *C. orientalis* (S14 Fig).

We then investigated the differences in deleterious mutations among subgenomes and populations by classifying nonsynonymous mutations with the *SIFT4G* algorithm that uses site conservation across species to predict the selective effect of nonsynonymous changes [44]. In order to control for possible biases due to the unequal genetic distance between the different genomes and *C. rubella*, we used both *C. rubella* and *A. thaliana* *SIFT4G* annotation databases. Because we were interested in the number of deleterious mutations that accumulated after speciation of *C. bursa-pastoris*, we polarized the mutations of all three species with the reconstructed ancestral sequences of the common ancestors (see Material and methods).

**Fig 5:**
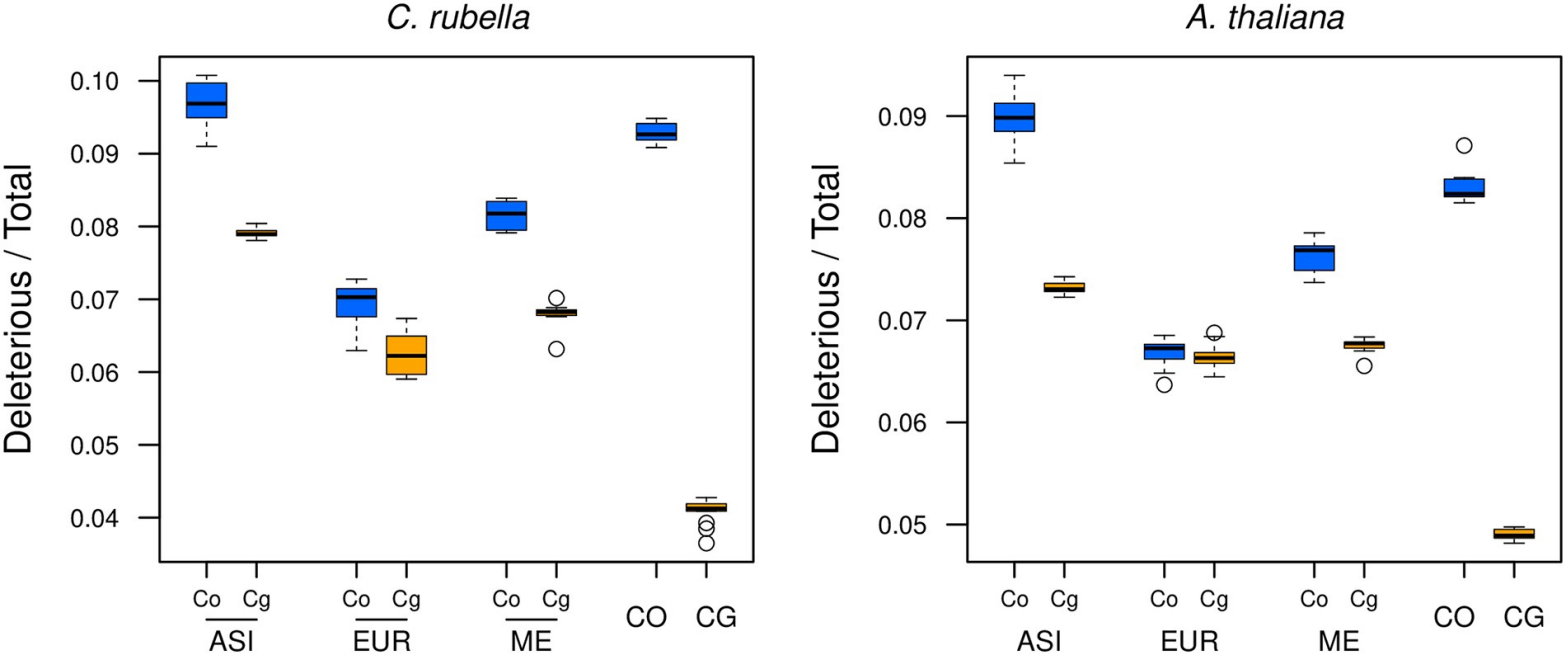
Genetic load in the subgenomes of *C. bursa-pastoris* and its parental species. The proportion of deleterious nonsynonymous changes was estimated with *SIFT4G* on derived alleles, i.e. alleles accumulated after the speciation of *C. bursa-pastoris*. The left plot shows the results obtained with the *C. rubella* database and the right plot those obtained with *A. thaliana* database. Co and Cg are the two subgenomes of *C. bursa-pastoris*. ASI, EUR, ME, CO, CG indicate Asian, European and Middle Eastern populations of *C. bursa-pastoris*, and parental species *C. orientalis* and *C. grandiflora*, respectively.

Regardless of the *SIFT4G* database (*C. rubella* or *A. thaliana*), the proportion of deleterious nonsynonymous sites among derived mutations was always significantly higher in *C. orientalis* and the *Cbp*_*Co*_ subgenomes than in *C. grandiflora* and the *Cbp*_*Cg*_ subgenomes (Fig 5, S9 and S10 Tables). Within *C. bursa-pastoris*, the proportion of deleterious mutations depended on the population considered with the highest value in the ASI population and the smallest in EUR. It is also noteworthy that the proportion of deleterious nonsynonymous sites of the *Cbp*_*Co*_ subgenome in EUR and ME was significantly smaller than that of *C. orientalis* suggesting that a higher effective population size in the *Cbp*_*Co*_ subgenome than in its ancestor led to more efficient purifying selection in these two populations. On the other hand, the proportion of deleterious nonsynonymous sites in the ASI *Cbp*_*Co*_ subgenome was larger than in *C. orientalis*, but this difference was only significant for the *A. thaliana* database. The *Cbp*_*Cg*_ subgenome also had a significantly higher proportion of deleterious sites in ASI than in EUR and ME in all comparisons. In conclusion, the proportion of deleterious sites in the two subgenomes of extant *C. bursa-pastoris* still reflected the differences between the parental species and the efficacy of purifying selection in the different *C. bursa-pastoris* populations was associated to their synonymous nucleotide diversity or, equivalently, to their effective population size.

#### Selective sweeps differ between subgenomes among populations of *C. bursa-pastoris*

**Fig 6:**
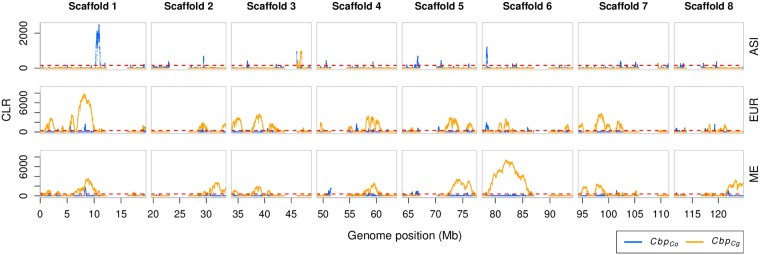
Selective sweep differences between populations of *C. bursa-pastoris*. Selective sweeps are detected with the composite likelihood ratio statistics (CLR) along the *Cbp*_*Co*_ and *Cbp*_*Cg*_ subgenomes in Asian (ASI), European (EUR) and Middle Eastern (ME) populations. The 0.01 significance level defined with data simulated under a standard neutral model (green dashed line). Pericentromeric regions are removed. Only eight major scaffolds (chromosomes) are shown.

The three populations of *C. bursa-pastoris* also differ in patterns of positive selection. Selective sweeps were more significant on the *Cbp*_*Cg*_ subgenome than on the *Cbp*_*Co*_ subgenome in EUR and ME, whereas the opposite was true in the ASI population (Fig 6). The regions harboring significant sweep signals were also larger on the *Cbp*_*Cg*_ subgenome than on the *Cbp*_*Co*_ subgenome in EUR and ME (total length 42 Mb, 50 Mb vs 9 Mb, 3 Mb), whereas in ASI the sweep regions were larger on *Cbp*_*Co*_ than on *Cbp*_*Cg*_ (total length 4 Mb vs 830 Kb). Although the locations of the *Cbp*_*Cg*_ sweep signals in EUR and ME largely overlap, the patterns differed between the two populations. For example, the strongest sweep signal in EUR was located on scaffold 1, whereas the strongest sweep signal in ME was on scaffold 6. In addition, EUR had many sweeps in *Cbp*_*Co*_ subgenome (109 in EUR_Cg, 128 in EUR_Co), but they all were small and hardly above the significance threshold (Fig 6). In the ME population, the sweep signals in the *Cbp*_*Cg*_ subgenome were prevailing both in size and numbers (101 in ME_Cg, 22 in ME_Co). The ASI population differed strongly from both EUR and ME not only because most of its sweep signals were on the *Cbp*_*Co*_ subgenome but also because these sweeps regions were narrower and less pronounced (Fig 6). We also verified these selection signals with the most stringent filtering of phased fragments (phasing state of all SNPs was supported by fixed differences between parental species) and the major differences between population were maintained (S15 Fig). Thus, all three populations of *C. bursa-pastoris* were distinct in their selective sweeps patterns with the ASI population being the one least affected by positive selection.

#### Lack of a strong expression dominance of one subgenome

One can expect differences in selection patterns between subgenomes and populations of *C. bursa-pastoris* to also affect the relative expression of the two subgenomes, or homeologue-specific expression (HSE). In particular, biased adaptation towards one subgenome may select for decreased expression of the other subgenome. Hence, given the observed selection pattern, one would expect the *Cbp*_*Cg*_ subgenome to be overexpressed in EUR and ME, and the *Cbp*_*Co*_ subgenome to be overexpressed in ASI.

To assess HSE, we analyzed the RNA-Seq data of 24 accessions representing all three populations of *C. bursa-pastoris* in a hierarchical Bayesian model that integrates information from both RNA and DNA data [45]. Overall, in agreement with Douglas et al. [10], one subgenome did not dominate the other in the 24 accessions considered together, though a few genes demonstrated a slight expression shift toward the *Cbp*_*Cg*_ subgenome. On average, we assessed HSE in 13,589 genes per accession (range 12,808-15,340) and 18% of them showed significant HSE (posterior probability of HSE > 0.99). The expression ratios between subgenomes (defined here as *Cbp*_*Co*_ / Total) across all assayed genes in the DNA data were close to equal (mean = 0.495). Thus, there was no strong mapping bias.

**Fig 7:**
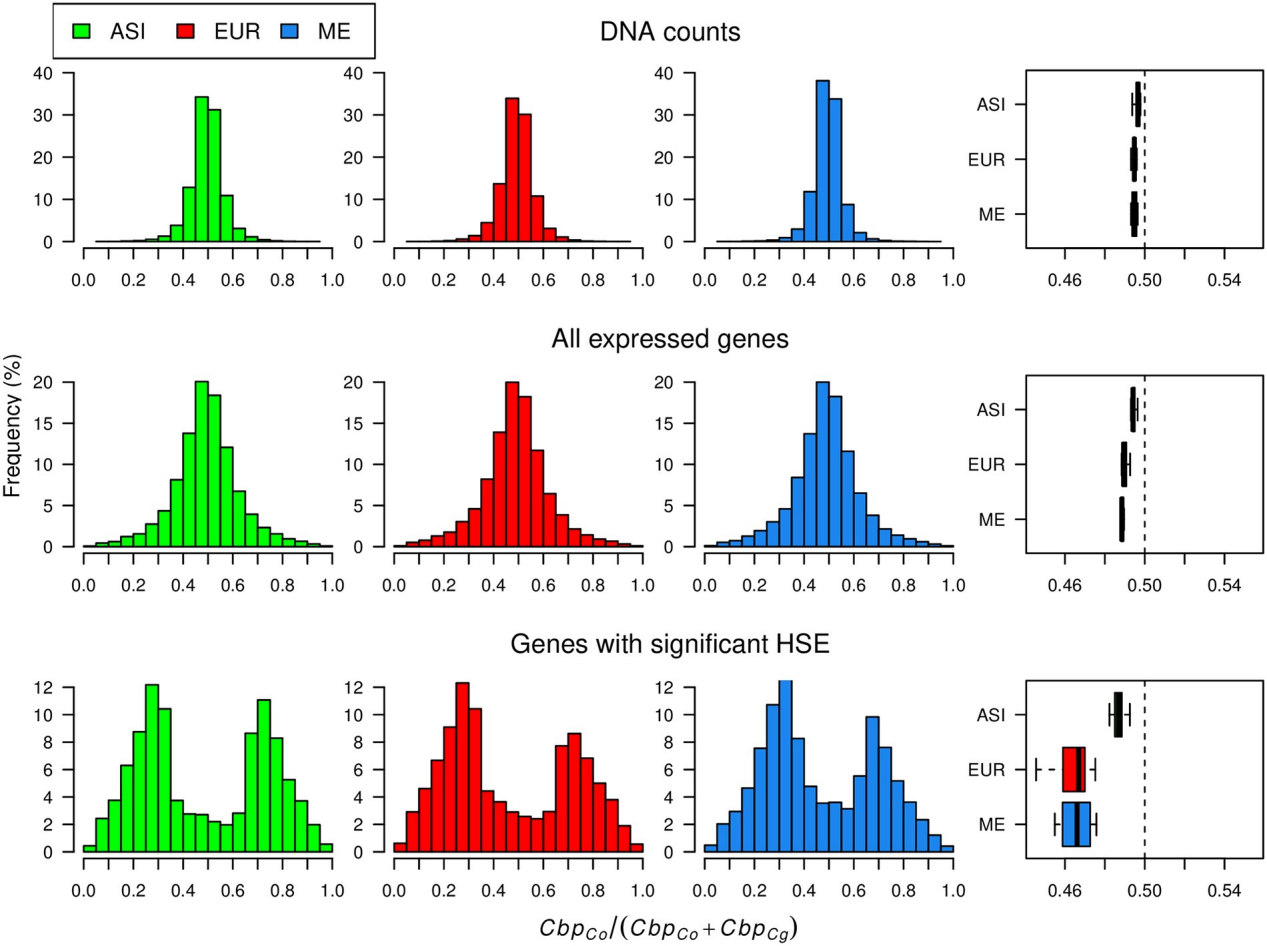
Distributions of expression ratios between the two subgenomes of *C. bursa-pastoris*. The homeologue specific expression (HSE) is estimated by the fraction the *Cbp*_*Co*_ subgenome relative to the total expression level. The upper part presents the distributions for DNA counts, the middle plots show the expression for all assayed gene and the lower plot shows only the distribution for genes with significant HSE. The histograms present the distribution of the allelic ratio, whereas the boxplots summarize these results with the grand mean for every sample. ASI, EUR, and ME indicate Asian, European and Middle Eastern populations, respectively.

We found some HSE variation among populations. The mean expression ratios for all genes were 0.494, 0.489, and 0.489 in the ASI, EUR, and ME accessions, respectively, and these mean ratios for genes with significant HSE were 0.487, 0.465, 0.468. The difference in mean ratio between EUR and ME was not significant, but both EUR and ME were significantly different from ASI (S11 Table). In addition, the distribution of expression ratios between the two subgenomes was right-skewed in EUR and ME, whereas in the ASI population, the distribution was more symmetrical (Fig 7). The difference between the populations was particularly evident in the grand mean values (Fig 7). Thus, the shift towards higher expression of the *Cbp*_*Cg*_ subgenome was more prominent in Europe and the Middle East than in Asia.

## Discussion

In the present study, we analyzed the genetic changes experienced by the recently formed allopolyploid *C. bursa-pastoris* since its founding, focusing on the evolutionary trajectories followed by its two subgenomes in demographically and genetically distinct populations from Europe, the Middle East, and Asia. The shift to selfing and polyploidy had a global impact on the species, resulting in a sharp reduction of the effective population size in all populations accompanied by relaxed selection and accumulation of deleterious mutations. However, the two subgenomes were not similarly affected, with the magnitude of the subgenome-specific differences depending on the population considered. The relative patterns of nucleotide diversity, genetic load, selection and gene expression between the two subgenomes in the European and the Middle Eastern populations were distinct from that observed in Asia. The differences between populations were further enhanced by post-speciation hybridization of *C. bursa-pastoris* with local parental lineages. Below, we discuss these global and local effects in more detail and their consequences for the history of the species.

But before this, a few words need to be said on the reliability of the phasing approach that was chosen here. Our results are based on the computationally phased (HapCUT) genomic data that was generated from the reads mapped to the *C. rubella* reference genome—the only reference genome available for the *Capsella* genus at the time of this study. This approach was possible due to the strict disomic inheritance of *C. bursa-pastoris* [36, 37]. Disomic inheritance and reproduction by selfing resulted in almost no variation within subgenomes and we therefore were able to treat our data as diploid. The major challenge of our approach was to reduce mapping bias in favor of the *Cbp*_*Cg*_ subgenome that is more closely related to *C. rubella* than the *Cbp*_*Co*_ subgenome [10]. To minimize this bias, we performed tolerant read mapping, stringent SNP filtering and even more stringent filtering of gene expression data. Our survey of the frequency of non-reference alleles, consistent decay of linkage disequilibrium with a distance, distribution of coverage and heterozygosity across the genome (see Material and methods), and almost an equal ratio between homeologous alleles in the DNA data, indicated that there were no major phasing errors. We also verified the sweeps and admixture results by analyzing only the phased fragments showing 100% consistency with fixed differences between parental species. Furthermore, we phased the same data using an alternative approach (HomeoRoq) and mapping our reads to the assembly of *C. bursa-pastoris* [37]. The two alternatively phased datasets were largely consistent in the separation between the subgenomes, with a perfect clustering between alternatively phased samples (HapCUT and HomeoRoq) in EUR and a clustering more influenced by phasing in ASI samples. However, even in that case, ASI samples still were more related to each other than to any other cluster and larger-scale grouping remained the same. Given that the assembly of *C. bursa-pastoris* we used for this validation was done with short reads and that the classification of the scaffolds between homeologues was based only on the coding part and allowed up to 25% of discrepancy between parental species [37], the congruence of the results is encouraging. Overall, we hence believe that our phasing approach did not lead to biases that could invalidate the main conclusions of our study.

### The effect of parental legacy is detectable even after ∼100,000 years of co-evolution of two initially different subgenomes

The genetic composition of parental species obviously has a strong impact on the evolution of subgenomes in allopolyploids. For example, the allopolyploid *Arabidopsis kamchatica* originated from the outcrossing diploids *Arabidopsis halleri* and *Arabidopsis lyrata* [46] that have rather similar effective population sizes and the two subgenomes of *A. kamchatica* have thus similar effective population sizes and levels of negative selection [47]. In contrast, the effective population size of the diploid outcrossing ancestor of *C. bursa-pastoris*, *C. grandiflora*, is ten times larger than that of its selfing ancestor *C. orientalis* [10]. Any analysis of the difference in effective population size between the subgenomes of *C. bursa-pastoris* or of their evolutionary trajectories must therefore account for this initial difference. After the bottleneck associated with the origin of *C. bursa-pastoris* and the reduction in *N*_*e*_ due to the shift to selfing [48], the effective population sizes of the two subgenomes are expected to progressively converge and decrease along the same trajectory.

While this was indeed the observed overall pattern, the trajectories followed by the two subgenomes in the three populations differed: in Europe the initial level was similar to that in the Middle East but higher than in Asia and the decline of *N*_*e*_ of the *Cbp*_*Cg*_ subgenome was delayed compared to the sudden decline experienced by the *Cbp*_*Co*_ subgenome. In contrast, in Asia the two subgenomes initially followed similar downwards trajectories but *N*_*e*_ increased again in both subgenomes at around 40,000 ya. In the diploid *C. orientalis*, there was a period of stasis followed by a steeper decline than in the tetraploid. The difference in demography across the three regions could indicate multiple origins of *C. bursa-pastoris* as suggested by Douglas et al. [10] and the difference between the diploid and the tetraploid could reflect a mixture of the population expansion experienced by the tetraploid and the buffering effect of tetraploidy against deleterious mutations.

There was a clearly noticeable difference between the two subgenomes in the number of accumulated deleterious mutations. Based on the strong differences in *N*_*e*_, one would expect the efficacy of selection to be much higher in *C. grandiflora* than in *C. orientalis* that has a much smaller *N*_*e*_ [49]. In the analysis of the genetic load, we indeed observed that *C. orientalis* had a higher proportion of deleterious mutations than *C. grandiflora*. Hence, the amount of genetic load most likely was different between the *Cbp*_*Cg*_ and *Cbp*_*Co*_ subgenomes of *C. bursa-pastoris* at the time of the species emergence. Interestingly, hundreds of thousands of generations of selfing did not totally erase the differences between the two subgenomes and, today, the *Cbp*_*Co*_ subgenome still accumulates more deleterious mutations than the *Cbp*_*Cg*_ subgenome. Despite being statistically significant, the difference between subgenomes is smaller than between *C. orientalis* and *C. grandiflora* suggesting a convergence of the two subgenomes, which is also confirmed by analyses of genetic load dispersion and gene expression regulation [50].

Everything else being equal, genomic redundancy in polyploids is expected to lead to relaxation of purifying selection, and to a higher rate of accumulation of deleterious mutations than in diploids. Douglas and coworkers [10], using forward simulations, concluded that indeed the *Cbp*_*Cg*_ subgenome showed an excess of deleterious mutations compared to what would have been expected from mating system shift alone and ascribed this excess of deleterious mutations to genomic redundancy. Our study also suggests that both effective population size and genomic redundancy contribute to the observed pattern. The proportion of derived deleterious mutations in both subgenomes is, with the exception of the *Cbp*_*Co*_ subgenome in the Asian population, lower than the same proportion in *C. orientalis* but much higher than in *C. grandiflora* where it is extremely low (Fig 5). Furthermore purifying selection is weaker on the *Cbp*_*Co*_ subgenome than on the *Cbp*_*Cg*_ subgenome. These data can be explained as follows. First, purifying selection on the *Cbp*_*Co*_ subgenome is stronger than in *C. orientalis* because the latter has a smaller effective population size than *C. bursa-pastoris*. Second, purifying selection is weaker on the *Cbp*_*Co*_ subgenome than on the *Cbp*_*Cg*_ because of genomic redundancy. Or, stated differently, because of its highest initial genetic quality the *Cbp*_*Cg*_ may be more sheltered from deleterious mutations than the *Cbp*_*Co*_ subgenome.

Nucleotide diversity also demonstrated the effect of parental legacy. The *Cbp*_*Cg*_ subgenome inherited from the more variable outcrosser *C. grandiflora* was still more diverse in all populations except the Asian one. The maintenance of part of the parental legacy in both cases suggests that, in spite of their initial differences, both subgenomes have experienced similar levels of fixation since the creation of the species. The Asian population is an exception in this regard because it was affected by secondary gene flow as discussed below. Variation in nucleotide diversity in the coding part of the genome also demonstrated similarity in the efficacy of purifying selection between the two subgenomes and their corresponding parental lineages, though the pattern in the ASI population was the reverse of that observed in the parental lineages. The effect of parental legacy in *C. bursa-pastoris* was also noted in Douglas et al. [10]. Thus, the effect of the genetic background of hybridizing species may not be as overwhelming as the effect of mating system but it still impacts the fate of the two subgenomes long after the species arose.

### Admixture with diploid species and/or multiple origins

Based on coalescent simulations and the amount of shared variation between *C. bursa-pastoris* and its parental species Douglas et al. [10] ruled out a single founder but noted that it would be difficult to estimate the exact number of founding lineages. Douglas et al. [10] did not consider hybridization but an earlier study detected gene flow from *C. rubella* to the European *C. bursa-pastoris* using 12 nuclear loci and a coalescent-based isolation-with-migration model [51], a result which is in agreement with the general occurrence of abundant trans-specific polymorphism in the *Arabidopsis* genus [52]. The present study adds two new twists to the story. First, our results indicate that shared polymorphisms were not symmetrical: namely, the *Cbp*_*Cg*_ subgenome showed signs of admixture with *C. rubella* in the EUR and ME populations, whereas the *Cbp*_*Co*_ subgenome was admixed with *C. orientalis* in ASI. Second, in both the whole genome and density trees, *C. orientalis* appears as derived from the *C. bursa-pastoris* *Cbp*_*Co*_ subgenome rather than the converse as one would have expected. No such anomaly was observed for *C. grandiflora* that, as expected, grouped at the root of the *C. bursa-pastoris* *Cbp*_*Cg*_ subgenome. These results could be explained by a mixture of multiple origins and a recent admixture. Multiple origins seem to be common in allotetraploids [27, 53, 54] and interploidy gene flow has already been inferred for *Capsella* [51] and other plant genera [55–57].

Our crossing results did not reject the possibility of ongoing gene flow between *C. bursa-pastoris* and parental lineages in both Europe and Asia. The distribution of European and Asian populations of *C. bursa-pastoris* partially overlap with the distribution ranges of *C. rubella* and *C. orientalis*, respectively (Fig 1A). The exact proportion of admixture remains unclear at this stage. Taken at face value, the strongest admixture was between the ASI *Cbp*_*Co*_ subgenome and *C. orientalis*. Considering the overlapping estimates of *f*-statistic and *HAPMIX*, the proportion of admixture between the ASI *Cbp*_*Co*_ subgenomes and *C. orientalis* was around 14%-23%. The admixture between the EUR *Cbp*_*Cg*_ subgenome and *C. rubella* was also strong, being around 8-20%. There were also signs of minor admixture in the ME population with both *C. orientalis* and *C. rubella*. This lack of a non-admixed population posed a problem of correct estimation of the proportion of admixture for both the ABBA-BABA and *HAPMIX* approaches.

In the ABBA-BABA test, departures from the assumptions can lead to incorrect interpretation of the results. We assumed monophyly of the three populations of *C. bursa-pastoris*, which may be wrong if these populations were of multiple origins. Thus, the observed shared polymorphism might be due to closer relatedness of our *C. orientalis* samples with the parent of the ASI population than with the parent of EUR and ME populations, but not admixture. Departures from the assumptions of the ABBA-BABA test can lead to under- or overestimated admixture. In the present case, some proportion of the variation shared between *P*_3_ and both *P*_1_ and *P*_2_ populations could be due to gene flow and not due to incomplete lineage sorting and this would lead to underestimating the amount of admixture. On the other hand, small *N*_*e*_ and recent divergence of the populations used in the test can inflate estimates of *D* [58]. Further, the behavior of *D* in tests involving both selfing and outcrossing species has not been assessed yet. The *D* statistics were significantly different from zero in all our comparisons suggesting that admixture did indeed occur in all populations of *C. bursa-pastoris*. The *f* statistic is considered less prone to be affected by these factors [58], and it was more reliable in our tests too. Its values were close to zero in the alternative combinations for the ABBA-BABA tests where we did not expect to find admixture, while *D* had high estimates (S12 Table). Thus, the *f* values are the closest to the real proportion of admixture we could obtain.

In *HAPMIX*, if the reference populations have non-negligible levels of admixture with each other, such that they have few differences, it will be difficult for *HAPMIX* to distinguish with which reference population the focal population is more likely to share ancestry, driving admixture probabilities to intermediate values. Therefore, we observed a discrepancy between the results of *HAPMIX* and ABBA-BABA in the estimates of admixture between the EUR *Cbp*_*Co*_ subgenome and *C. orientalis*. However, the results for the *Cbp*_*Cg*_ subgenome largely agreed between *HAPMIX* and ABBA-BABA and, together with the results by Slotte et al. [51] and our crossing experiment bolsters the hypothesis of admixture between *C. rubella* and *C. bursa-pastoris* in Europe. On balance, a scenario with a single origin of *C. bursa-pastoris* with later rampant admixture with *C. orientalis* in Asia and less extensive admixture with *C. rubella* in Europe is consistent with our data.

**Fig 8:**
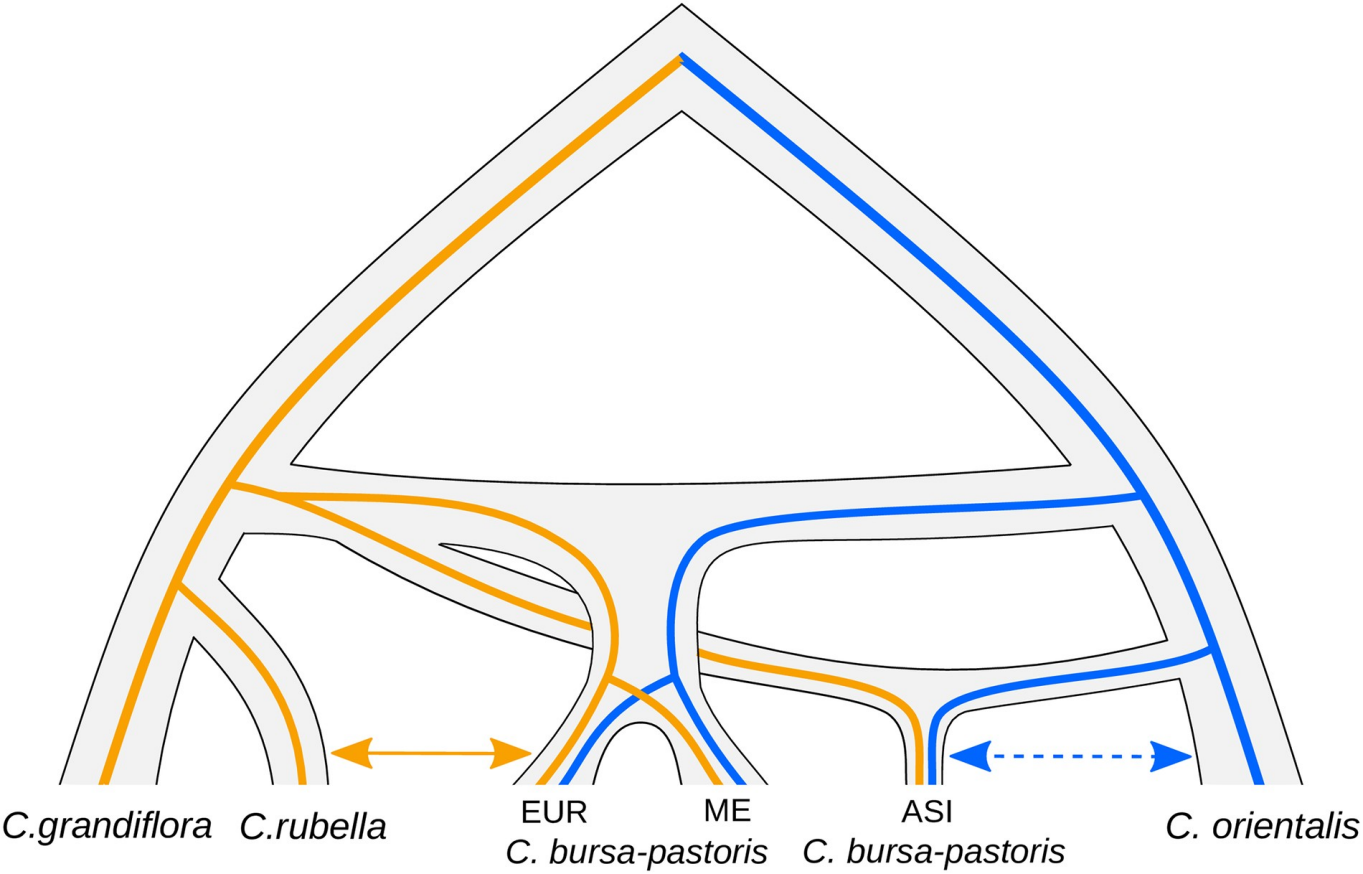
A tentative scenario of multiple origins of *C. bursa-pastoris*. The Asian population originated separately from other *C. bursa-pastoris* populations. There is gene flow between the European *C. bursa-pastoris* and *C. rubella* (solid arrow), and there may be gene flow between the Asian population and *C. orientalis* (dashed arrow). ASI, EUR, and ME indicate Asian, European and Middle Eastern populations of *C. bursa-pastoris*, respectively.

On the other hand, our results could also be obtained under a scenario of multiple origins. Such a scenario seems particularly likely if one looks at Fig 4, where the histories of the *Cbp*_*Co*_ and *Cbp*_*Cg*_ subgenomes are strikingly different. If we assume that *C. orientalis* and *C. grandiflora* are indeed parental lineages and there was no unknown parental lineage that went extinct, this picture can be only explained by a separate and more recent origin of the ASI population (Fig 8). However, the scenario of multiple origins and post-speciation admixture are not mutually exclusive. The signs of gene flow between EUR and *C. rubella* are still best explained by post-speciation admixture. The weak signs of admixture between *C. bursa-pastoris* and *C. orientalis* in EUR and ME are also difficult to fit into a scenario involving only multiple origins. A possibility is that these signs of admixture resulted from gene flow from ASI to EUR and ME within *C. bursa-pastoris*. The ASI population is more related to *C. orientalis* and the presence of its alleles in EUR and ME could be spuriously recognized as introgressed from ASI. Regardless of whether a single or a multiple origins scenario is the true one, our results demonstrate that the history of *C. bursa-pastoris* is far more complex than previously imagined.

### Weak subgenome-specific expression differences

Many allopolyploid species show subgenome expression bias, where one subgenome tends to be overexpressed relative to the other one [59–62]. This expression dominance is often observed in synthetic allopolyploids [63–66] and thus the major part of such preferential subgenome dominance is probably established immediately after allopolyploidization. The subgenome expression dominance is also suggested to be largely defined by parental expression differences [67, 68]. Contradictory results on patterns of subgenome specific expression in *C. bursa-pastoris* have been obtained so far. Douglas et al. [10] concluded that there is no strong homeologue expression bias and those few genes showing HSE could be explained by parental expression differences. However, genes with HSE do show a slight bias towards over-expression of the *Cbp*_*Cg*_ subgenome inherited from *C. grandiflora / rubella* lineage on the Figure 3B in Douglas et al. [10]. In contrast, Steige et al. [69] reported higher expression of the *Cbp*_*Co*_ subgenome inherited from *C. orientalis* in three accessions, and *Cbp*_*Cg*_ over-expression in a fourth one (CbpGR). Steige et al. [69] hypothesized that the over-expression of the *Cbp*_*Co*_ subgenome might be related to a higher number of transposable elements in this subgenome, but they did not find any evidence of this and could not explain the down-regulation of the *Cbp*_*Co*_ subgenome in the CbpGR accession and in the artificial hybrid between *C. rubella* and *C. orientalis*.

Considering the population histories of *C. bursa-pastoris* sheds some light on these discrepancies. The results of Douglas et al. [10] and Steige et al. [69] are consistent with the hypothesis that *cis*-regulatory differences between the *C. orientalis* and *C. grandiflora / rubella* genomes result in over-expression of the *Cbp*_*Cg*_ subgenome in a hybrid comprising both genomes. Thus, in the absence of other factors, the slight over-expression of the *Cbp*_*Cg*_ subgenome would be the default HSE pattern in *C. bursa-pastoris*. In accordance with this, we observed over-expression of the *Cbp*_*Cg*_ subgenome in the ME and EUR populations that are most likely the closest to the region of origin of *C. bursa-pastoris* [34]. The accessions that show over-expression of the *Cbp*_*Cg*_ subgenome in Douglas et al. [10] (SE14 from Sweden) and in Steige et al. [69] (CbpGR from Greece), as we now know belong to the EUR population [34]. Hence, their results are consistent with ours and expected if the HSE is defined primarily by the differences between the parental lineages. On the other hand, we observed that genes with HSE in the ASI population showed equal expression between the two subgenomes. The accessions showing over-expression of the *Cbp*_*Co*_ subgenome in Steige et al. [69] also mostly belong to the ASI population (CbpKMB and CbpGY, though not CbpDE that putatively originates from Germany). Thus, the Asian accessions show a HSE that differs from the default pattern. This difference can be caused by the selection preference for the *Cbp*_*Co*_ subgenome and/or by admixture with *C. orientalis* that enhanced the *cis*-regulatory elements of the *Cbp*_*Co*_ subgenome. The ASI population experienced a strong population bottleneck, so genetic drift played some role as well. These explanations need to be confirmed because HSE can be influenced by many factors (e.g. *trans*-regulatory elements, gene methylation, transposable elements), but it is clear that there are different directions of HSE in populations of *C. bursa-pastoris* and they are caused by the different evolutionary histories of those populations.

The reason we observed an equal expression between subgenomes in ASI, whereas Steige et al. [69] detected expression bias of the *Cbp*_*Co*_ subgenome for Asian samples, could also be due to different approaches in our analyses. First, we extracted RNA from seedlings, whereas Steige et al. [69] obtained RNA from leaves and flower buds. Variation in HSE for different tissues of *C. bursa-pastoris* is not characterized yet, so the *Cbp*_*Co*_ expression in seedlings may not be apparent yet. Second, we mapped reads to the *C. rubella* reference with masked polymorphism, whereas Steige et al. [69] used the reconstructed reference of an F1 hybrid between *C. orientalis* and *C. rubella*. The bias in our DNA data was not stronger than in Steige et al. [69], so which method is more appropriate remains to be found out.

### Conclusion

Three salient, and sometimes unexpected, features of the evolution of the tetraploid shepherd’s purse that emerged from the present study, are its complex origin and possible admixture with diploid relatives, the long-lasting effects of the difference between its two parental species, and the importance of demography in shaping its current genomic diversity. Hence, the present study suggests that understanding the evolution of tetraploid species without paying due attention to the historical and ecological backgrounds under which it occurred could be misleading.

## Materials and methods

### Sequence data

We obtained the whole genome sequences of 31 accessions of *C. bursa-pastoris* and the transcriptomes of 24 of these accessions. Transcriptome data used in this study was generated previously from seedling growth in the same growth chamber [35]. Whole genome DNA data consisted of 10 accessions sequenced previously [10] and 21 accessions sequenced in this study. New DNA samples were sequenced using the same technology as the downloaded ones (100-bp paired-end reads, Illumina HiSeq 2000 platform, SciLife, Stockholm, Sweden). The mean genomic coverage of *C. bursa-pastoris* samples was 47x. We also used the previously generated genomic data of 10 *C. orientalis* and 13 *C. grandiflora* samples [10]. For the analysis requiring an out-group, we used the whole genome assembly of *Neslia paniculata* [70]. Detailed information on the samples is provided in S1 Table.

### Genotype calling and phasing

DNA reads from each individual of *C. bursa-pastoris* were mapped to the *Capsella rubella* reference genome [70] and subsequently computationally phased the two subgenomes. We favored this approach, which has been already successfully implemented in [10], over mapping to two alternative genomes or read-sorting genotyping algorithms because the two subgenomes of *C. bursa-pastoris* are quite similar to each other (∼1-3% divergence), and alternative approaches would have large regions where reads cannot be assigned to the parent of origin [21, 54, 71, 72]. To account for the divergence from the reference and ensure minimal read-mapping bias between the two subgenomes, we performed tolerant read mapping using Stampy v1.0.22 [73] with the substitution rate set to 0.025. Potential PCR duplicates were marked using Picard Tools 1.115 (http://picard.sourceforge.net) and were ignored during genotyping. Genotypes were called using *HaplotypeCaller* from the Genome Analysis Tool Kit (GATK) v3.5 in the GVCF diploid mode and heterozygosity set to 0.015 [74]. Genotypes were filtered for depth between 6 and 100 reads (the 5th and 99th coverage percentiles, respectively) to remove low confidence genotypes due to low coverage or due to their location in repetitive regions and paralogs, which usually have abnormally high coverage. This approach produced a VCF file containing all called sites. This VCF was used in the analyses requiring both polymorphic and monomorphic sites for correct estimates. To obtain a set of SNPs with the highest confidence possible, we generated another VCF file that contained only polymorphic sites and applied more stringent filtering. We set to no-call all sites that met the following criteria: *MQ* < *30*, *SOR* > *4*, *QD* < *2*, *FS* > *60*, *MQRankSum* < -*20*, *ReadPosRankSum* < -*10*, *ReadPosRankSum* > *10*. These filtering criteria were defined following GATK Best Practices [75] with some adjustment guided by the obtained distributions of the GATK annotation scores (S16 Fig).

To phase the *C. bursa-pastoris* subgenomes, we run HapCUT version 0.7 [76] on each sample from the VCF with the stringently filtered SNPs. The phased haplotype fragments were then concatenated into two sequences descended from *C. grandiflora* and *C. orientalis* (S17 Fig). The origin of haplotypes in HapCUT fragments was defined using sites with fixed heterozygotes in *C. bursa-pastoris* and fixed differences between the parental lineages. The fixed differences were defined as fixed between 10 *C. orientalis* and 13 *C. grandiflora* with maximum 20% of missing data per position. Fragments that had small (< 2 sites) or no overlap with variation in *C. grandiflora* and *C. orientalis* as well as those that looked chimeric (prevailing phasing state was supported by less than 90% of sites) were set to missing data (S18 Fig). Additionally, we also set to missing the sites that were defined as not real variants or not heterozygous by HapCUT (flagged with *FV*). We checked that the distribution of the length of phased haplotype blocks and the proportion of introduced missing data across samples were not strongly different (S19 Fig). HapCUT phasing produced the alignment that had only heterozygous sites and removed all the sites that were non-variant within but variable between individuals. We restored this inter-individual variation by introducing the same proportion of missing data into non-variant sites as it was introduced to heterozygous sites during the phasing. Similarly, we merged the phased SNPs dataset with non-polymorphic sites from the whole genome data to keep the level of heterozygosity as in the unphased data.

To ensure that there was no sign of a general bias towards the *Cbp*_*Cg*_ subgenome, which is less divergent from the reference genome than *Cbp*_*Co*_, we checked that there was no strong bias towards the reference allele in the VCF file. The bias was only ∼4% and it should not affect alleles calling in heterozygous sites even at the lowest coverage. We also surveyed the level of heterozygosity and coverage along the genome and showed that there was no regional dropout of *Cbp*_*Co*_ haplotypes (S20 Fig). In addition, the accuracy of phasing was assessed with the linkage disequilibrium (LD) variation between and within the subgenomes. From each subgenome, we randomly sampled 1000 SNPs on each chromosome (the principal SNPs) and calculated *r*^2^ between them and the next 1000 SNPs. These SNPs could originate from the same genome as the principal SNPs or from the opposite subgenome. We only considered SNP pairs where at least 5 samples had genotypes for both SNPs. As a baseline, we also calculated LD in the same way for a genome containing a random mixture of SNPs from both subgenomes. LD gradually decayed with increasing distance between SNPs within homeologues but not between them or when SNPs from both subgenomes were randomly mixed (S3D Fig), further indicating that there was no major phasing error in our data.

To verify our phasing procedure with an alternative phasing method, we utilized the HomeoRoq pipeline [77] that was successfully used to phase transcriptomic and genomic data in allopolyploid *Arabidopsis kamchatica* [78, 79]. While HapCUT relies on the read-back phasing of the reads mapped to one reference, HomeoRoq classifies the reads mapped to two references corresponding to the subgenomes. As the two references, we used the recently published assembly of *C. bursa-pastoris* [37]. We independently mapped our reads to the each assembled subgenome. Then we classified reads with the HomeoRoq to keep only unique and common reads, and to remove unclassified reads from SAM files. These SAM files were processed the same way as described above to obtain SNPs. Next, we aligned the main dataset with the HomeoRoq phased data, according to the alignment between the *C. rubella* reference and the *C. bursa-pastoris* assembly. The latter was performed by aligning each subgenome of *C. bursa-pastoris* to *C. rubella* with the *LASTZ* program [80] following the procedure described in [10]. Finally, we analyzed the clustering of the samples phased with the two different methods using a sliding window phylogenetic analysis as described below.

The sequences of *C. grandiflora* and *C. orientalis* were created using the GVCF files produced by Douglas et al. [10]. The variants were called as described above with additional filtering for fixed differences between the two species. For some of the analyses, where the software was not able to treat heterozygous genotypes properly, we pseudo-phased the sequences of *C. grandiflora* and *C. orientalis* by randomizing alleles in heterozygous genotypes.

The data-sets in all the analyses comprised the alignment of phased *C. bursa-pastoris* sequences, *C. grandiflora*, *C. orientalis*, *C. rubella* (the reference sequence) and *N. paniculata*. This alignment was filtered for missing data such that genomic positions with more than 80% of missing genotypes were removed. We also removed the repetitive sequences as annotated in Slotte et al. [70] and pericentromeric regions that we delineated based on the density of repetitive regions and missing data. The final data-set had even proportion of missing data in the three populations of *C. bursa-pastoris* and diploid species (S21 Fig).

### Reconstruction of the ancestral sequences

Several analyses presented in this paper required polarized sequence data. The most common approach to polarizing the alleles is to use an outgroup. However, the alignment of *Capsella* species and *N. paniculata*, the nearest outgroup with a whole genome sequence available, resulted in substantial reduction of the dataset due to missing data. To overcome this drawback, as well as to track mutations’ origin on the phylogenetic branches, we reconstructed ancestral sequences for major phylogenetic splits. The reconstruction was performed on the tree that was assumed to represent a true history of the *Capsella* species (S22 Fig) using the empirical Bayes joint reconstruction method implemented in PAML v4.6 [81].

### Population differentiation

To assess the degree of differentiation among populations for the two subgenomes, we estimated absolute divergence (*Dxy*) and nucleotide diversity (*π*) of the phased genomes using a sliding window approach. The estimates were calculated on non-overlapping 100 Kb windows using the *EggLib* Python module [82]. The *p*-values for the difference in mean values were estimated using 10,000 bootstrap resamples from 100 Kb windows.

### Temporal change in *N*_*e*_

We reconstructed changes of *N*_*e*_ over time with both *PSMC* [83] and *SMC++* [39]. We first masked potential CpG islands and all nonsynonymous sites in the genome to avoid bias caused by variation in mutation rates or selective effects. We randomly paired haplotypes for estimation in *C. orientalis* and did the same for estimations based on the two subgenomes of *C. bursa-pastoris*. *SMC++* was run on all samples from a population, with default parameter settings. For *PSMC* runs, we set parameters to “-N25 -t15 -r5 -p 4+25*2+4+6”. Variation in *N*_*e*_ was estimated using 100 bootstrap replicates and three different pairs. We chose a mutation rate equal to the mutation rate of *A. thaliana*, *μ* = 7 × 10^−9^ per site per generation [84] and a generation time of 1 year for all *Capsella* species.

### Phylogenomic analyses

We reconstructed a whole genome phylogeny to explore the relationship between the phased subgenomes of the three populations of *C. bursa-pastoris* as well as its parental species. To investigate the local phylogenetic relationships along the genome, we also conducted a sliding window phylogenetic analysis using non-overlapping 100 Kb windows. In both analyses, phylogenetic trees were reconstructed using the neighbor-joining algorithm and absolute genetic distance in R package *ape* [85]. Additionally, a whole genome phylogenetic tree was also reconstructed using the maximum-likelihood approach with the GTRGAMMA model and 100 bootstrap replicates in *RAxML* v8.2.4 [86] (S23 Fig). The trees from the sliding window analysis were described by counting the frequency of monophyly of different groups with the Newick Utilities [87]. The variation in topology across the genome was also described using topology weighting implemented in *TWISST* [40]. The weighting was estimated for 100 SNPs windows where each sample was genotyped for at least 50 SNPs. To test for the difference in mean topology weighting, we fitted the generalized linear model with a binomial distribution and performed multiple comparisons for the contrasts of interest with the *glht* function from the *multcomp* library in *R* [88].

### Tests for admixture

To evaluate the presence of admixture between the parental species and *C. bursa-pastoris*, we calculated the ABBA-BABA based statistics, *D*, an estimate of departure from incomplete lineage sorting, and *f*, an estimate of admixture proportion [41, 42]. These statistics and their significance, which was estimated with a 1Mb block jackknife method, were calculated from population allele frequencies with scripts from Martin et al. [89]. We also used *HAPMIX* [90] to infer haplotype blocks of admixture with the diploid *C. grandiflora*, *C. rubella*, and *C. orientalis* into the three populations of *C. bursa-pastoris* for each phased subgenome. We removed sites with more than 20% missing data for each population. The remaining missing data was imputed for the parental populations used in each analysis. As this method determines the probability of ancestry from a diploid progenitor population relative to a non-admixed *C. bursa-pastoris* subgenome population, we defined regions of the subgenomes as putatively admixed if the probability of ancestry from the progenitor diploid was greater than 50%.

### Selection tests

To search for selective sweeps, we used SweepFinder2 [91]. SweepFinder2 was run on the data-set that besides polarized SNPs also included fixed derived alleles. This enables accounting for variation in mutation rate along the genome and increases power to detect sweeps [92]. The critical composite likelihood ratio (CLR) values were determined using a 1% cut-off of the CLR values estimated in 100 simulations under a standard neutral model. The simulations were performed with *fastsimcoal2* [93]. We assumed a mutation rate of 7 × 10^−9^ per site per generation, the population effective sizes for every population and subgenome were inferred from the *θ* values approximated by genetic diversity (*π*), and the average recombination rate was estimated using LDhelmet v1.7 [94]. In addition, we estimated the ratio between nucleotide diversity at 0-fold (*π*_0_) and 4-fold degenerate sites (*π*_4_) in 5-6 samples with the lowest amount of missing data in each group.

### Genetic load estimation

To identify differences in genetic load between populations of *C. bursa-pastoris* (as well as to assess the effect of selfing on accumulation of deleterious mutations), we classified mutations into tolerated and deleterious ones using *SIFT4G* [44]. We built the *SIFT4G* *Capsella rubella* reference partition database and used it to annotate our SNPs dataset. Then we analyzed the frequencies of tolerated and deleterious mutations. We also verified this analysis by using *A. thaliana* *SIFT4G* database and annotating *C. bursa-pastoris* according to the alignment between the two species. This verification was performed to make sure that the observed results were not due to a reference bias because *C. rubella* is closer to *C. grandiflora* than to *C. orientalis*. To get only the annotation of the mutations that occurred after speciation of *C. bursa-pastoris*, we polarized the mutations with the reconstructed ancestral sequences (see above) and analyzed only derived mutations. We verified this polarization by analyzing only species(subgenome)-specific mutation (e.g. mutations unique to *C. bursa-pastoris* *Cbp*_*Co*_ subgenome, *C. bursa-pastoris* *Cbp*_*Cg*_ subgenome, *C. orientalis*, *C. grandiflora*, and *C. rubella*) (S24 Fig). All the counts were presented relative to the total number of annotated sites to avoid bias caused by variation in missing data between samples. The means of the genetic load were compared using the generalized linear model as we did for the topology weighting except that here we used a quasibinomial distribution due to overdispersion.

### Homeolog-specific expression analyses

Mapping of RNA-Seq reads to the *C. rubella* reference genome was conducted similarly to the mapping of DNA data using Stampy v1.0.22 [73] with the substitution rate set to 0.025. Although potential PCR duplicates are usually not removed from RNA-Seq data, for the allele-specific expression analysis removing duplicates is recommended [95]. We marked duplicates with Picard Tools 1.115 and did not use them during the genotyping and homeolog-specific expression assessment. Variants were called using *HaplotypeCaller* (GATK) with heterozygosity set to 0.015, and minimum Phred-scaled call confidence of 20.0, and minimum Phred-scaled emit confidence of 20.0 as recommended for RNA-Seq data in GATK Best Practices [75]. Among the obtained polymorphic sites those that had *MQ* < *30.00*, *QD* < *2.00*, *FS* > *30.000* were filtered out. Calls with coverage of fewer than 10 reads were also excluded. Alleles counting was carried out using *ASEReadCounter* from GATK.

Homeolog-specific expression was assessed within the statistical framework developed by Skelly et al. [45]. This framework uses a Markov chain Monte Carlo (MCMC) method for parameter estimation and incorporates information from both RNA and DNA data to exclude highly biased SNPs and calibrate for the noise in read counts due to statistical sampling and technical variability. First, we used DNA data to identify and remove SNPs that strongly deviated from the 0.5 mapping ratio. Second, we estimated the variation in allele counts using unbiased SNPs in the DNA data. Next, we fitted an RNA model using parameter estimated from DNA data in the previous step. Finally, we calculated a Bayesian analog of false discovery rate (FDR) with a posterior probability of homeologue specific expression (HSE) > 0.99 and defined genes with significant HSE given the estimated FDR. All inferences were performed using 200,000 MCMC iterations with burn-in of 20,000 and thin interval of 100. Each model was run three times with different starting parameters to verify convergence.

### Data access

DNA sequence data generated for 21 accessions of *C. bursa-pastoris* is submitted to the NCBI database under the sequence read archive number SRP126886. Previously generated DNA sequence data for 10 accessions of *C. bursa-pastoris*, 10 accessions *C. orientalis* and 13 accessions *C. grandiflora* is available in the NCBI (SRP050328, SRP041585, SRP044121). RNA-Seq data is also available in the NCBI (SRA320558). Both phased and unphased SNPs, phylogenetic trees, reconstructed ancestral sequences, estimates of *π* and *Dxy* with sliding window approach, results of *PSMC* and *SMC++*, *SIFT4G* annotations, CLR estimates of *sweepFinder2*, *TWISST* and *HAPMIX* outputs, homeologue-specific gene expression values are deposited to the Open Science Framework Repository (DOI: 10.17605/OSF.IO/5VC34) [96].

## Supporting information

S1 Fig Phylogenetic relationship reconstructed for each subgenome separately. **A** and **B**: Density tree visualizing 1002 NJ trees reconstructed with 100 Kb sliding windows for the *Cbp*_*Cg*_ and *Cbp*_*Co*_ subgenomes, respectively. **C** and **D**: Whole genome NJ tree showing the absolute divergence between different populations of *C. bursa-pastoris* for the *Cbp*_*Cg*_ and *Cbp*_*Co*_ subgenomes, respectively. The root *N. paniculata* is not shown. ASI, EUR ME, CO, CG, CR indicate Asian, European and Middle Eastern populations of *C. bursa-pastoris*, *C. orientalis*, *C. grandiflora*, and *C. rubella*, respectively.
(PDF)

S2 Fig Frequency of monophyly of different groups. ASI, EUR and ME are the three populations of *C. bursa-pastoris* (Cbp) with Co and Cg indicating two subgenomes. CO, CG, CR are short forms for *C. orientalis*, *C. grandiflora*, and *C. rubella* respectively.
(PDF)

S3 Fig Comparison of linkage disequilibrium decay within and between subgenomes. Mean LD was assessed for 100 window bins (each window 1000 SNP wide) within the **A)** *Cbp*_*Cg*_ and **B)** *Cbp*_*Co*_ subgenomes, **C)** between the *Cbp*_*Cg*_ and *Cbp*_*Co*_ subgenomes, and **D)** among SNPs randomly sampled from either subgenome. ASI, EUR and ME are the three populations of *C. bursa-pastoris*.
(PDF)

S4 Fig Overlap in SNPs called with GATK, HomeoRoq, and HapCUT phasing. GATK SNPs include all high-quality unphased SNPs. HapCUT SNPs include all successfully phased GATK SNPs. Overlap with HomeoRoq was possible to access only for positions that were aligned between the *C. rubella* reference and the *C. bursa-pastoris* assembly.
(PDF)

S5 Fig Phylogenetic relationship reconstructed for 10 K windows in the alternatively phased datasets. The trees were reconstructed with the neighbor-joining algorithm and absolute genetic distance for non-overlapping 10 K windows with minimum 1 K complete sites (88 trees). HomeoRoq phased samples have _A and _B in their names to indicate *Cbp*_*Cg*_ and *Cbp*_*Co*_ subgenomes, respectively, whereas samples phased with HapCUT are marked with _Cg and _Co for the corresponding subgenomes. ASI, EUR ME indicate Asian, European and Middle Eastern populations of *C. bursa-pastoris*, respectively.
(PDF)

S6 Fig Phylogenetic relationship reconstructed for 1 K windows in the alternatively phased datasets. The trees were reconstructed with the neighbor-joining algorithm and absolute genetic distance for non-overlapping 1 K windows with minimum 100 complete sites (784 trees). HomeoRoq phased samples have _A and _B in their names to indicate *Cbp*_*Cg*_ and *Cbp*_*Co*_ subgenomes, respectively, whereas samples phased with HapCUT are marked with _Cg and _Co for the corresponding subgenomes. ASI, EUR ME indicate Asian, European and Middle Eastern populations of *C. bursa-pastoris*, respectively.
(PDF)

S7 Fig Phylogenetic trees that failed the subgenomes mutual monophyly test in 1 K sliding windows for the alternatively phased datasets. The trees were reconstructed with the neighbor-joining algorithm and absolute genetic distance for non-overlapping 1 K windows with minimum 100 complete sites. Trees were tested for mutual monophyly of both the *Cbp*_*Cg*_ and *Cbp*_*Co*_ subgenomes. There were 132 trees that failed this test. HomeoRoq phased samples have _A and _B in their names to indicate *Cbp*_*Cg*_ and *Cbp*_*Co*_ subgenomes, respectively, whereas samples phased with HapCUT are marked with _Cg and _Co for the corresponding subgenomes. ASI, EUR ME indicate Asian, European and Middle Eastern populations of *C. bursa-pastoris*, respectively.
(PDF)

S8 Fig Genetic distance (Dxy) between the data analyzed in this study and the assembly of *C. bursa-pastoris* by Kasianov et al. (2017). The corresponding subgenomes are marked with Cg and Co. Estimates were assessed for 100 K windows on SNPs data and scaled to whole genome values.
(PDF)

S9 Fig Phylogenetic relationship of the alternatively phased datasets with parental species. The tree was reconstructed with the neighbor-joining algorithm and absolute genetic distance for 800 K SNPs. HomeoRoq phased samples have _A and _B in their names to indicate *Cbp*_*Cg*_ and *Cbp*_*Co*_ subgenomes, respectively, whereas samples phased with HapCUT are marked with _Cg and _Co for the corresponding subgenomes. The reference sequences of the assembled *C. bursa-pastoris* are named with TARG. CO, CG, ASI, EUR, ME indicate *C. orientalis*, and *C. grandiflora*, and Asian, European and Middle Eastern populations of *C. bursa-pastoris*, respectively.
(PDF)

S10 Fig Variation in nucleotide diversity (*π*) between subgenomes of *C. bursa-pastoris* in HomeoRoq phased data. The distributions of *π* were estimated from 797,806 SNPs using 100 Kb sliding windows and scaled to whole genome scale. Co and Cg indicate *C. orientalis* and *C. grandiflora/rubella* descendant subgenomes, respectively. ASI, EUR, and ME correspond to Asian, European and Middle Eastern populations of *C. bursa-pastoris*, respectively.
(PDF)

S11 Fig Population size histories of *C. bursa-pastoris* and its parental species estimated with *PSMC* and *SMC++*. Population sizes were inferred using whole-genome sequences from six randomly chosen haplotypes per population with PSMC and all haplotypes with SMC++. The estimates for PSMC and SMC++ are designated with different line thickness. Co and Cg specify subgenomes of *C. bursa-pastoris* and corresponding parental species in the CO & CG plot. ASI, EUR, ME, CO & CG indicate Asian, European and Middle Eastern populations of *C. bursa-pastoris*, and *C. orientalis* and *C. grandiflora*, respectively. The axes are in log scale and the most recent time, where PSMC is less reliable is excluded.
(PDF)

S12 Fig Topology weighting of the three populations of *C. bursa-pastoris*, *C. orientalis*, and *C. grandiflora*. **A**. Fifteen possible rooted topologies for the three groups of *C. bursa-pastoris* in one subgenome and corresponding parental species. The topologies are grouped into five main groups. Co and Cg indicate the two subgenomes. ASI, EUR ME, CO, CG, N indicate Asian, European and Middle Eastern populations of *C. bursa-pastoris*, *C. orientalis*, *C. grandiflora*, and *N. paniculata*, respectively. **B**. Topology weightings for 100 SNP windows plotted along 8 main scaffolds with loess smoothing (span = 1Mb). The tentative centromeric regions are shaded. **C**. Average weighting for the five main topology groups.
(PDF)

S13 Fig Bar plots of *HAPMIX* admixture probabilities for major scaffolds (chromosomes) of *C. bursa-pastoris*. **A**. Estimated admixture between *C. rubella* (dark red regions) and the European Cg subgenome, relative to the Middle Eastern Cg subgenome (green regions). **B**. Estimated admixture between *C. orientalis* (orange regions) and the European Co subgenome, relative to the Middle Eastern Co subgenome (green regions). The limits of centromeric regions are indicated by vertical dashed lines.
(PDF)

S14 Fig Nucleotide diversity (*π*) in the coding part of the genome. **A**. Nucleotide diversity in 0-fold (*π*_0_). **B**. Nucleotide diversity in 4-fold sites (*π*_4_). **C**. The ratio between *π*_0_ and *π*_4_. ASI, EUR and ME are the three differentiated populations of *C. bursa-pastoris*. Co and Cg indicate corresponding subgenomes. CO and CG are short forms for *C. orientalis* and *C. grandiflora*, respectively.
(PDF)

S15 Fig Selective sweep differences between populations of *C. bursa-pastoris* in the most stringently filtered data. **A**. The analysis was performed on the fragments that were 100% consistent in their phasing state with parental species. **B**. For ease of comparison, we also duplicate Fig 6 that shows the results for the data phased with 90% consistency with the parental species. The 0.01 significance level defined with data simulated under a standard neutral model is depicted with dashed lines. Pericentromeric regions are removed. Only eight major scaffolds (chromosomes) are shown. Selective sweeps are detected with the composite likelihood ratio statistics (CLR) along the *Cbp*_*Co*_ and *Cbp*_*Cg*_ subgenomes in Asian (ASI), European (EUR) and Middle Eastern (ME) populations.
(PDF)

S16 Fig Distribution of GATK annotation scores. The vertical lines indicate the used cut-offs. DP—combined depth per SNP across samples. QD—variant confidence standardized by depth. MQ—Mapping quality of a SNP. FS—strand bias in support for REF vs ALT allele calls. SOR—sequencing bias in which one DNA strand is favored over the other. MQRankSum—rank sum test for mapping qualities of REF vs. ALT reads. ReadPosRankSum—indicates if all the reads supporting a SNP call tend to be near the end of a read. Indels were not used in this study.
(PDF)

S17 Fig Phasing scheme used to phase genomic data of *C. bursa-pastoris* in this study. We were able to phaseheterozygous sites that are fixed between the *Cbp*_*Co*_ and *Cbp*_*Cg*_ subgenomes. The level of diversity within each subgenome is extremely low, so such variation as at the position 7 was ignored. HapCUT was able to phase regions with enough SNPs density to get overlapping reads with polymorphic sites (orange reads). However, there were gaps between these phased haplotype blocks. To assemble these blocks, we compared them to fixed differences in parental species, and if there were more than 10% discrepancy sites, we considered such block as chimeric and replaced it with Ns. An example of a chimeric block is the block between positions 3 and 4. This filtering resulted in lowering the number of polymorphic sites in the data. To compensate for this, we also randomly introduced missing data in non-polymorphic regions such as in the regions between position 4 and 5. This allowed us to maintain the same level of heterozygosity in the phased data as in the unphased one.
(PDF)

S18 Fig Example of the distribution of phasing states of haplotype blocks emitted by HapCUT. The blocks in the shaded area were considered chimeric and were discarded from the data. All samples had a rather similar distribution as shown in this figure. “Same” means that the alleles were phased in accord with the order Co-Cg; “reverse” indicated the opposite pattern Cg-Co. Thus, 1.0 indicates that all sites are phased as Co-Cg, and 0.0 means that all sites are in the reverse state Cg-Co. 0.5 means that half of alleles are phased as Co-Cg and the other half as Cg-Co.
(PDF)

S19 Fig Variation in the length of phased fragments and proportion of unsuccessfully phased positions across samples. Mean and median of the fragment length distribution in each sample are shown in absolute numbers. The introduced missing data (Ns) is presented relative to the total number of heterozygous sites that were used for phasing. ASI, EUR and ME accessions are colored by green, red, and blue, respectively.
(PDF)

S20 Fig Verification of non-biased genotyping of both subgenomes of *C. bursa-pastoris* with the distribution of heterozygosity and coverage across the genome. All values have been estimated with sliding windows of 100 K genomic positions on phased data. Heterozygosity is shown in relative numbers, while the coverage is in absolute numbers. The regions without the data mostly correspond to pericentromeric regions that were excluded from the data.
(PDF)

S21 Fig Variation in the proportion of missing data in populations of *C. bursa-pastoris* and diploid species. All values have been estimated with sliding windows of 100 K genomic positions on the final dataset that was used in the analyses. ASI, EUR and ME are the three populations of *C. bursa-pastoris*. CO and CG are short forms for *C. orientalis* and *C. grandiflora*, respectively. The regions without the data mostly correspond to pericentromeric regions that were excluded from the data.
(PDF)

S22 Fig Tree presumably reflecting the true history of the populations of *C. bursa-pastoris* and parental species. The original whole genome phylogenetic tree was distorted by gene flow between *C. orientalis* and *C. bursa-pastoris* in Asia or different origin of the ASI population. Therefore, we reconstructed an artificial tree that presumably reflects the true history. The clades of *C. bursa-pastoris* within each subgenome were reconstructed from a neighbor-joining tree of only *C. bursa-pastoris*. The parental species *C. orientalis*, *C. grandiflora* were then placed as basal to each of the corresponding subgenomes. Branch length was discarded from the tree. Red and blue dots indicate the nodes for which the ancestral sequences were reconstructed.
(PDF)

S23 Fig Maximum likelihood phylogenetic tree of two subgenomes of *C. bursa-pastoris* and its parental species. The tree was reconstructed for the SNPs data and the divergence axis is scaled to the whole genome values. The bootstrap values of 100 replicates are shown only for the major clades.
(PDF)

S24 Fig Genetic load in the subgenomes of *C. bursa-pastoris* and its parental species for species-specific mutations. The proportion of deleterious nonsynonymous changes was estimated with SIFT4G on species-specific mutations that are unique alleles for *C. orientalis*, *C. grandiflora* and the two subgenomes of *C. bursa-pastoris*. The left plot shows the results obtained with *C. rubella* database, the right plot was obtained with *A. thaliana* database. Co and Cg are the two subgenomes of *C. bursa-pastoris*. ASI, EUR, ME, CO, CG indicate Asian, European and Middle Eastern populations of *C. bursa-pastoris*, and parental species *C. orientalis* and *C. grandiflora*, respectively.
(PDF)

S1 Table Sequencing and phasing information. (PDF)

S2 Table Nucleotide diversity *π* and absolute divergence D_xy_ between different subgenomes of *C. bursa-pastoris* populations and its parental species. (PDF)

S3 Table Multiple comparisons for the generalized linear model of the topology weighting of the *Cbp*_*Co*_ subgenome of *C. bursa-pastoris* and *C. orientalis*. (PDF)

S4 Table Multiple comparisons for the generalized linear model of the topology weighting of the *Cbp*_*Cg*_ subgenome of *C. bursa-pastoris* and *C. rubella*. (PDF)

S5 Table Multiple comparisons for the generalized linear model of the topology weighting of the *Cbp*_*Cg*_ subgenome of *C. bursa-pastoris* and *C. grandiflora*. (PDF)

S6 Table Results of the ABBA-BABA tests assessing the admixture between *C. bursa-pastoris* and *C. orientalis*, *C. rubella* for the unphased data. (PDF)

S7 Table Results of the ABBA-BABA tests assessing the admixture between *C. bursa-pastoris* and *C. orientalis*, *C. rubella* for the complete phased data. (PDF)

S8 Table Summary of the probabilities and proportions of admixture from *HAPMIX* analyses of each of the *C. bursa-pastoris* subgenomes. (PDF)

S9 Table Multiple comparisons for the generalized linear model on the genetic load estimated with the *C. rubella* *SIFT4G* database. (PDF)

S10 Table Multiple comparisons for the generalized linear model on the genetic load estimated with the *A. thaliana* *SIFT4G* database. (PDF)

S11 Table Multiple comparisons for the generalized linear model testing for the expression difference between populations. (PDF)

S12 Table Alternative combinations used in the ABBA-BABA tests. (PDF)

S1 Appendix Artificial crosses. (PDF)
