# Supplementary material for: Parental legacy, demography, and admixture influenced the evolution of the two subgenomes of the tetraploid Capsella bursa-pastoris (Brassicaceae)
Source: PLoS Genet. 2019 Feb 15;15(2):e1007949. doi: 10.1371/journal.pgen.1007949 (PMC6395008; doi:10.1371/journal.pgen.1007949)
Supplement: S1 Fig — A and B: Density tree visualizing 1002 NJ trees reconstructed with 100 Kb sliding windows for the CbpCg and CbpCo subgenomes, respectively. C and D: Whole genome NJ tree showing the absolute divergence between different populations of C. bursa-pastoris for the CbpCg and CbpCo subgenomes, respectively. The root N. paniculata is not shown. ASI, EUR ME, CO, CG, CR indicate Asian, European and Middle Eastern populations of C. bursa-pastoris, C. orientalis, C. grandiflora, and C. rubella, respectively. (PDF) [file pgen.1007949.s001.pdf]

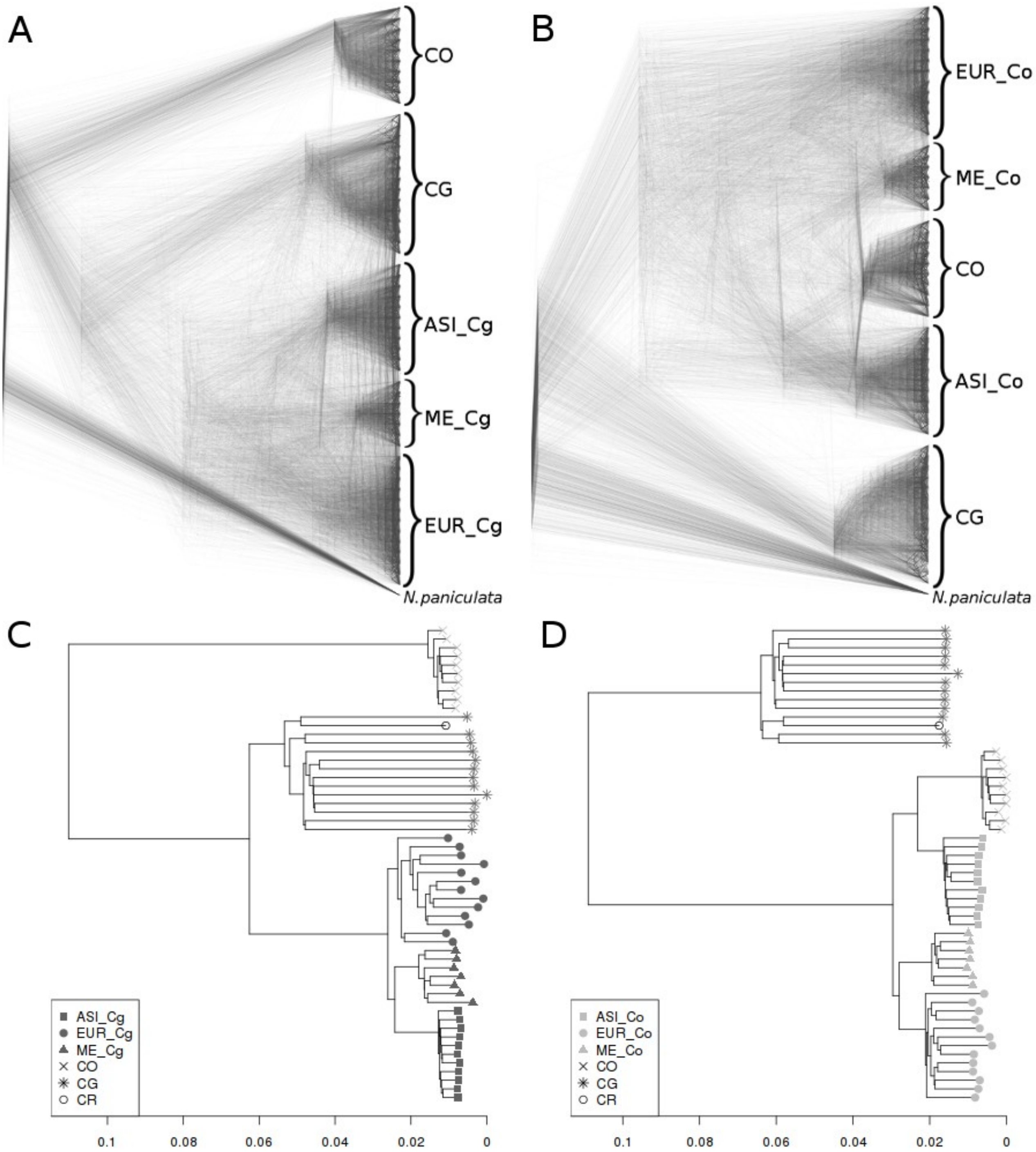

S1 Figure. Phylogenetic relationship reconstructed for each subgenome separately. **A** and **B**: Density tree visualizing 1002 NJ trees reconstructed with 100 Kb sliding windows for the Cbp_{Cg} and Cbp_{Co} subgenomes, respectively. **C** and **D**: Whole genome NJ tree showing the absolute divergence between different populations of *C. bursa-pastoris* for the Cbp_{Cg} and Cbp_{Co} subgenomes, respectively. The root *N. paniculata* is not shown. ASI, EUR ME, CO, CG, CR indicate Asian, European and Middle Eastern populations of *C. bursa-pastoris, C. orientalis, C. grandiflora*, and *C. rubella*, respectively.
