# Supplementary material for: Parental legacy, demography, and admixture influenced the evolution of the two subgenomes of the tetraploid Capsella bursa-pastoris (Brassicaceae)
Source: PLoS Genet. 2019 Feb 15;15(2):e1007949. doi: 10.1371/journal.pgen.1007949 (PMC6395008; doi:10.1371/journal.pgen.1007949)
Supplement: S2 Fig — ASI, EUR and ME are the three populations of C. bursa-pastoris (Cbp) with Co and Cg indicating two subgenomes. CO, CG, CR are short forms for C. orientalis, C. grandiflora, and C. rubella respectively. (PDF) [file pgen.1007949.s002.pdf]

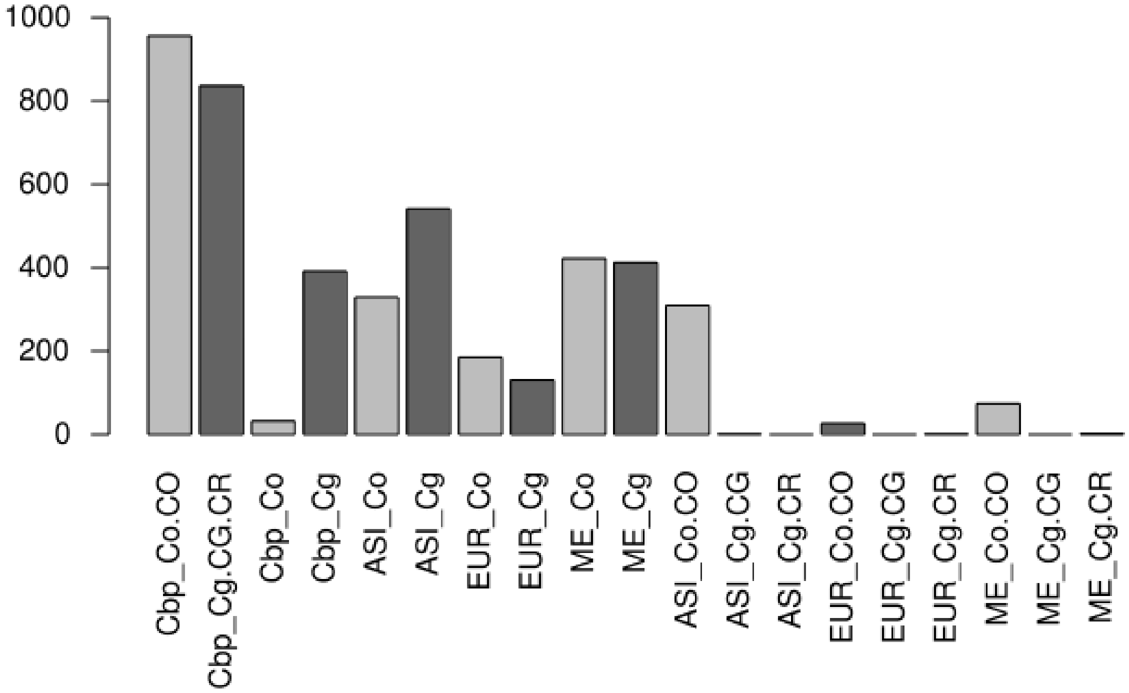

S2 Figure. Frequency of monophyly of different groups. ASI, EUR and ME are the three populations of *C. bursa-pastoris* (Cbp) with Co and Cg indicating two subgenomes. CO, CG, CR are short forms for *C. orientalis, C. grandiflora*, and *C. rubella* respectively.
