# Supplementary material for: Parental legacy, demography, and admixture influenced the evolution of the two subgenomes of the tetraploid Capsella bursa-pastoris (Brassicaceae)
Source: PLoS Genet. 2019 Feb 15;15(2):e1007949. doi: 10.1371/journal.pgen.1007949 (PMC6395008; doi:10.1371/journal.pgen.1007949)
Supplement: S3 Fig — Mean LD was assessed for 100 window bins (each window 1000 SNP wide) within the A) CbpCg and B) CbpCo subgenomes, C) between the CbpCg and CbpCo subgenomes, and D) among SNPs randomly sampled from either subgenome. ASI, EUR and ME are the three populations of C. bursa-pastoris. (PDF) [file pgen.1007949.s003.pdf]

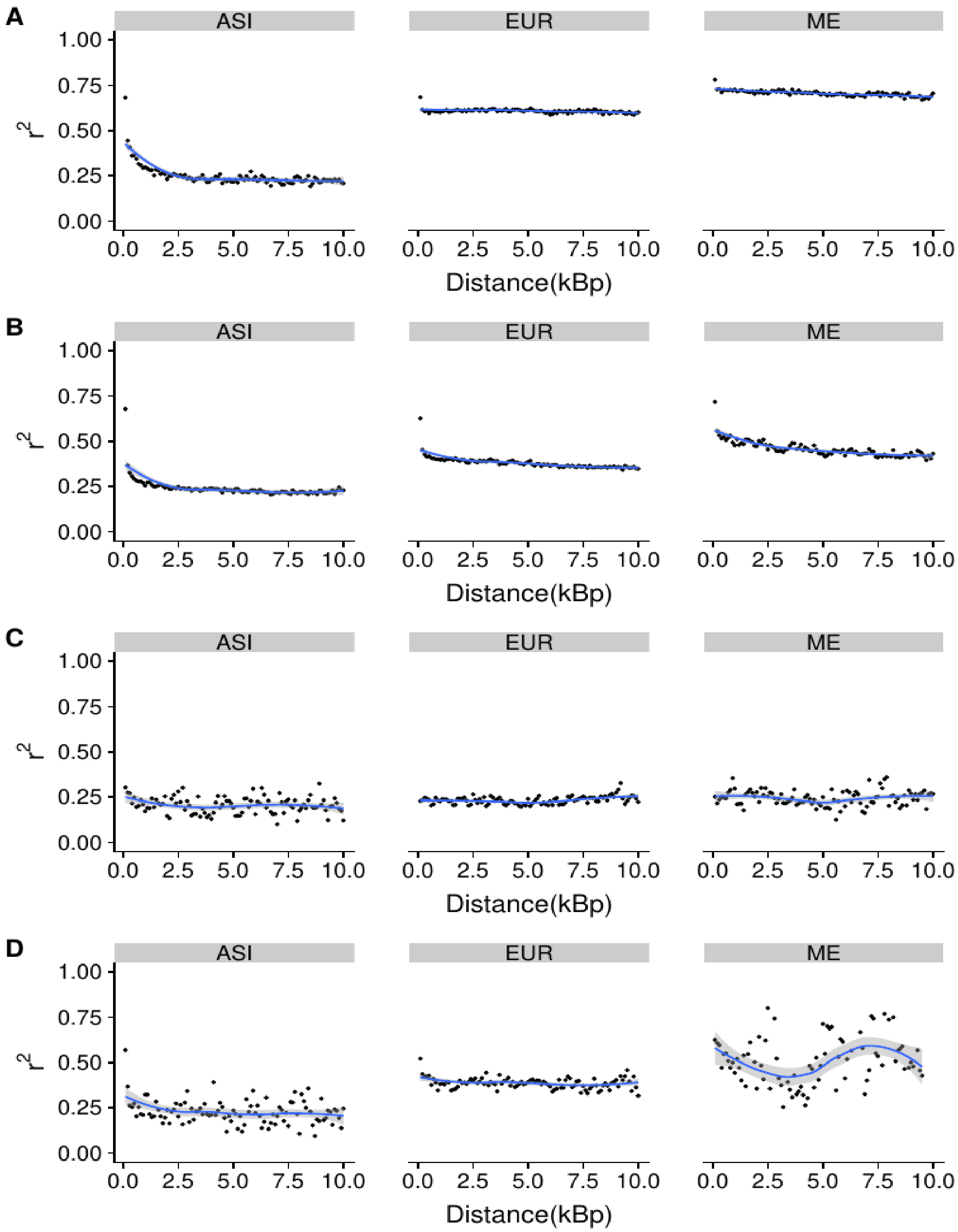

S3 Figure. Comparison of linkage disequilibrium decay within and between subgenomes. Mean LD was assessed for 100 window bins (each window 1000 SNP wide) within the **A**) Cbp_{Cg} and **B**) Cbp_{Co} subgenomes, **C**) between the Cbp_{Cg} and Cbp_{Cg} subgenomes, and **D**) among SNPs randomly sampled from either subgenome. ASI, EUR and ME are the three populations of *C. bursa-pastoris*
