# Supplementary material for: Parental legacy, demography, and admixture influenced the evolution of the two subgenomes of the tetraploid Capsella bursa-pastoris (Brassicaceae)
Source: PLoS Genet. 2019 Feb 15;15(2):e1007949. doi: 10.1371/journal.pgen.1007949 (PMC6395008; doi:10.1371/journal.pgen.1007949)
Supplement: S4 Fig — GATK SNPs include all high-quality unphased SNPs. HapCUT SNPs include all successfully phased GATK SNPs. Overlap with HomeoRoq was possible to access only for positions that were aligned between the C. rubella reference and the C. bursa-pastoris assembly. (PDF) [file pgen.1007949.s004.pdf]

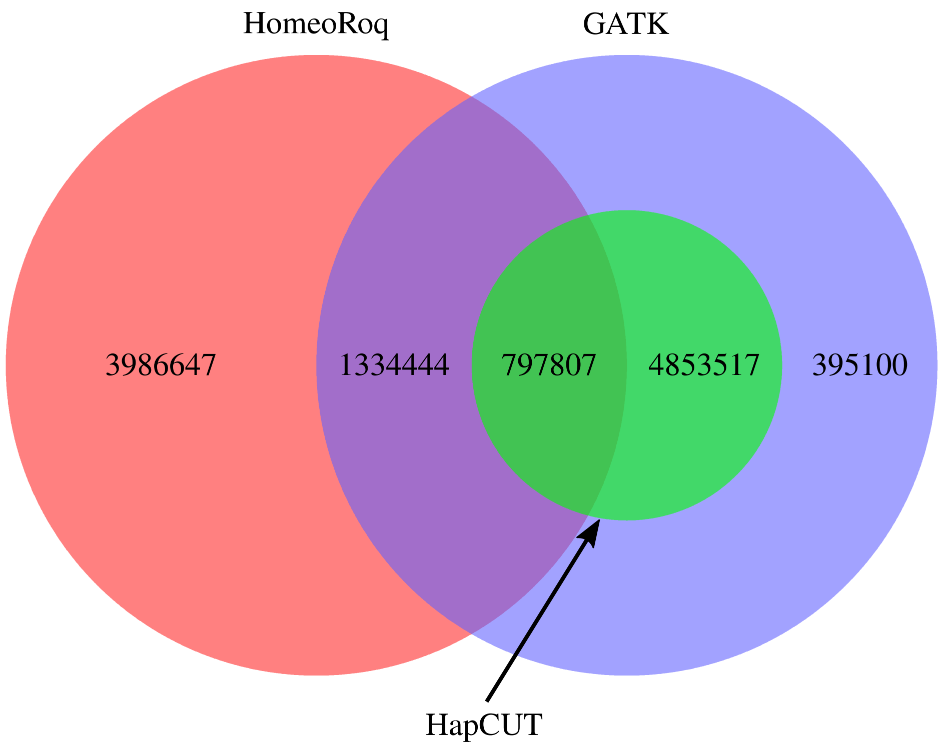

S4 Figure. Overlap in SNPs called with GATK, HomeoRoq, and HapCUT phasing. GATK SNPs include all high-quality unphased SNPs. HapCUT SNPs include all successfully phased GATK SNPs. Overlap with HomeoRoq was possible to access only for positions that were aligned between the *C. rubella* reference and the *C. bursa-pastoris* assembly.
