# Supplementary material for: Parental legacy, demography, and admixture influenced the evolution of the two subgenomes of the tetraploid Capsella bursa-pastoris (Brassicaceae)
Source: PLoS Genet. 2019 Feb 15;15(2):e1007949. doi: 10.1371/journal.pgen.1007949 (PMC6395008; doi:10.1371/journal.pgen.1007949)
Supplement: S7 Fig — The trees were reconstructed with the neighbor-joining algorithm and absolute genetic distance for non-overlapping 1 K windows with minimum 100 complete sites. Trees were tested for mutual monophyly of both the CbpCg and CbpCo subgenomes. There were 132 trees that failed this test. HomeoRoq phased samples have _A and _B in their names to indicate CbpCg and CbpCo subgenomes, respectively, whereas samples phased with HapCUT are marked with _Cg and _Co for the corresponding subgenomes. ASI, EUR ME indicate Asian, European and Middle Eastern populations of C. bursa-pastoris, respectively. (PDF) [file pgen.1007949.s007.pdf]

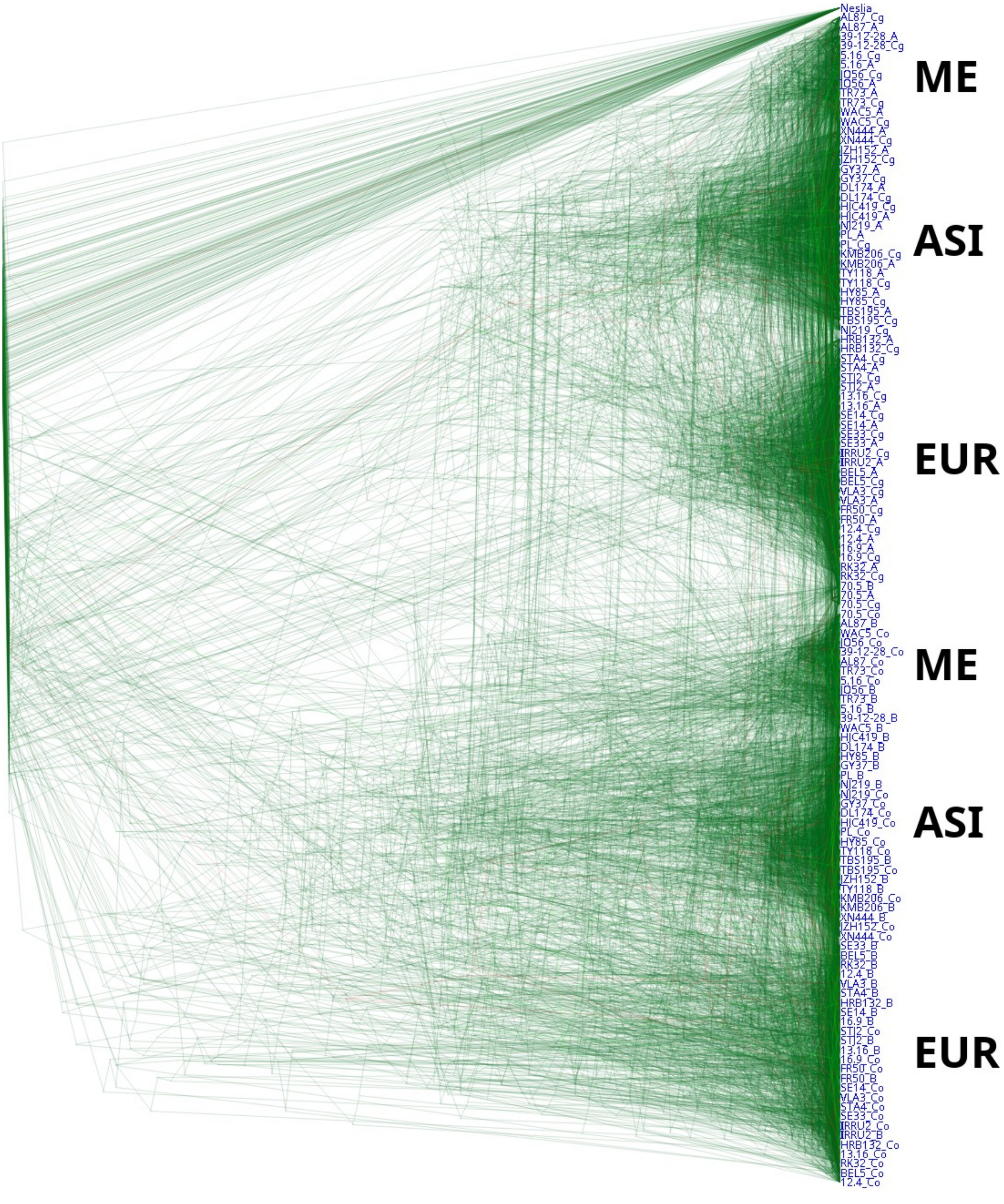

S7 Figure. Phylogenetic trees that failed the subgenomes mutual monophyly test in 1K sliding windows for the alternatively phased datasets. The trees were reconstructed with the neighbor-joining algorithm and absolute genetic distance for non-overlapping 1K windows with minimum 100 complete sites. Trees were tested for mutual monophyly of both the *Cbp*$_{Cg}$ and *Cbp*$_{Co}$ subgenomes. There were 132 trees that failed this test. HomeoRoq phased samples have _A and _B in their names to indicate *Cbp*$_{Cg}$ and *Cbp*$_{Co}$ subgenomes, respectively, whereas samples phased with HapCUT are marked with _Cg and _Co for the corresponding subgenomes. ASI, EUR ME indicate Asian, European and Middle Eastern populations of *C. bursa-pastoris*, respectively.
