# Supplementary material for: Parental legacy, demography, and admixture influenced the evolution of the two subgenomes of the tetraploid Capsella bursa-pastoris (Brassicaceae)
Source: PLoS Genet. 2019 Feb 15;15(2):e1007949. doi: 10.1371/journal.pgen.1007949 (PMC6395008; doi:10.1371/journal.pgen.1007949)
Supplement: S8 Fig — The corresponding subgenomes are marked with Cg and Co. Estimates were assessed for 100 K windows on SNPs data and scaled to whole genome values. (PDF) [file pgen.1007949.s008.pdf]

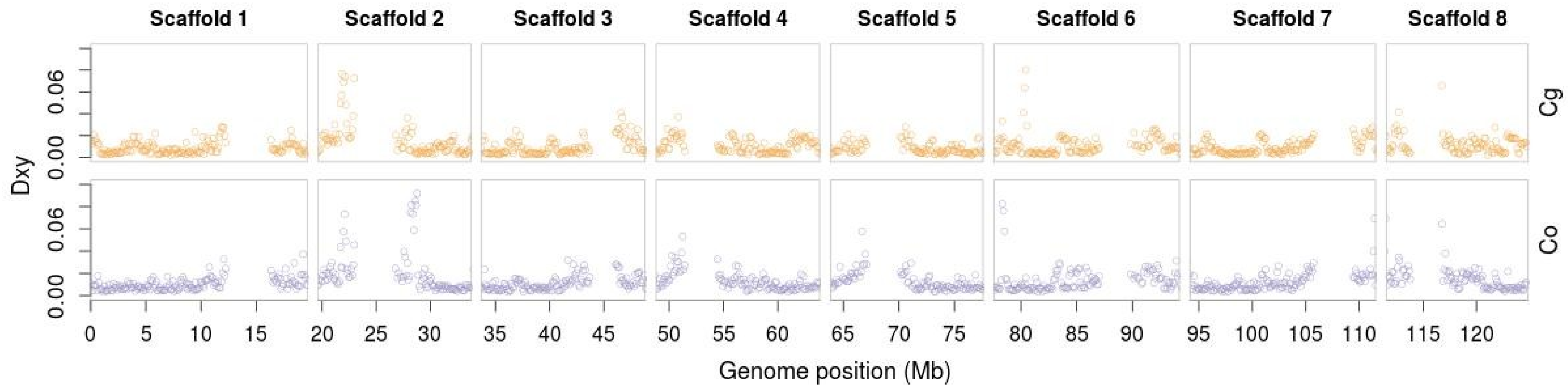

S8 Figure. Genetic distance (Dxy) between the data analyzed in this study and the assembly of *C. bursa-pastoris* by Kasianov et al. (2017). The corresponding subgenomes are marked with Cg and Co. Estimates were assessed for 100K windows on SNPs data and scaled to whole genome values.
