# Supplementary material for: Parental legacy, demography, and admixture influenced the evolution of the two subgenomes of the tetraploid Capsella bursa-pastoris (Brassicaceae)
Source: PLoS Genet. 2019 Feb 15;15(2):e1007949. doi: 10.1371/journal.pgen.1007949 (PMC6395008; doi:10.1371/journal.pgen.1007949)
Supplement: S9 Fig — The tree was reconstructed with the neighbor-joining algorithm and absolute genetic distance for 800 K SNPs. HomeoRoq phased samples have _A and _B in their names to indicate CbpCg and CbpCo subgenomes, respectively, whereas samples phased with HapCUT are marked with _Cg and _Co for the corresponding subgenomes. The reference sequences of the assembled C. bursa-pastoris are named with TARG. CO, CG, ASI, EUR, ME indicate C. orientalis, and C. grandiflora, and Asian, European and Middle Eastern populations of C. bursa-pastoris, respectively. (PDF) [file pgen.1007949.s009.pdf]

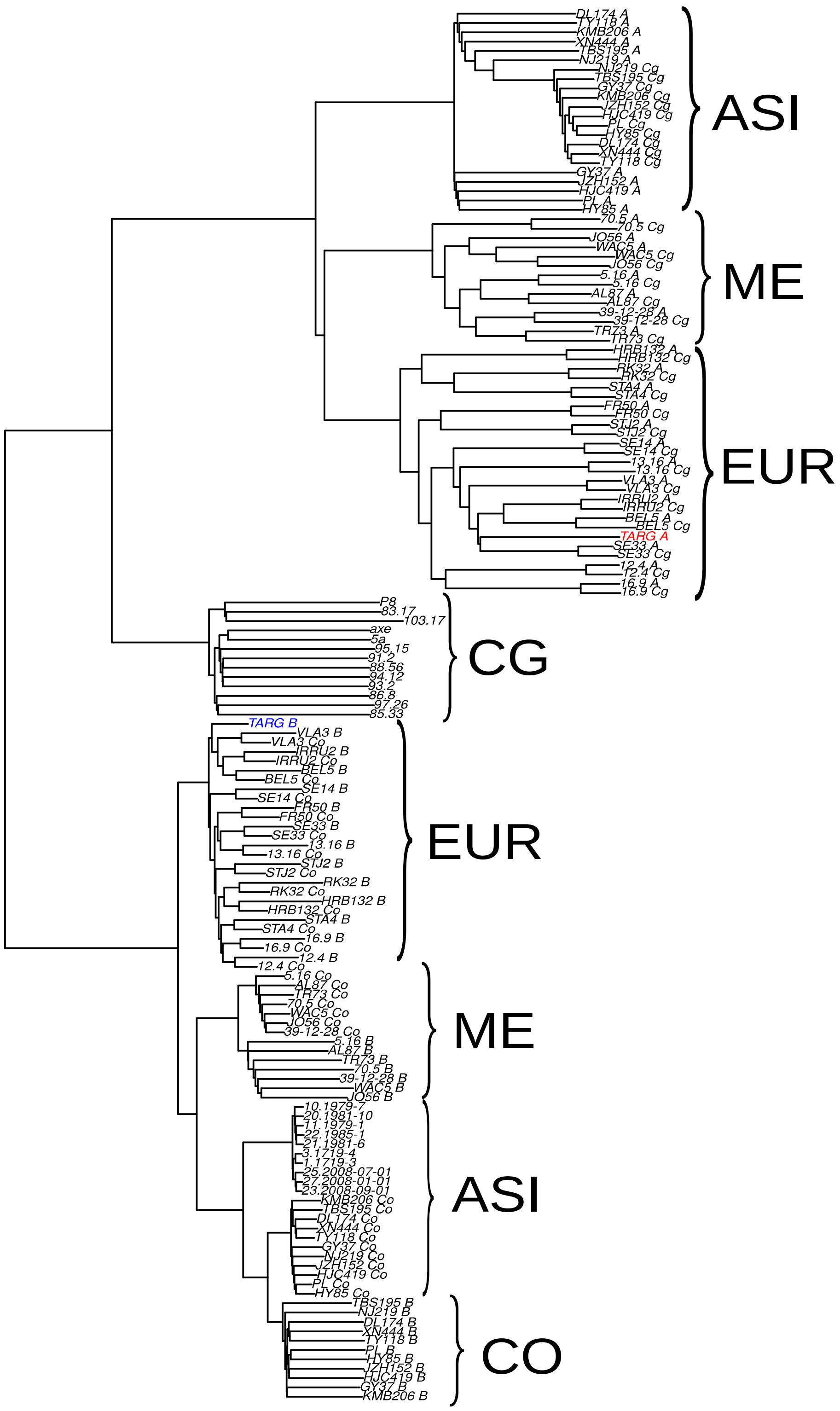

S9 Figure. Phylogenetic relationship of the alternatively phased datasets with parental species. The tree was reconstructed with the neighbor-joining algorithm and absolute genetic distance for 800K SNPs. HomeoRoq phased samples have _A and _B in their names to indicate *Cbp*$_{Cg}$ and *Cbp*$_{Co}$ subgenomes, respectively, whereas samples phased with HapCUT are marked with _Cg and _Co for the corresponding subgenomes. The reference sequences of the assembled *C.bursa-pastoris* are named with *TARG.* CO, CG, ASI, EUR, ME indicate *C. orientalis*, and *C. grandiflora*, and Asian, European and Middle Eastern populations of *C. bursa-pastoris*, respectively.
