# Supplementary material for: Parental legacy, demography, and admixture influenced the evolution of the two subgenomes of the tetraploid Capsella bursa-pastoris (Brassicaceae)
Source: PLoS Genet. 2019 Feb 15;15(2):e1007949. doi: 10.1371/journal.pgen.1007949 (PMC6395008; doi:10.1371/journal.pgen.1007949)
Supplement: S10 Fig — The distributions of π were estimated from 797,806 SNPs using 100 Kb sliding windows and scaled to whole genome scale. Co and Cg indicate C. orientalis and C. grandiflora/rubella descendant subgenomes, respectively. ASI, EUR, and ME correspond to Asian, European and Middle Eastern populations of C. bursa-pastoris, respectively. (PDF) [file pgen.1007949.s010.pdf]

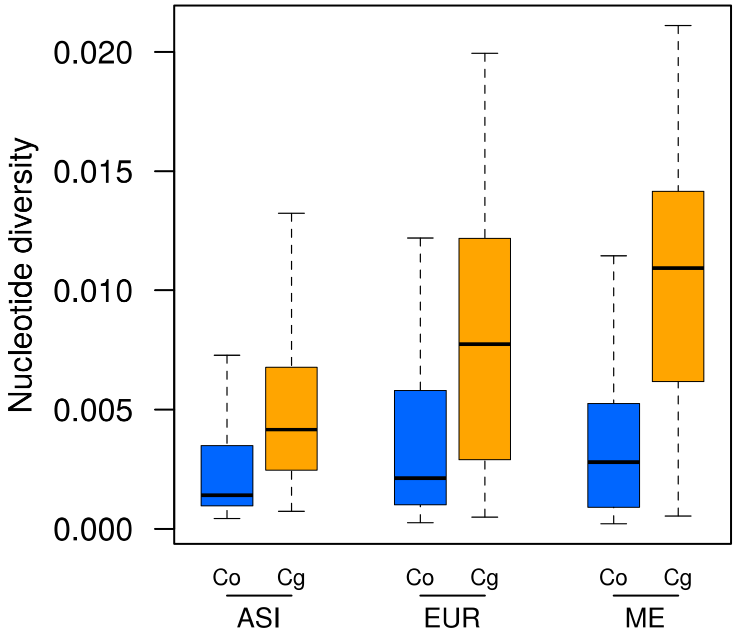

S10 Figure. Variation in nucleotide diversity (π) between subgenomes of *C. bursa-pastoris* in HomeoRoq phased data. The distributions of π were estimated from 797,806 SNPs using 100 Kb sliding windows and scaled to whole genome scale. *Co* and *Cg* indicate *C. orientalis* and *C. grandiflora/rubella* descendant subgenomes, respectively. ASI, EUR, and ME correspond to Asian, European and Middle Eastern populations of *C. bursa-pastoris*, respectively.
