# Supplementary material for: Parental legacy, demography, and admixture influenced the evolution of the two subgenomes of the tetraploid Capsella bursa-pastoris (Brassicaceae)
Source: PLoS Genet. 2019 Feb 15;15(2):e1007949. doi: 10.1371/journal.pgen.1007949 (PMC6395008; doi:10.1371/journal.pgen.1007949)
Supplement: S11 Fig — Population sizes were inferred using whole-genome sequences from six randomly chosen haplotypes per population with PSMC and all haplotypes with SMC++. The estimates for PSMC and SMC++ are designated with different line thickness. Co and Cg specify subgenomes of C. bursa-pastoris and corresponding parental species in the CO & CG plot. ASI, EUR, ME, CO & CG indicate Asian, European and Middle Eastern populations of C. bursa-pastoris, and C. orientalis and C. grandiflora, respectively. The axes are in log scale and the most recent time, where PSMC is less reliable is excluded. (PDF) [file pgen.1007949.s011.pdf]

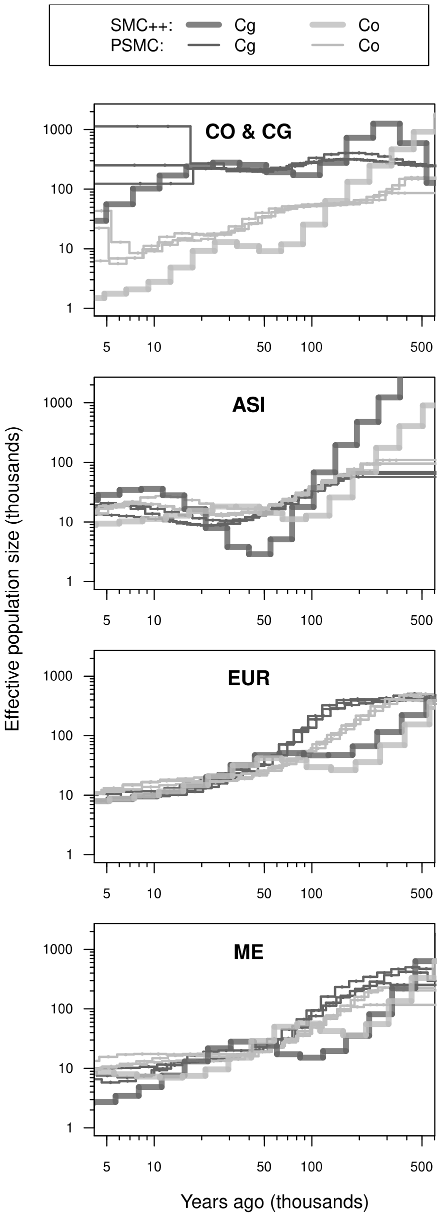

S11 Figure. Population size histories of *C. bursa-pastoris* and its parental species estimated with PSMC and SMC++. Population sizes were inferred using whole-genome sequences from six randomly chosen haplotypes per population with PSMC and all haplotypes with SMC++. The estimates for PSMC and SMC++ are designated with different line thickness. Co and Cg specify subgenomes of *C. bursa-pastoris* and corresponding parental species in the CO & CG plot. ASI, EUR, ME, CO & CG indicate Asian, European and Middle Eastern populations of *C. bursa-pastoris*, and *C. orientalis* and *C. grandiflora*, respectively. The axes are in log scale and the most recent time, where PSMC is less reliable is excluded.

The results of SMC++ were largely similar to the results of PSMC. The only difference was that SMC++ showed larger scale differences between the N_e trajectories of the two subgenomes in the ASI and ME populations than PSMC. SMC++ also showed more fluctuation in the N_e of both *C. orientalis* and *C. grandiflora*, but the trajectories were similar to those obtained with PSMC. The difference in N_e fluctuation scale was supported by our simulations.
