# Supplementary material for: Parental legacy, demography, and admixture influenced the evolution of the two subgenomes of the tetraploid Capsella bursa-pastoris (Brassicaceae)
Source: PLoS Genet. 2019 Feb 15;15(2):e1007949. doi: 10.1371/journal.pgen.1007949 (PMC6395008; doi:10.1371/journal.pgen.1007949)
Supplement: S12 Fig — A. Fifteen possible rooted topologies for the three groups of C. bursa-pastoris in one subgenome and corresponding parental species. The topologies are grouped into five main groups. Co and Cg indicate the two subgenomes. ASI, EUR ME, CO, CG, N indicate Asian, European and Middle Eastern populations of C. bursa-pastoris, C. orientalis, C. grandiflora, and N. paniculata, respectively. B. Topology weightings for 100 SNP windows plotted along 8 main scaffolds with loess smoothing (span = 1Mb). The tentative centromeric regions are shaded. C. Average weighting for the five main topology groups. (PDF) [file pgen.1007949.s012.pdf]

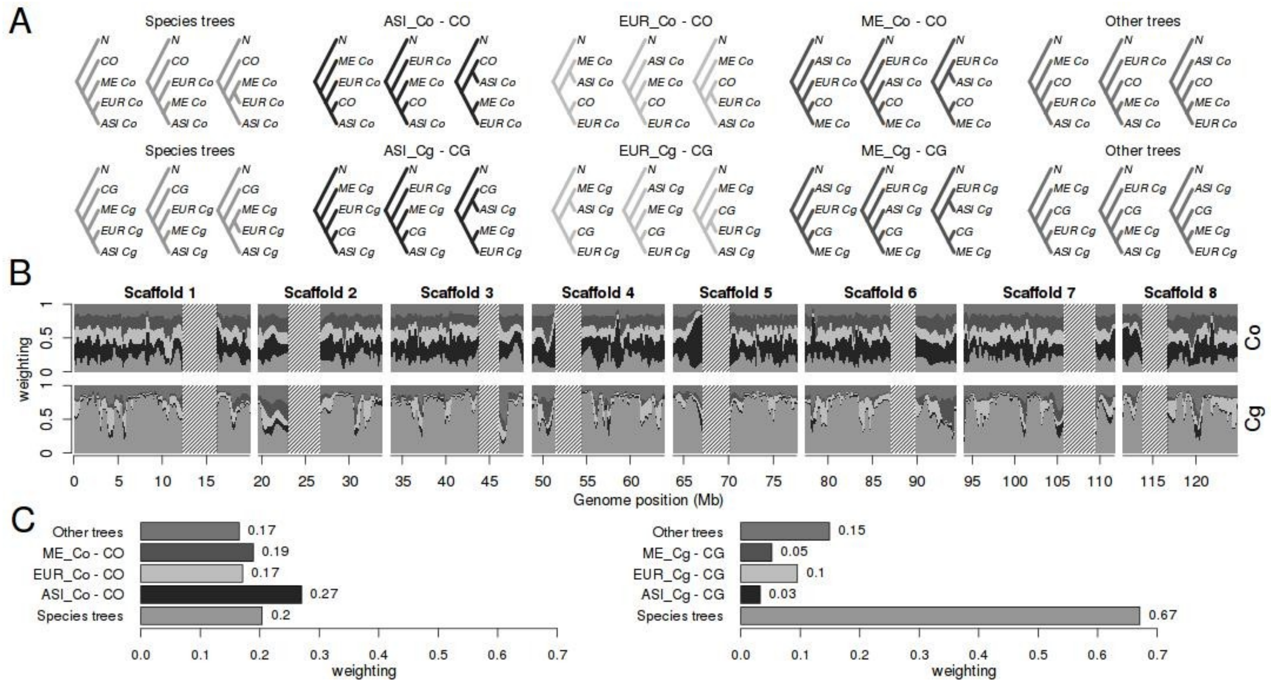

S12 Figure. Topology weighting of the three populations of *C. bursa-pastoris, C. orientalis,* and *C. grandiflora*. A. Fifteen possible rooted topologies for the three groups of *C. bursa-pastoris* in one subgenome and corresponding parental species. The topologies are grouped into five main groups. Co and Cg indicate the two subgenomes. ASI, EUR ME, CO, CG, N indicate Asian, European and Middle Eastern populations of *C.bursa-pastoris, C. orientalis*, *C. grandiflora*, and *N. paniculata*, respectively. **B**.Topology weightings for 100 SNP windows plotted along 8 main scaffolds with loess smoothing (span = 1Mb). The tentative centromeric regions are shaded. **C.** Average weighting for the five main topology groups.
