# Supplementary material for: Parental legacy, demography, and admixture influenced the evolution of the two subgenomes of the tetraploid Capsella bursa-pastoris (Brassicaceae)
Source: PLoS Genet. 2019 Feb 15;15(2):e1007949. doi: 10.1371/journal.pgen.1007949 (PMC6395008; doi:10.1371/journal.pgen.1007949)
Supplement: S13 Fig — A. Estimated admixture between C. rubella (dark red regions) and the European Cg subgenome, relative to the Middle Eastern Cg subgenome (green regions). B. Estimated admixture between C. orientalis (orange regions) and the European Co subgenome, relative to the Middle Eastern Co subgenome (green regions). The limits of centromeric regions are indicated by vertical dashed lines. (PDF) [file pgen.1007949.s013.pdf]

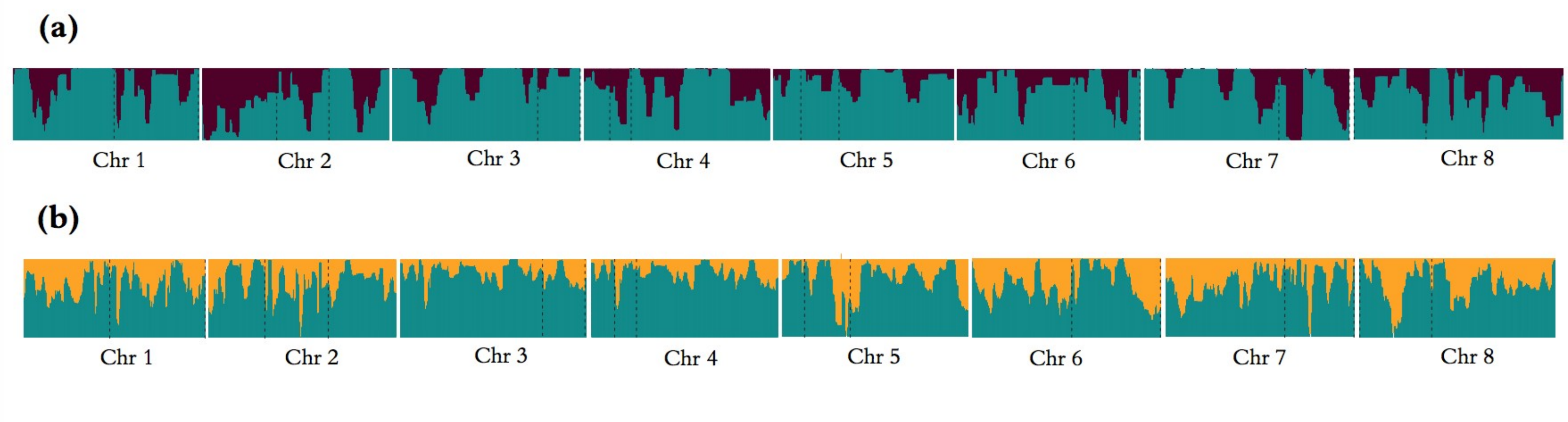

S13 Figure. Bar plots of HAPMIX admixture probabilities for major scaffolds (chromosomes) of *C. bursa-pastoris*. (**a**) Estimated admixture between *C. rubella* (dark red regions) and the European Cbp_{Cg} subgenome, relative to the Middle Eastern Cbp_{Cg} subgenome (green regions). Estimated admixture between (**b**) *C. orientalis* (orange regions) and the European Cbp_{Co} subgenome, relative to the Middle Eastern Cbp_{Co} subgenome (green regions). The limits of centromeric regions are indicated by vertical dashed lines.
