# Supplementary material for: Parental legacy, demography, and admixture influenced the evolution of the two subgenomes of the tetraploid Capsella bursa-pastoris (Brassicaceae)
Source: PLoS Genet. 2019 Feb 15;15(2):e1007949. doi: 10.1371/journal.pgen.1007949 (PMC6395008; doi:10.1371/journal.pgen.1007949)
Supplement: S14 Fig — A. Nucleotide diversity in 0-fold (π0). B. Nucleotide diversity in 4-fold sites (π4). C. The ratio between π0 and π4. ASI, EUR and ME are the three differentiated populations of C. bursa-pastoris. Co and Cg indicate corresponding subgenomes. CO and CG are short forms for C. orientalis and C. grandiflora, respectively. (PDF) [file pgen.1007949.s014.pdf]

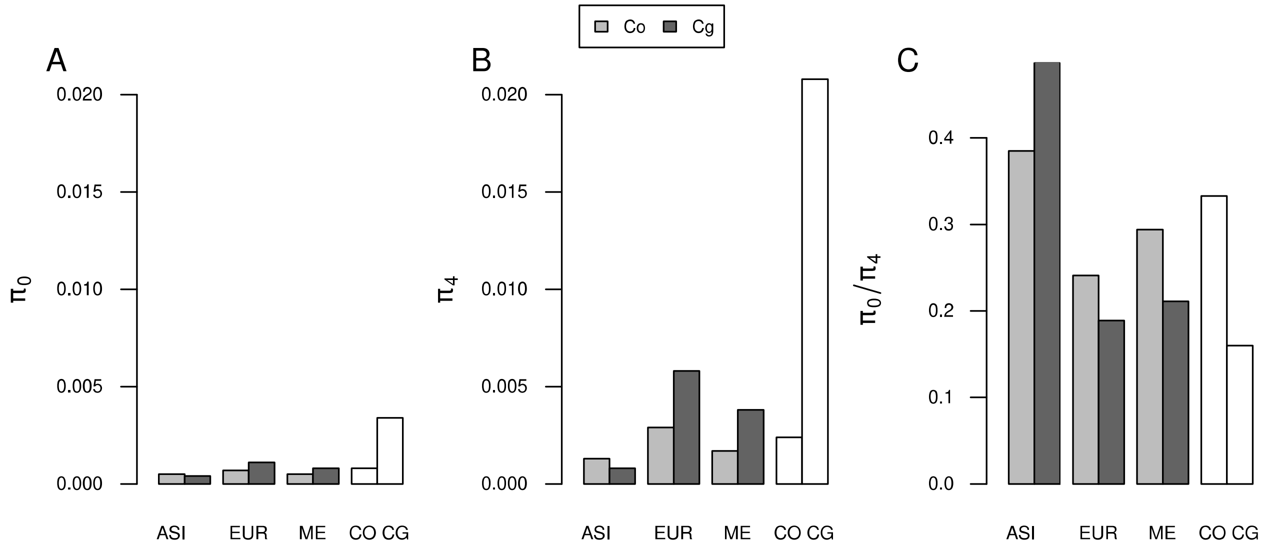

S14 Figure. Nucleotide diversity (π) in the coding part of the genome. A. Nucleotide diversity in 0-fold (π_0). **B.** Nucleotide diversity in 4-fold sites (π_4). **C.** The ratio between π_0 and π_4. ASI, EUR and ME are the three differentiated populations of *C. bursa-pastoris*. Co and Cg indicate corresponding subgenomes. *CO* and *CG* are short forms for *C. orientalis* and *C. grandiflora*, respectively.
