# Supplementary material for: Parental legacy, demography, and admixture influenced the evolution of the two subgenomes of the tetraploid Capsella bursa-pastoris (Brassicaceae)
Source: PLoS Genet. 2019 Feb 15;15(2):e1007949. doi: 10.1371/journal.pgen.1007949 (PMC6395008; doi:10.1371/journal.pgen.1007949)
Supplement: S15 Fig — A. The analysis was performed on the fragments that were 100% consistent in their phasing state with parental species. B. For ease of comparison, we also duplicate Fig 6 that shows the results for the data phased with 90% consistency with the parental species. The 0.01 significance level defined with data simulated under a standard neutral model is depicted with dashed lines. Pericentromeric regions are removed. Only eight major scaffolds (chromosomes) are shown. Selective sweeps are detected with the composite likelihood ratio statistics (CLR) along the CbpCo and CbpCg subgenomes in Asian (ASI), European (EUR) and Middle Eastern (ME) populations. (PDF) [file pgen.1007949.s015.pdf]

A

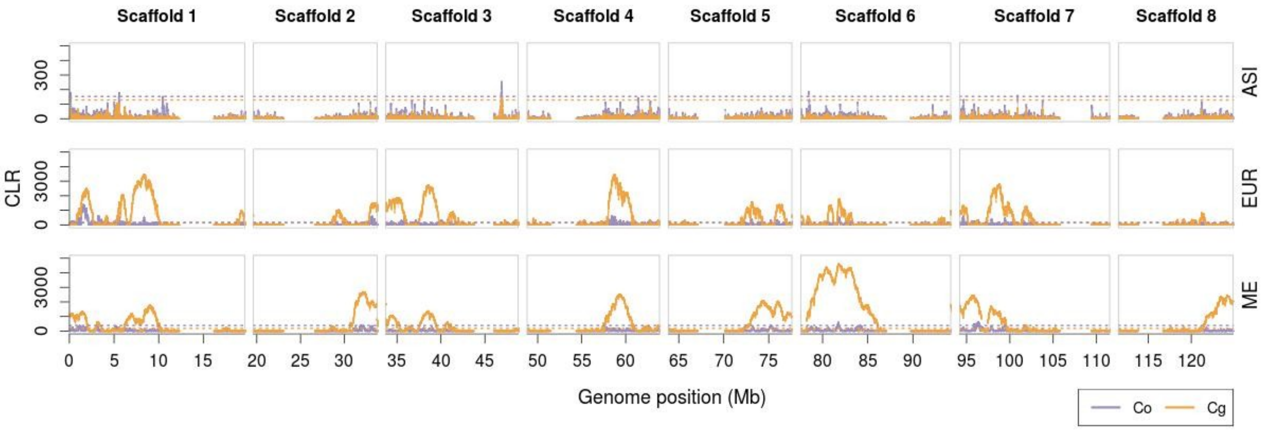

B

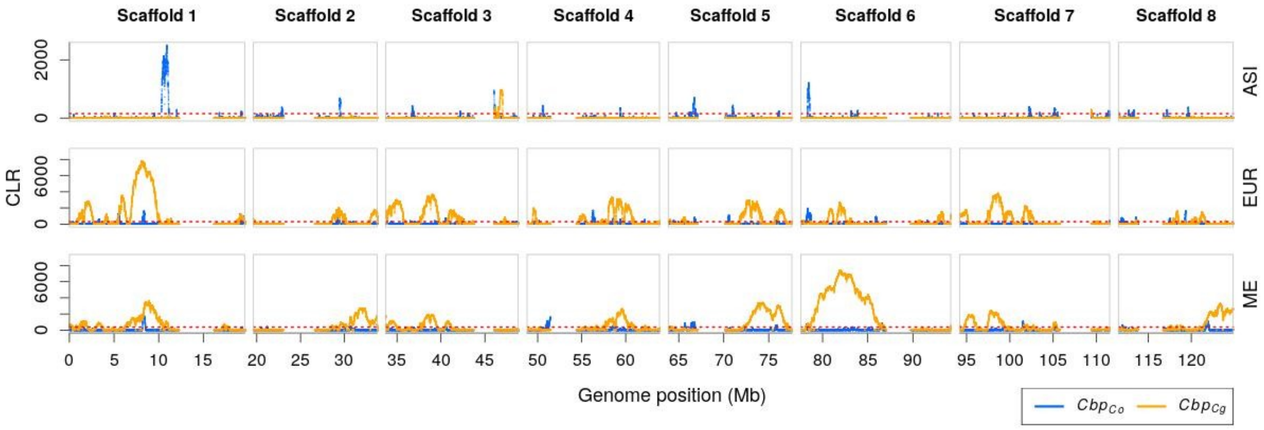

S15 Figure. Selective sweep differences between populations of *C. bursa-pastoris* in the most stringently filtered data. A. The analysis was performed on the fragments that were 100% consistent in their phasing state with parental species. **B.** For ease of comparison, we also duplicate Fig. 6 that shows the results for the data phased with 90% consistency with the parental species. The 0.01 significance level defined with data simulated under a standard neutral model is depicted with dashed lines. Pericentromeric regions are removed. Only eight major scaffolds (chromosomes) are shown. Selective sweeps are detected with the composite likelihood ratio statistics (CLR) along the Cbp_{Co} and Cbp_{Cg} subgenomes in Asian (ASI), European (EUR) and Middle Eastern (ME) populations.
