# Supplementary material for: Parental legacy, demography, and admixture influenced the evolution of the two subgenomes of the tetraploid Capsella bursa-pastoris (Brassicaceae)
Source: PLoS Genet. 2019 Feb 15;15(2):e1007949. doi: 10.1371/journal.pgen.1007949 (PMC6395008; doi:10.1371/journal.pgen.1007949)
Supplement: S16 Fig — The vertical lines indicate the used cut-offs. DP—combined depth per SNP across samples. QD—variant confidence standardized by depth. MQ—Mapping quality of a SNP. FS—strand bias in support for REF vs ALT allele calls. SOR—sequencing bias in which one DNA strand is favored over the other. MQRankSum—rank sum test for mapping qualities of REF vs. ALT reads. ReadPosRankSum—indicates if all the reads supporting a SNP call tend to be near the end of a read. Indels were not used in this study. (PDF) [file pgen.1007949.s016.pdf]

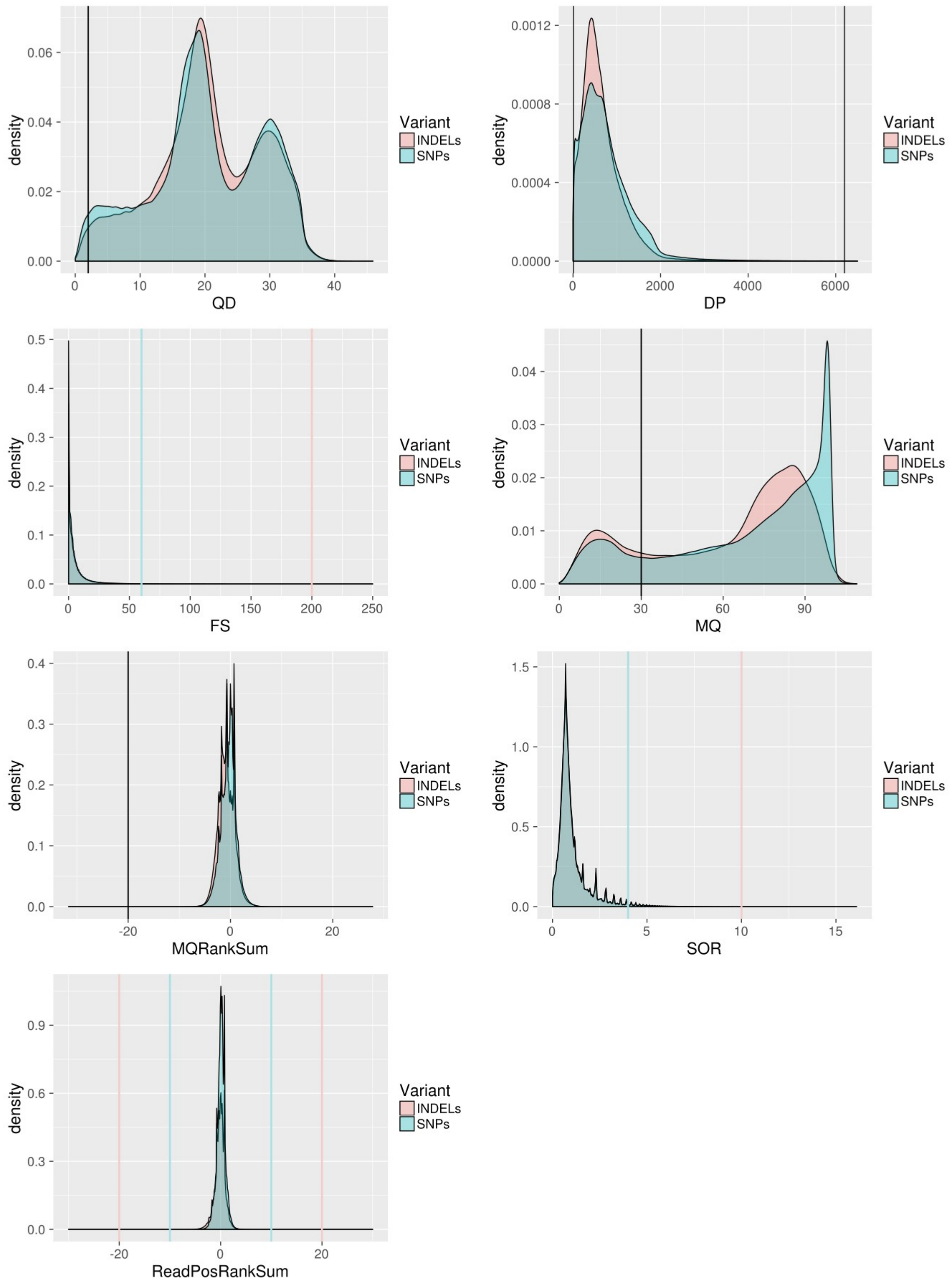

S16 Figure. Distribution of GATK annotation scores. The vertical lines indicate the used cut-offs. DP - combined depth per SNP across samples. QD - variant confidence standardized by depth. MQ - Mapping quality of a SNP. FS - strand bias in support for REF vs ALT allele calls. SOR - sequencing bias in which one DNA strand is favored over the other. MQRankSum - rank sum test for mapping qualities of REF vs. ALT reads. ReadPosRankSum – indicates if all the reads supporting a SNP call tend to be near the end of a read. Indels were not used in this study.
