# Supplementary material for: Parental legacy, demography, and admixture influenced the evolution of the two subgenomes of the tetraploid Capsella bursa-pastoris (Brassicaceae)
Source: PLoS Genet. 2019 Feb 15;15(2):e1007949. doi: 10.1371/journal.pgen.1007949 (PMC6395008; doi:10.1371/journal.pgen.1007949)
Supplement: S17 Fig — We were able to phaseheterozygous sites that are fixed between the CbpCo and CbpCg subgenomes. The level of diversity within each subgenome is extremely low, so such variation as at the position 7 was ignored. HapCUT was able to phase regions with enough SNPs density to get overlapping reads with polymorphic sites (orange reads). However, there were gaps between these phased haplotype blocks. To assemble these blocks, we compared them to fixed differences in parental species, and if there were more than 10% discrepancy sites, we considered such block as chimeric and replaced it with Ns. An example of a chimeric block is the block between positions 3 and 4. This filtering resulted in lowering the number of polymorphic sites in the data. To compensate for this, we also randomly introduced missing data in non-polymorphic regions such as in the regions between position 4 and 5. This allowed us to maintain the same level of heterozygosity in the phased data as in the unphased one. (PDF) [file pgen.1007949.s017.pdf]

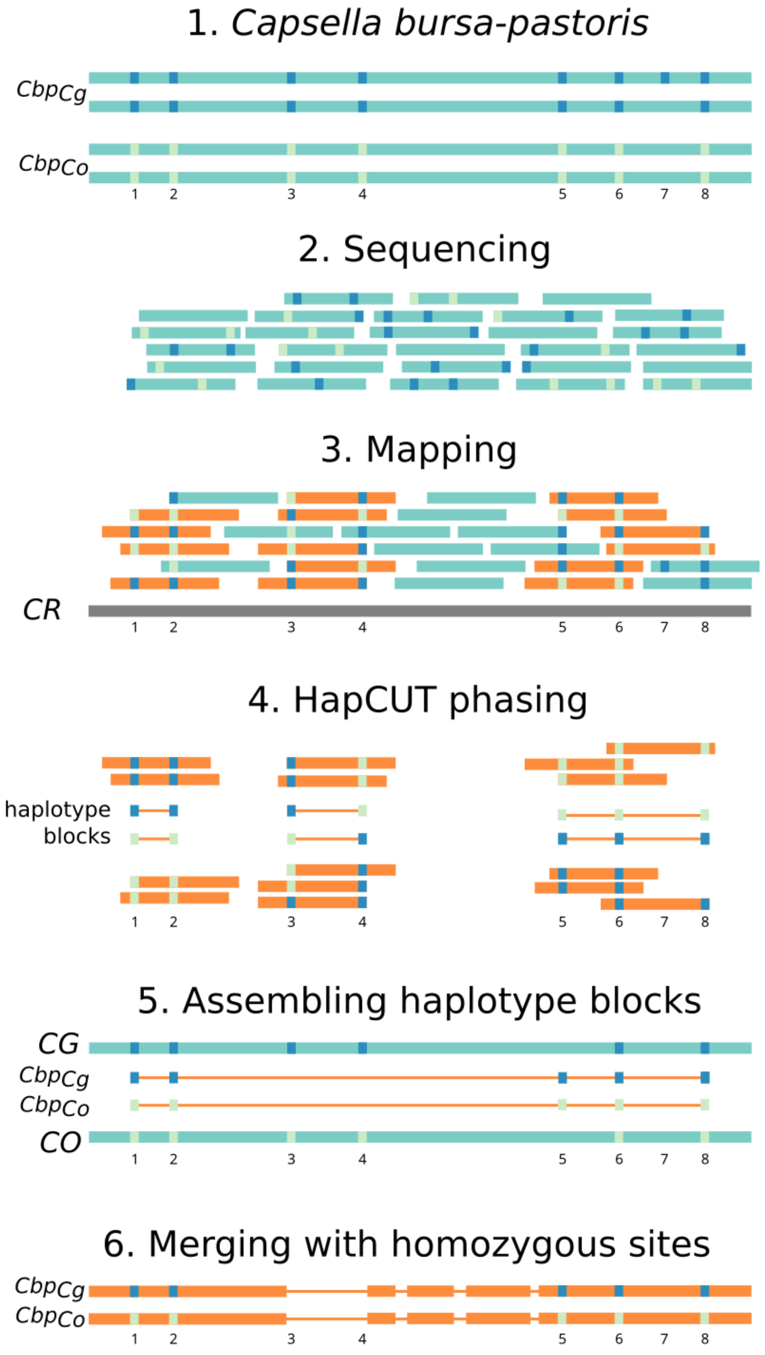

S17 Figure. Phasing scheme used to phase genomic data of *C. bursa-pastoris* in this study. We were able to phase heterozygous sites that are fixed between the Cbp_{Co} and Cbp_{Cg} subgenomes. The level of diversity within each subgenome is extremely low, so such variation as at the position 7 was ignored. HapCUT was able to phase regions with enough SNPs density to get overlapping reads with polymorphic sites (orange reads). However, there were gaps between these phased haplotype blocks. To assemble these blocks, we compared them to fixed differences in parental species, and if there were more than 10% discrepancy sites, we considered such block as chimeric and replaced it with Ns. An example of a chimeric block is the block between positions 3 and 4. This filtering resulted in lowering the number of polymorphic sites in the data. To compensate for this, we also randomly introduced missing data in non-polymorphic regions such as in the regions between position 4 and 5. This allowed us to maintain the same level of heterozygosity in the phased data as in the unphased one.
