# Supplementary material for: Parental legacy, demography, and admixture influenced the evolution of the two subgenomes of the tetraploid Capsella bursa-pastoris (Brassicaceae)
Source: PLoS Genet. 2019 Feb 15;15(2):e1007949. doi: 10.1371/journal.pgen.1007949 (PMC6395008; doi:10.1371/journal.pgen.1007949)
Supplement: S18 Fig — The blocks in the shaded area were considered chimeric and were discarded from the data. All samples had a rather similar distribution as shown in this figure. “Same” means that the alleles were phased in accord with the order Co-Cg; “reverse” indicated the opposite pattern Cg-Co. Thus, 1.0 indicates that all sites are phased as Co-Cg, and 0.0 means that all sites are in the reverse state Cg-Co. 0.5 means that half of alleles are phased as Co-Cg and the other half as Cg-Co. (PDF) [file pgen.1007949.s018.pdf]

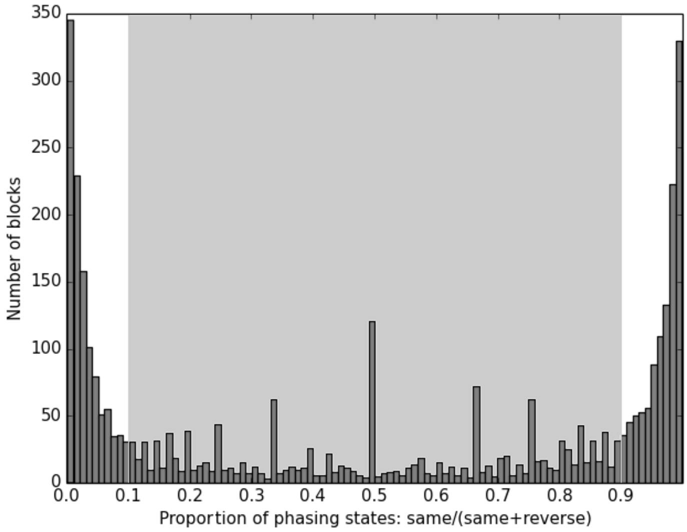

S18 Figure. Example of the distribution of phasing states of haplotype blocks emitted by HapCUT. The blocks in the shaded area were considered chimeric and were discarded from the data. All samples had a rather similar distribution as shown in this figure. “Same” means that the alleles were phased in accord with the order Co-Cg; “reverse” indicated the opposite pattern Cg-Co. Thus, 1.0 indicates that all sites are phased as Co-Cg, and 0.0 means that all sites are in the reverse state Cg-Co. 0.5 means that half of alleles are phased as Co-Cg and the other half as Cg-Co.
