# Supplementary material for: Parental legacy, demography, and admixture influenced the evolution of the two subgenomes of the tetraploid Capsella bursa-pastoris (Brassicaceae)
Source: PLoS Genet. 2019 Feb 15;15(2):e1007949. doi: 10.1371/journal.pgen.1007949 (PMC6395008; doi:10.1371/journal.pgen.1007949)
Supplement: S19 Fig — Mean and median of the fragment length distribution in each sample are shown in absolute numbers. The introduced missing data (Ns) is presented relative to the total number of heterozygous sites that were used for phasing. ASI, EUR and ME accessions are colored by green, red, and blue, respectively. (PDF) [file pgen.1007949.s019.pdf]

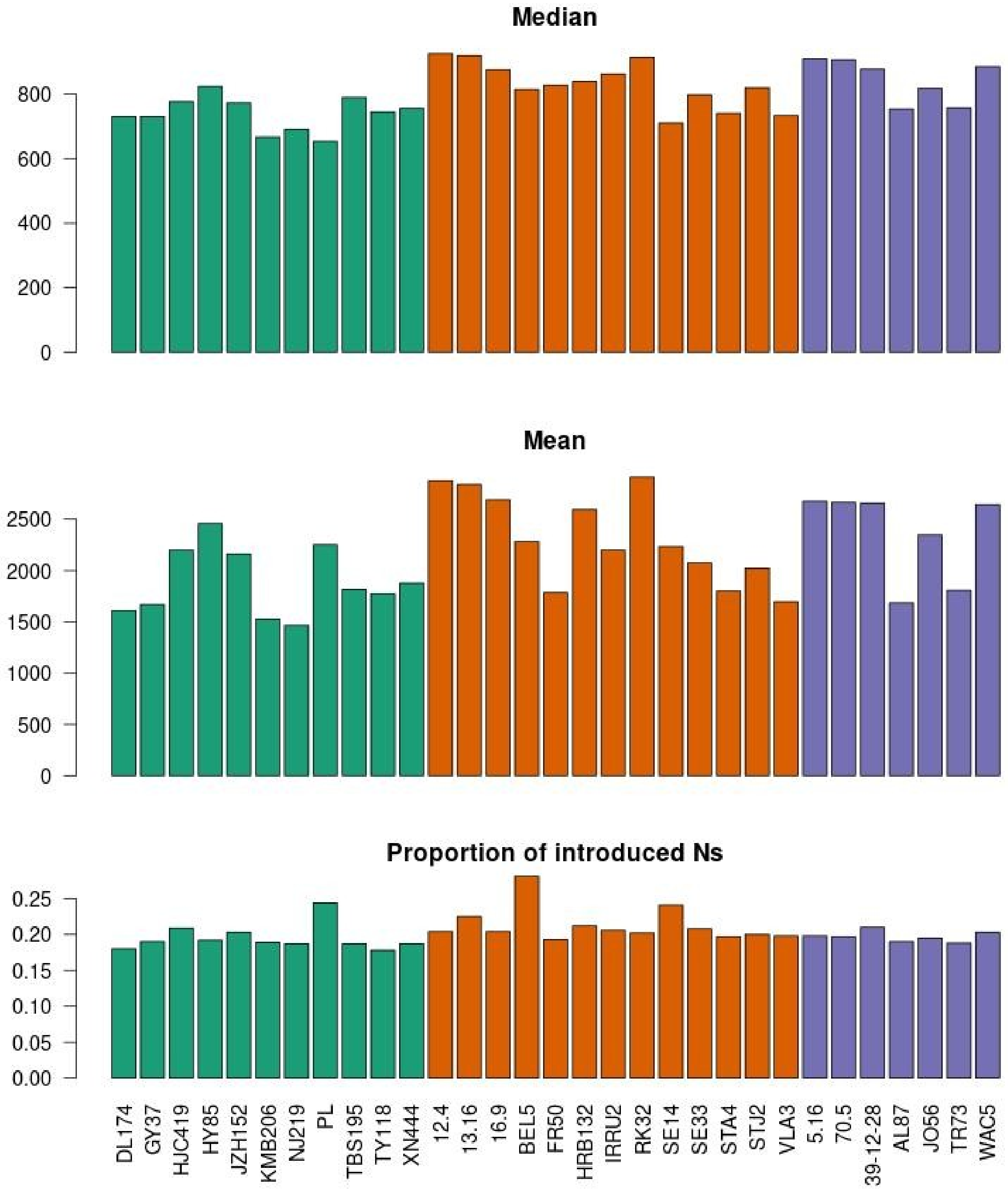

S19 Figure. Variation in the length of phased fragments and proportion of unsuccessfully phased positions across samples. Mean and median of the fragment length distribution in each sample are shown in absolute numbers. The introduced missing data (Ns) is presented relative to the total number of heterozygous sites that were used for phasing. ASI, EUR and ME accessions are colored by green, red, and blue, respectively.
