# Supplementary material for: Parental legacy, demography, and admixture influenced the evolution of the two subgenomes of the tetraploid Capsella bursa-pastoris (Brassicaceae)
Source: PLoS Genet. 2019 Feb 15;15(2):e1007949. doi: 10.1371/journal.pgen.1007949 (PMC6395008; doi:10.1371/journal.pgen.1007949)
Supplement: S20 Fig — All values have been estimated with sliding windows of 100 K genomic positions on phased data. Heterozygosity is shown in relative numbers, while the coverage is in absolute numbers. The regions without the data mostly correspond to pericentromeric regions that were excluded from the data. (PDF) [file pgen.1007949.s020.pdf]

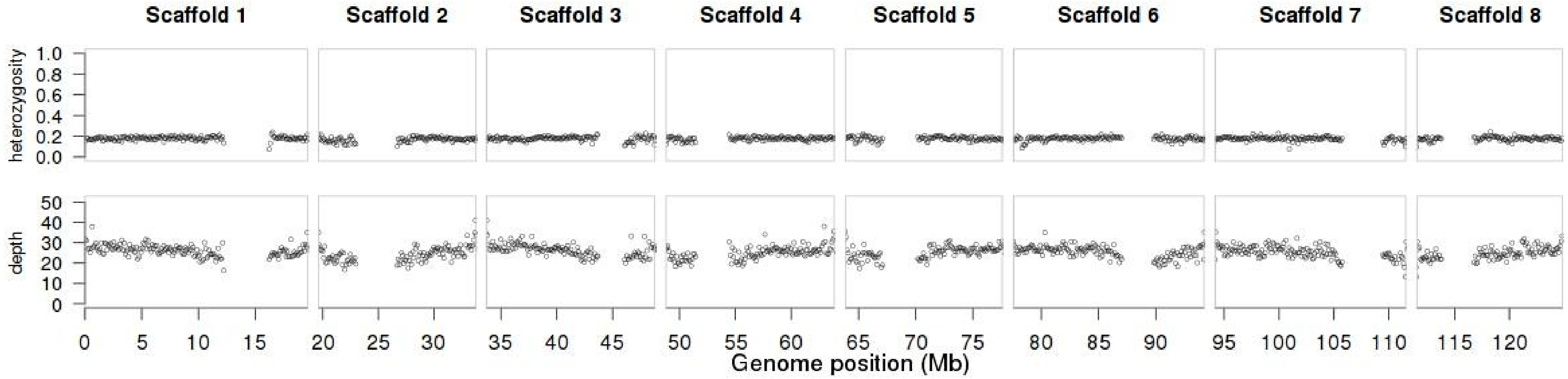

S20 Figure. Verification of non-biased genotyping of both subgenomes of *C. bursa-pastoris* with the distribution of heterozygosity and coverage. All values have been estimated with sliding windows of 100K genomic positions on phased data. Heterozygosity is shown in relative numbers, while the coverage is in absolute numbers. The regions without the data mostly correspond to pericentromeric regions that were excluded from the data.
