# Supplementary material for: Parental legacy, demography, and admixture influenced the evolution of the two subgenomes of the tetraploid Capsella bursa-pastoris (Brassicaceae)
Source: PLoS Genet. 2019 Feb 15;15(2):e1007949. doi: 10.1371/journal.pgen.1007949 (PMC6395008; doi:10.1371/journal.pgen.1007949)
Supplement: S21 Fig — All values have been estimated with sliding windows of 100 K genomic positions on the final dataset that was used in the analyses. ASI, EUR and ME are the three populations of C. bursa-pastoris. CO and CG are short forms for C. orientalis and C. grandiflora, respectively. The regions without the data mostly correspond to pericentromeric regions that were excluded from the data. (PDF) [file pgen.1007949.s021.pdf]

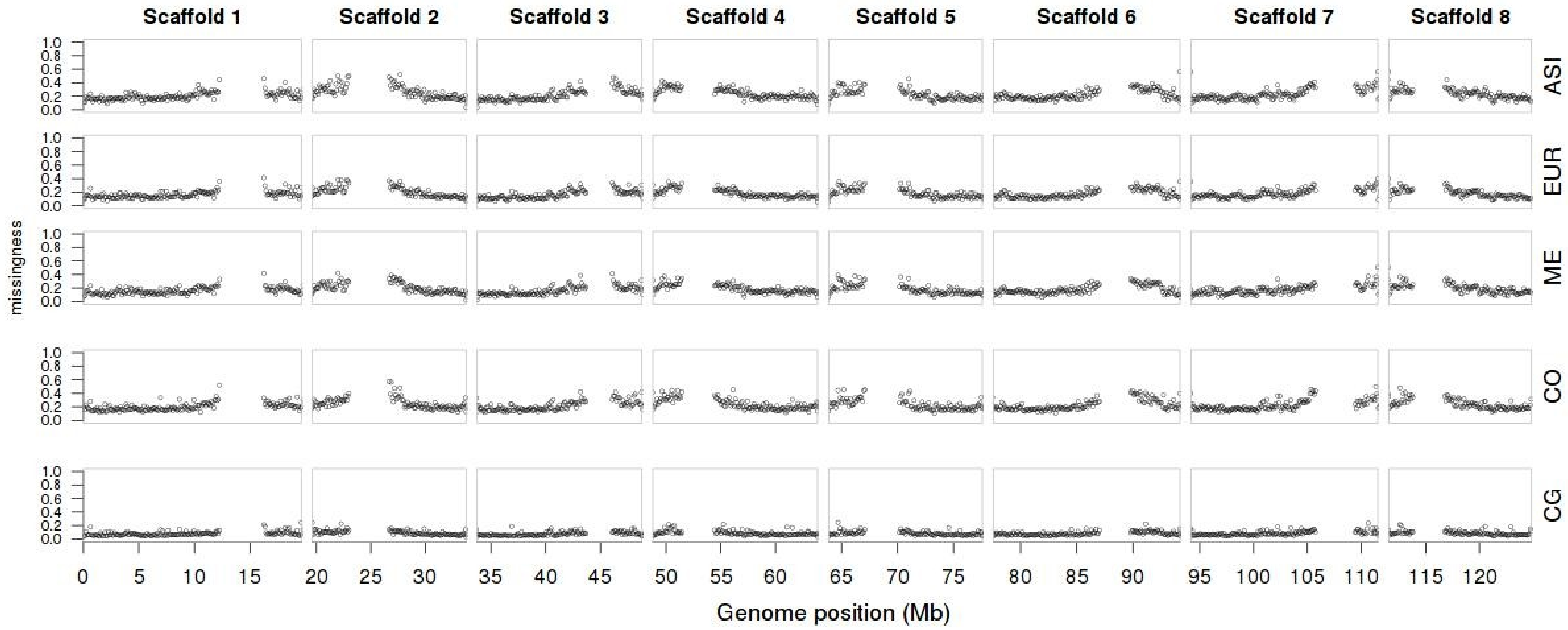


**S21 Figure. Variation in the proportion of missing data in populations of *C. bursa-pastoris* and diploid species.** All values have been estimated with sliding windows of 100K genomic positions on the final dataset that was used in the analyses. ASI, EUR and ME are the three populations of *C. bursa-pastoris*. *CO* and *CG* are short forms for *C. orientalis* and *C. grandiflora*, respectively. The regions without the data mostly correspond to pericentromeric regions that were excluded from the data.
