# Supplementary material for: Parental legacy, demography, and admixture influenced the evolution of the two subgenomes of the tetraploid Capsella bursa-pastoris (Brassicaceae)
Source: PLoS Genet. 2019 Feb 15;15(2):e1007949. doi: 10.1371/journal.pgen.1007949 (PMC6395008; doi:10.1371/journal.pgen.1007949)
Supplement: S22 Fig — The original whole genome phylogenetic tree was distorted by gene flow between C. orientalis and C. bursa-pastoris in Asia or different origin of the ASI population. Therefore, we reconstructed an artificial tree that presumably reflects the true history. The clades of C. bursa-pastoris within each subgenome were reconstructed from a neighbor-joining tree of only C. bursa-pastoris. The parental species C. orientalis, C. grandiflora were then placed as basal to each of the corresponding subgenomes. Branch length was discarded from the tree. Red and blue dots indicate the nodes for which the ancestral sequences were reconstructed. (PDF) [file pgen.1007949.s022.pdf]

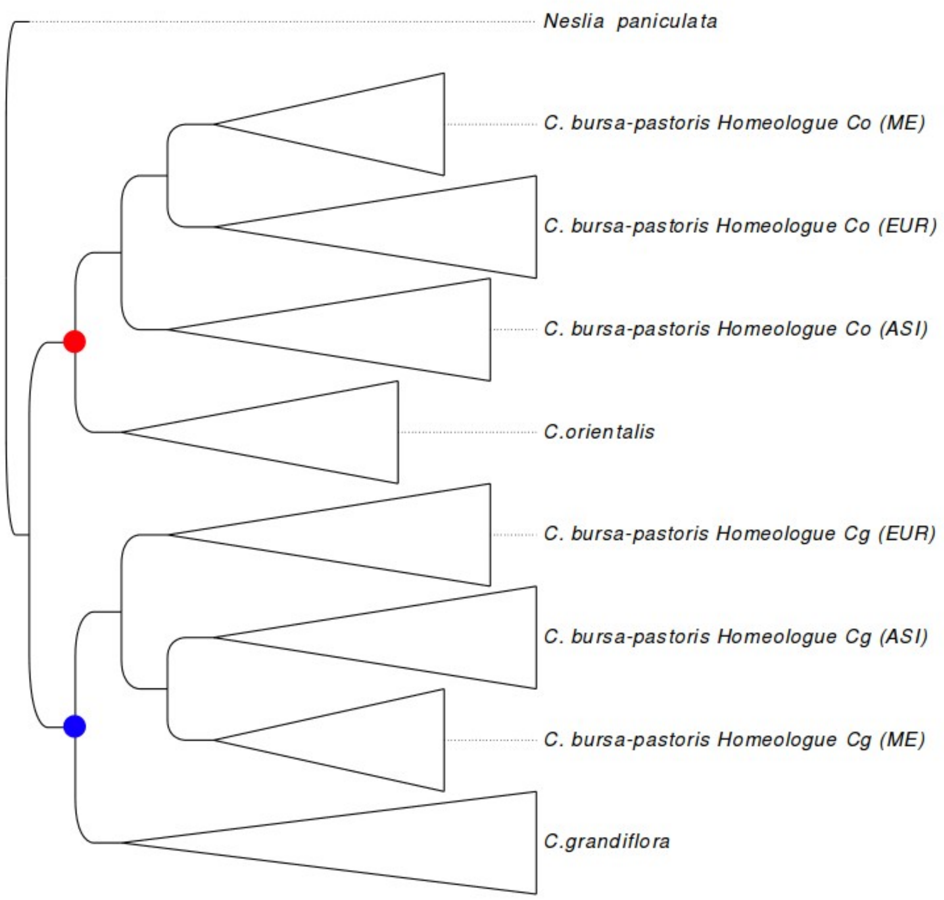

S22 Figure. Tree presumably reflecting the true history of the populations of *C. bursa-pastoris* and parental species. The original whole genome phylogenetic tree was distorted by gene flow between *C. orientalis* and *C. bursa-pastoris* in Asia or different origin of the ASI population. Therefore, we reconstructed an artificial tree that presumably reflects the true history. The clades of *C. bursa-pastoris* within each subgenome were reconstructed from a neighbor-joining tree of only *C. bursa-pastoris*. The parental species *C. orientalis, C. grandiflora* were then placed as basal to each of the corresponding subgenomes. Branch length was discarded from the tree. Red and blue dots indicate the nodes for which the ancestral sequences were reconstructed.
