# Supplementary material for: Parental legacy, demography, and admixture influenced the evolution of the two subgenomes of the tetraploid Capsella bursa-pastoris (Brassicaceae)
Source: PLoS Genet. 2019 Feb 15;15(2):e1007949. doi: 10.1371/journal.pgen.1007949 (PMC6395008; doi:10.1371/journal.pgen.1007949)
Supplement: S23 Fig — The tree was reconstructed for the SNPs data and the divergence axis is scaled to the whole genome values. The bootstrap values of 100 replicates are shown only for the major clades. (PDF) [file pgen.1007949.s023.pdf]

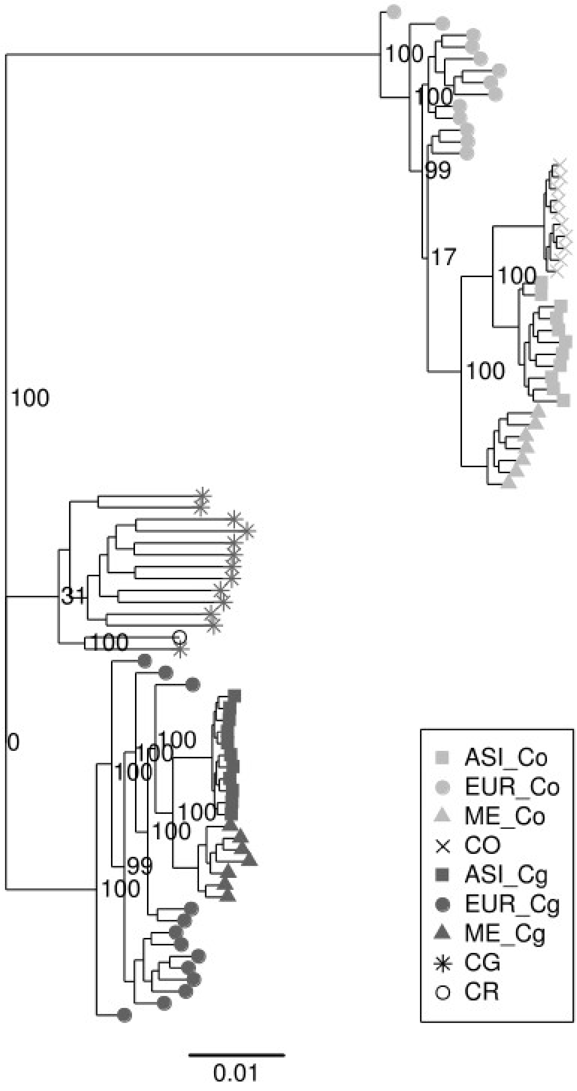

S23 Figure. Maximum likelihood phylogenetic tree of two subgenomes of *C. bursa-pastoris* and its parental species. The tree was reconstructed for the SNPs data and the divergence axis is scaled to the whole genome values. The bootstrap values of 100 replicates are shown only for the major clades.

We observed some difference in the topologies between the ML vs NJ whole genome trees. The NJ tree showed consistent clustering of three populations ASI, EUR and ME that was also identified in the previous studies (Cornille et al. 2016, Kryvokhyzha et al. 2016). However, in the ML tree, the EUR samples were not a monophyletic group in the Cbp_{Co} nor in the Cbp_{Cg} subgenomes. We assumed this discrepancy could be due to the distortion of the whole genome tree by the inter-specific gene. This makes the whole genome tree rather a summary of genetic variation than the tree representing true phylogeny. A more realistic picture is presented by the densitree showing that phylogenetic signal varies in different part of the genome (Fig. 1C).
