# Supplementary material for: Parental legacy, demography, and admixture influenced the evolution of the two subgenomes of the tetraploid Capsella bursa-pastoris (Brassicaceae)
Source: PLoS Genet. 2019 Feb 15;15(2):e1007949. doi: 10.1371/journal.pgen.1007949 (PMC6395008; doi:10.1371/journal.pgen.1007949)
Supplement: S24 Fig — The proportion of deleterious nonsynonymous changes was estimated with SIFT4G on species-specific mutations that are unique alleles for C. orientalis, C. grandiflora and the two subgenomes of C. bursa-pastoris. The left plot shows the results obtained with C. rubella database, the right plot was obtained with A. thaliana database. Co and Cg are the two subgenomes of C. bursa-pastoris. ASI, EUR, ME, CO, CG indicate Asian, European and Middle Eastern populations of C. bursa-pastoris, and parental species C. orientalis and C. grandiflora, respectively. (PDF) [file pgen.1007949.s024.pdf]

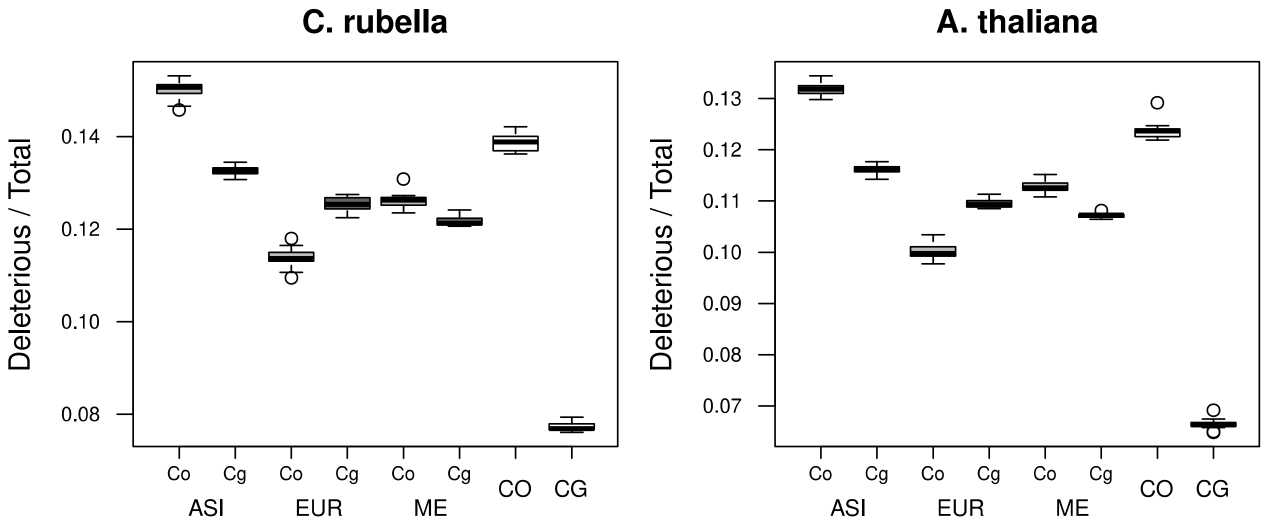

S24 Figure. Genetic load in the subgenomes of *C. bursa-pastoris* and its parental species for species-specific mutations. The proportion of deleterious nonsynonymous changes was estimated with SIFT4G on species-specific mutations that are unique alleles for *C. orientalis, C. grandiflora* and the two subgenomes of *C. bursa-pastoris*. The left plot shows the results obtained with *C. rubella* database, the right plot was obtained with *A. thaliana* database. Co and Cg are the two subgenomes of *C. bursa-pastoris*. ASI, EUR, ME, CO, CG indicate Asian, European and Middle Eastern populations of *C.bursa-pastoris*, and parental species *C. orientalis* and *C. grandiflora*, respectively.
