# Supplementary material for: Parental legacy, demography, and admixture influenced the evolution of the two subgenomes of the tetraploid Capsella bursa-pastoris (Brassicaceae)
Source: PLoS Genet. 2019 Feb 15;15(2):e1007949. doi: 10.1371/journal.pgen.1007949 (PMC6395008; doi:10.1371/journal.pgen.1007949)
Supplement: S1 Table — (PDF) [file pgen.1007949.s025.pdf]

S1 Table. Sequencing and phasing information.

Accession	Total number of reads	Number of mapped reads	Number of mapped paired reads (both in pair)	Mean coverage	SNPs	Heterozygots SNPs	Missing	Phased Heterozygots	SNPs after phasing	Genotypes in whole genome alignment	Genotypes in whole-genome after phasing
12.4	49464054	45557201	44499507	36	6121356	2905233	940549	2346266	4999676	98257035	72118966
13.16	52845124	47568226	46301587	38	6138305	2921106	923600	2298020	4887614	99216717	72118966
16.9	57887747	53690444	52518383	43	6150065	2910658	911840	2351345	5029618	99471512	72224559
1939-12-28	53902068	47795879	46463357	38	6080116	2908808	981789	2330121	4917715	97117816	72224559
5.16	53139204	46608524	45147337	37	5959594	2839505	1102311	2308444	4887015	95675598	72349318
70.5	55124805	49200993	47855451	39	5890303	2811262	1171602	2287111	4833694	95214208	72349318
AL87	59970536	53452762	51908654	39	4993017	2252446	2068888	1847100	4127492	82421369	71516706
BEL5	80937786	71299545	69198355	52	5555348	2690358	1506557	1958817	4099234	91392255	71516706
DL174	76005581	58828043	55756083	43	4874837	2218696	2187068	1838528	4065553	79779739	70582485
FR50	70612809	58168853	56174727	43	5043544	2253542	2018361	1844371	4157102	83989319	70582485
GY37	79610942	70780094	68897241	52	4973428	2254374	2088477	1844561	4098621	82173857	70058290
HJC419	80712836	66977963	64417680	49	5525451	2574615	1536454	2058837	4462904	90246365	70058290
HRB132	75789495	60995844	58043985	45	5886199	2776379	1175706	2217299	4744199	95248326	60514775
HY85	67500437	53986788	51381588	40	5692278	2691156	1369627	2200661	4694684	92006976	60514775
IRRU2	76929364	69564152	67907823	51	5425246	2472742	1636659	1991234	4405418	89432280	66881028
JO56	79470603	67046138	64397023	49	5653123	2649382	1408782	2159880	4651624	91957943	66881028
JZH152	80652085	67163379	64593245	49	5475564	2540707	1586341	2049915	4451070	89469067	59430965
KMB206	78975546	66355143	63957155	49	4970075	2253892	2091830	1848536	4107289	82138896	59430965
NJ219	67776611	57774620	55881123	42	4740856	2144863	2321049	1761379	3919129	78693540	61564298
PL	78544520	70779212	69236572	78	6178036	2997065	883869	2296097	4812102	98704361	61564298
RK32	58340940	52216435	50786351	41	6069313	2886144	992592	2334764	4955856	97453700	61088073
SE14	78611608	67949620	66051904	75	6308942	3038188	752963	2343422	4943223	102149081	61088073
SE33	78120829	68824457	66784671	51	5441078	2494867	1620827	2003876	4403731	89860912	66844290
STA4	76063405	64087118	61595543	47	5167167	2357164	1894738	1916937	4237381	84991358	66844290
STJ2	77687488	67938722	65955342	50	5364007	2442565	1697898	1980790	4389954	89317913	70058648
TBS195	75620897	65288373	63326796	48	4981222	2260746	2080683	1857707	4127273	82446889	70058648
TR73	77624846	64179290	61503301	47	5073550	2309054	1988355	1898751	4207231	83635729	68214092
TY118	68816910	53791171	50983722	39	5072029	2336960	1989876	1940936	4239747	82747183	68214092
VLA3	77085815	65409644	63156669	48	5117585	2306564	1944320	1876328	4197115	85220147	65805269
WAC5	74611864	62596618	59962710	46	5786987	2717785	1274918	2195675	4728586	94034921	65805269
XN444	76675754	63395249	60792822	46	5140099	2356177	1921806	1937268	4254116	84443325	67866392
