# Supplementary material for: Parental legacy, demography, and admixture influenced the evolution of the two subgenomes of the tetraploid Capsella bursa-pastoris (Brassicaceae)
Source: PLoS Genet. 2019 Feb 15;15(2):e1007949. doi: 10.1371/journal.pgen.1007949 (PMC6395008; doi:10.1371/journal.pgen.1007949)
Supplement: S2 Table — (PDF) [file pgen.1007949.s026.pdf]

S2 Table. Nucleotide diversity *π* and absolute divergence *Dxy* between different subgenomes of *C. bursa-pastoris* populations and its parental species.

Species		*C. b-p.*			CO
		ASI_Co	EUR_Co	ME_Co	
Cbp	ASI_Co	0.0030			
	EUR_Co	0.0083	0.0046		
	ME_Co	0.0076	0.0072	0.0032	
CO		0.0071	0.0097	0.0089	0.0020

Species		*C. b-p.*			*CG*
		ASI_Cg	EUR_Cg	ME_Cg	
Cbp	ASI_Cg	0.0017			
	EUR_Cg	0.0072	0.0051		
	ME_Cg	0.0064	0.0073	0.0034	
CG		0.0213	0.0203	0.0209	0.0174

The diagonal values (in gray) correspond to π of the corresponding subgenome within each population/species. The below diagonal values represent Dxy values. ASI, EUR and ME are the three differentiated populations of *C. bursa-pastoris* (*Cbp*) with Co and Cg indicating corresponding subgenomes. *CO* and *CG* are short forms for *C. orientalis* and *C. grandiflora*, respectively.
