# Supplementary material for: Parental legacy, demography, and admixture influenced the evolution of the two subgenomes of the tetraploid Capsella bursa-pastoris (Brassicaceae)
Source: PLoS Genet. 2019 Feb 15;15(2):e1007949. doi: 10.1371/journal.pgen.1007949 (PMC6395008; doi:10.1371/journal.pgen.1007949)
Supplement: S3 Table — (PDF) [file pgen.1007949.s027.pdf]

S3 Table. Multiple comparisons for the generalized linear model of the topology weighting of the *Cbp*$_{Co}$ subgenome of *C. bursa-pastoris* and *C. orientalis*.

Comparison	Estimate	Std. Error	z value	p-value
ASI_CO - species	0.728	0.017	43.50	0.0000
ASI_CO - EUR_CO	0.320	0.017	18.39	0.0000
ASI_CO - ME_CO	0.333	0.017	19.16	0.0000
EUR_CO - species	0.408	0.016	25.68	0.0000
EUR_CO - ME_CO	0.013	0.017	0.77	0.8678
ME_CO - species	0.395	0.016	24.92	0.0000

With the binomial family, the null deviance was 214077 on 198584 degrees of freedom, and residual deviance of 210324 on 198580 degrees of freedom.
