# Supplementary material for: Parental legacy, demography, and admixture influenced the evolution of the two subgenomes of the tetraploid Capsella bursa-pastoris (Brassicaceae)
Source: PLoS Genet. 2019 Feb 15;15(2):e1007949. doi: 10.1371/journal.pgen.1007949 (PMC6395008; doi:10.1371/journal.pgen.1007949)
Supplement: S4 Table — (PDF) [file pgen.1007949.s028.pdf]

S4 Table. Multiple comparisons for the generalized linear model of the topology weighting of the *Cbp*$_{Cg}$ subgenome of *C. bursa-pastoris* and *C. rubella*.

Comparison	Estimate	Std. Error	z value	p-value
ASI_CR - species	-3.711	0.022	-166.33	0.0000
ASI_CR - EUR_CR	-1.425	0.016	-90.97	0.0000
ASI_CR - ME_CR	-0.797	0.016	-50.48	0.0000
EUR_CR - species	-2.286	0.021	-107.07	0.0000
EUR_CR - ME_CR	0.628	0.014	43.65	0.0000
ME_CR - species	-2.914	0.021	-135.94	0.0000

With the binomial family, the null deviance was 274261 on 198584 degrees of freedom, and residual deviance of 225829 on 198580 degrees of freedom.
