# Supplementary material for: Parental legacy, demography, and admixture influenced the evolution of the two subgenomes of the tetraploid Capsella bursa-pastoris (Brassicaceae)
Source: PLoS Genet. 2019 Feb 15;15(2):e1007949. doi: 10.1371/journal.pgen.1007949 (PMC6395008; doi:10.1371/journal.pgen.1007949)
Supplement: S5 Table — (PDF) [file pgen.1007949.s029.pdf]

S5 Table. Multiple comparisons for the generalized linear model of the topology weighting of the *Cbp*$_{Cg}$ subgenome and *C. grandiflora*.

Comparison	Estimate	Std. Error	z value	p-value
ASI_CG - species	-3.783	0.028	-133.48	0.0000
ASI_CG - EUR_CG	-0.881	0.015	-60.55	0.0000
ASI_CG - ME_CG	-0.402	0.014	-27.77	0.0000
EUR_CG - species	-2.902	0.028	-102.68	0.0000
EUR_CG - ME_CG	0.480	0.014	33.50	0.0000
ME_CG - species	-3.381	0.028	-119.83	0.0000

With the binomial family, the null deviance was 262921 on 198584 degrees of freedom, and residual deviance of 219219 on 198580 degrees of freedom.
