# Supplementary material for: Parental legacy, demography, and admixture influenced the evolution of the two subgenomes of the tetraploid Capsella bursa-pastoris (Brassicaceae)
Source: PLoS Genet. 2019 Feb 15;15(2):e1007949. doi: 10.1371/journal.pgen.1007949 (PMC6395008; doi:10.1371/journal.pgen.1007949)
Supplement: S6 Table — (PDF) [file pgen.1007949.s030.pdf]

S6 Table. Results of the ABBA-BABA tests assessing the admixture between *C. bursa-pastoris* and *C. orientalis*, *C. rubella* for the unphased data.

P1	***P2***	***P3***	***D***	***D error***	***Z-score***	***P-value***	***f***	***f error***
EUR	ASI	CO	0.08	0.01	9.40	<0.0001	6.6%	0.7%
ME	ASI	CO	0.05	0.01	6.18	<0.0001	4.1%	0.7%
ME	EUR	CO	-0.03	0.01	-5.72	<0.0001	-2.7%	0.5%
EUR	ASI	CR	-0.13	0.01	-11.50	<0.0001	-7.0%	0.7%
ME	ASI	CR	-0.07	0.01	-6.32	<0.0001	-3.6%	0.6%
ME	EUR	CR	0.06	0.01	5.05	<0.0001	3.2%	0.6%

P1, P2, and P3 refer to the three populations used in the ABBA-BABA tests. A significantly positive *D* indicates admixture between *P2* and *P3*. *f* provides an estimate of the fraction of admixture. *Z*-score and *P*-value were estimated with the block jack-knife method. The error term corresponds to a standard error. ASI, EUR and ME are the three populations of *C. bursa-pastoris*. CO and CR stand for *C. orientalis* and *C. rubella*, respectively. The unphased data set comprised the same genomic positions as the phased data (12.8Mb in total length).
