# Supplementary material for: Parental legacy, demography, and admixture influenced the evolution of the two subgenomes of the tetraploid Capsella bursa-pastoris (Brassicaceae)
Source: PLoS Genet. 2019 Feb 15;15(2):e1007949. doi: 10.1371/journal.pgen.1007949 (PMC6395008; doi:10.1371/journal.pgen.1007949)
Supplement: S7 Table — (PDF) [file pgen.1007949.s031.pdf]

S7 Table. Results of the ABBA-BABA tests assessing the admixture between *C. bursa-pastoris* and *C. orientalis*, *C. rubella* for the complete phased data.

P1	***P2***	***P3***	***D***	***D* error**	***Z*-score**	***P*-value**	***f***	***f* error**
EUR	ASI	CO	0.47	0.05	8.68	<0.0001	19.0%	2.5%
ME	ASI	CO	0.38	0.06	5.91	<0.0001	13.8%	2.6%
ME	EUR	CO	-0.21	0.06	-3.57	0.0004	-6.4%	2.0%
EUR	ASI	CR	-0.73	0.03	-26.40	<0.0001	-16.1%	2.1%
ME	ASI	CR	-0.60	0.04	-14.14	<0.0001	-8.3%	1.5%
ME	EUR	CR	0.31	0.07	4.30	<0.0001	6.8%	1.6%

P1, P2, and P3 refer to the three populations used in the ABBA-BABA tests. A significantly positive *D* indicates admixture between *P2* and *P3*. *f* provides an estimate of the fraction of admixture. *Z*-score and *P*-value were estimated with the block jack-knife method. The error term corresponds to a standard error. ASI, EUR and ME are the three populations of *C. bursa-pastoris*. CO and CR stand for *C. orientalis* and *C. rubella*, respectively. The complete dataset (i.e. data without any missing point) comprised 1,427,289 sites.
