# Supplementary material for: Parental legacy, demography, and admixture influenced the evolution of the two subgenomes of the tetraploid Capsella bursa-pastoris (Brassicaceae)
Source: PLoS Genet. 2019 Feb 15;15(2):e1007949. doi: 10.1371/journal.pgen.1007949 (PMC6395008; doi:10.1371/journal.pgen.1007949)
Supplement: S8 Table — (PDF) [file pgen.1007949.s032.pdf]

S8 Table. Summary of the probabilities and proportions of admixture from HAPMIX analyses of each of the *C. bursa-pastoris* subgenomes.

Focal Population	Diploid Reference Population	Alt. Reference Population	Mean Genome-wide Admixture Probability	Mean Introg. Probability | > .5	Mean Introg. Probability | < .5	Proportion of Admixture Genome-wide	n	n (Admixed Sites)
Cbp_{Cg} subgenome								
EUR_Cg	CR	ASI_Cg	0.242	0.682	0.144	0.182	8798	1597
EUR_Cg	CG	ASI_Cg	0.115	0.650	0.075	0.069	19122	1327
ME_Cg	CR	ASI_Cg	0.128	0.668	0.060	0.111	6776	754
ME_Cg	CG	ASI_Cg	0.084	0.684	0.043	0.063	14422	910
ASI_Cg	CR	EUR_Cg	0.022	0.822	0.005	0.020	52959	1075
ASI_Cg	CG	EUR_Cg	0.003	0.809	0.001	0.002	76408	186
Cbp_{Co} subgenome								
EUR_Co	CR	ASI_Co	0.013	0.000	0.013	0.000	11441	0
EUR_Co	CO	ASI_Co	0.456	0.635	0.326	0.421	76758	32310
EUR_Co	CO	ME_Co	0.288	0.612	0.234	0.144	50472	7252
ME_Co	CR	ASI_Co	0.006	0.000	0.006	0.000	9656	0
ME_Co	CO	ASI_Co	0.214	0.786	0.113	0.188	14903	2803
ME_Co	CO	EUR_Co	0.189	0.652	0.148	0.082	68516	5628
ASI_Co	CR	EUR_Co	0.004	0.000	0.004	0.000	17560	0
ASI_Co	CO	EUR_Co	0.442	0.766	0.245	0.379	88001	33330
ASI_Co	CO	ME_Co	0.289	0.707	0.216	0.148	44730	6638

For each analysis, the reported probabilities and proportion of admixture refers to admixture between the diploid reference population and the focal population. Estimates exclude sites that fall within centromeric regions.
