# Supplementary material for: Parental legacy, demography, and admixture influenced the evolution of the two subgenomes of the tetraploid Capsella bursa-pastoris (Brassicaceae)
Source: PLoS Genet. 2019 Feb 15;15(2):e1007949. doi: 10.1371/journal.pgen.1007949 (PMC6395008; doi:10.1371/journal.pgen.1007949)
Supplement: S9 Table — (PDF) [file pgen.1007949.s033.pdf]

S9 Table. Multiple comparisons for the generalized linear model on the genetic load estimated with the *C. rubella* SIFT4G database.

Comparison	Estimate	Std. Error	z value	p-value
ASI_Co - ASI_Cg	0.2243	0.0190	11.82	0.0000
EUR_Co - EUR_Cg	0.0995	0.0174	5.72	0.0000
ME_Co - ME_Cg	0.1997	0.0232	8.61	0.0000
CO - CG	0.8747	0.0147	59.44	0.0000
CG - ASI_Cg	-0.7008	0.0134	-52.43	0.0000
EUR_Cg - CG	0.4557	0.0135	33.73	0.0000
ME_Cg - CG	0.5337	0.0163	32.83	0.0000
CO - ASI_Co	-0.0504	0.0199	-2.52	0.1317
EUR_Co - CO	-0.3195	0.0184	-17.40	0.0000
ME_Co - CO	-0.1412	0.0222	-6.38	0.0000
EUR_Cg - ASI_Cg	-0.2451	0.0149	-16.42	0.0000
ME_Cg - ASI_Cg	-0.1670	0.0175	-9.57	0.0000
ME_Cg - EUR_Cg	0.0781	0.0176	4.44	0.0002
EUR_Co - ASI_Co	-0.3699	0.0210	-17.62	0.0000
ME_Co - ASI_Co	-0.1916	0.0244	-7.86	0.0000
ME_Co - EUR_Co	0.1783	0.0231	7.72	0.0000

Dispersion parameter for the quasibinomial family was 7.88, with null deviance of 48724 on 84 degrees of freedom, and residual deviance of 612 on 77 degrees of freedom.
