# Supplementary material for: Parental legacy, demography, and admixture influenced the evolution of the two subgenomes of the tetraploid Capsella bursa-pastoris (Brassicaceae)
Source: PLoS Genet. 2019 Feb 15;15(2):e1007949. doi: 10.1371/journal.pgen.1007949 (PMC6395008; doi:10.1371/journal.pgen.1007949)
Supplement: S10 Table — (PDF) [file pgen.1007949.s034.pdf]

S10 Table. Multiple comparisons for the generalized linear model on the genetic load estimated with the *A. thaliana* SIFT4G database.

Comparison	Estimate	Std. Error	z value	p-value
ASI_Co - ASI_Cg	0.1446	0.0066	22.00	0.0000
EUR_Co - EUR_Cg	-0.1000	0.0061	-16.51	0.0000
ME_Co - ME_Cg	0.0576	0.0079	7.31	0.0000
CO - CG	0.6857	0.0057	119.40	0.0000
CG - ASI_Cg	-0.6140	0.0046	-132.33	0.0000
EUR_Cg - CG	0.5485	0.0048	114.21	0.0000
ME_Cg - CG	0.5236	0.0058	91.02	0.0000
CO - ASI_Co	-0.0730	0.0074	-9.87	0.0000
EUR_Co - CO	-0.2372	0.0068	-34.74	0.0000
ME_Co - CO	-0.1045	0.0079	-13.26	0.0000
EUR_Cg - ASI_Cg	-0.0655	0.0054	-12.04	0.0000
ME_Cg - ASI_Cg	-0.0905	0.0063	-14.36	0.0000
ME_Cg - EUR_Cg	-0.0249	0.0064	-3.88	0.0016
EUR_Co - ASI_Co	-0.3102	0.0071	-43.75	0.0000
ME_Co - ASI_Co	-0.1775	0.0081	-21.89	0.0000
ME_Co - EUR_Co	0.1328	0.0076	17.48	0.0000

Dispersion parameter for the quasibinomial family was 0.81, with null deviance of 29943 on 85 degrees of freedom, and residual deviance of 62 on 77 degrees of freedom.
