# Supplementary material for: Parental legacy, demography, and admixture influenced the evolution of the two subgenomes of the tetraploid Capsella bursa-pastoris (Brassicaceae)
Source: PLoS Genet. 2019 Feb 15;15(2):e1007949. doi: 10.1371/journal.pgen.1007949 (PMC6395008; doi:10.1371/journal.pgen.1007949)
Supplement: S11 Table — (PDF) [file pgen.1007949.s035.pdf]

S11 Table. Multiple comparisons for the generalized linear model testing for the expression difference between populations.

Comparison	Estimate	Std. Error	z value	p-value
ASI – EUR	0.0197	0.0020	9.92	0.0000
ASI – ME	0.0229	0.0025	9.02	0.0000
EUR – ME	0.0032	0.0026	1.25	0.0100

Dispersion parameter for the quasibinomial family was 0.069, with null deviance of 25444 on 344116 degrees of freedom, and residual deviance of 25435 on 344114 degrees of freedom.
