# Supplementary material for: Parental legacy, demography, and admixture influenced the evolution of the two subgenomes of the tetraploid Capsella bursa-pastoris (Brassicaceae)
Source: PLoS Genet. 2019 Feb 15;15(2):e1007949. doi: 10.1371/journal.pgen.1007949 (PMC6395008; doi:10.1371/journal.pgen.1007949)
Supplement: S12 Table — (PDF) [file pgen.1007949.s036.pdf]

S12 Table. Alternative combinations used in the ABBA-BABA tests.

P_1	P_2	P_3	*D*±error	*Z*-score	*P*-value	*f*±error (%)
EUR_Cg	ASI_Cg	CO	0.20±0.02	9.94	<0.0001	2.2±0.3
ME_Cg	ASI_Cg	CO	0.20±0.02	8.07	<0.0001	1.8±0.3
EUR_Cg	ME_Cg	CO	0.04±0.01	4.40	<0.0001	0.4±0.1
ASI_Co	EUR_Co	CG	0.02±0.01	1.27	0.2042	0.4±0.3
ME_Co	ASI_Co	CG	0.02±0.01	1.40	0.1619	0.4±0.3
ME_Co	EUR_Co	CG	0.04±0.01	3.71	<0.0002	0.8±0.2
ASI_Co	EUR_Co	CR	0.06±0.02	3.57	<0.0004	0.8±0.2
ASI_Co	ME_Co	CR	0.00±0.02	0.28	0.7817	0.1±0.2
ME_Co	EUR_Co	CR	0.06±0.01	4.8	<0.0001	0.7±0.2
CR	CG	CO	0.21±0.01	33.64	<0.0001	3.1±0.1

P_1, P_2, and P_3 refer to the three populations used in the ABBA-BABA tests. A significantly positive *D* indicates admixture between P_2 and P_3. *f* provides an estimate of the fraction of admixture. *Z*-score and *P*-value were estimated with the block jack-knife method. The error term corresponds to a standard error. ASI, EUR and ME are the three populations of *C. bursa-pastoris* with _Co and _Cg indicating different subgenomes. CO, CG, and CR are short forms for *C. orientalis, C. grandiflora, C. rubella*, respectively.
