# Supplementary material for: Parental legacy, demography, and admixture influenced the evolution of the two subgenomes of the tetraploid Capsella bursa-pastoris (Brassicaceae)
Source: PLoS Genet. 2019 Feb 15;15(2):e1007949. doi: 10.1371/journal.pgen.1007949 (PMC6395008; doi:10.1371/journal.pgen.1007949)
Supplement: S6 Fig — The trees were reconstructed with the neighbor-joining algorithm and absolute genetic distance for non-overlapping 1 K windows with minimum 100 complete sites (784 trees). HomeoRoq phased samples have _A and _B in their names to indicate CbpCg and CbpCo subgenomes, respectively, whereas samples phased with HapCUT are marked with _Cg and _Co for the corresponding subgenomes. ASI, EUR ME indicate Asian, European and Middle Eastern populations of C. bursa-pastoris, respectively. (PDF) [file pgen.1007949.s006.pdf]

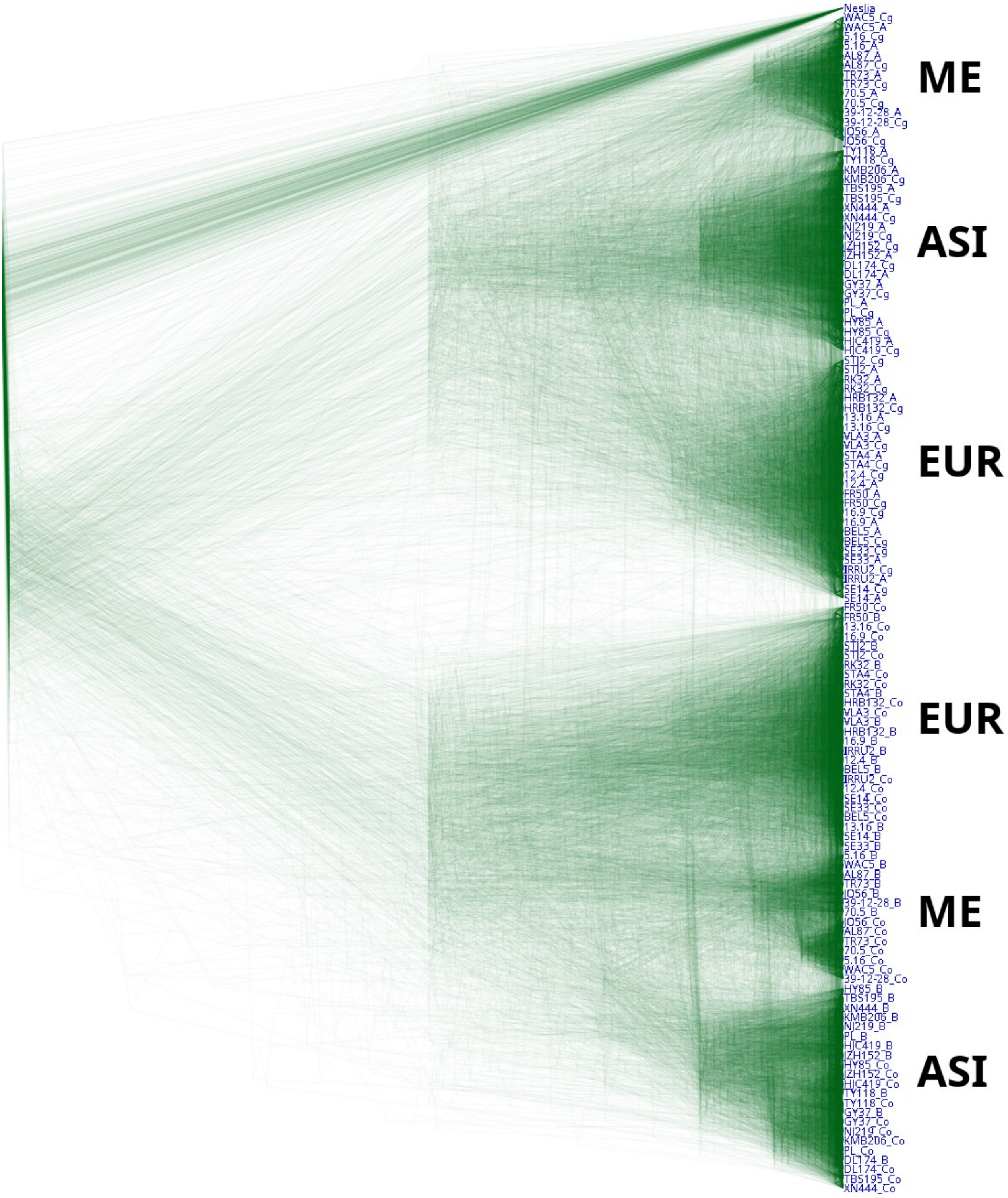

S6 Figure. Phylogenetic relationship reconstructed for 1K windows for the alternatively phased datasets. The trees were reconstructed with the neighbor-joining algorithm and absolute genetic distance for non-overlapping 1K windows with minimum 100 complete sites (784 trees). HomeoRoq phased samples have _A and _B in their names to indicate *Cbp*$_{Cg}$ and *Cbp*$_{Co}$ subgenomes, respectively, whereas samples phased with HapCUT are marked with _Cg and _Co for the corresponding subgenomes. ASI, EUR ME indicate Asian, European and Middle Eastern populations of *C. bursa-pastoris*, respectively.
