# Supplementary material for: Parental legacy, demography, and admixture influenced the evolution of the two subgenomes of the tetraploid Capsella bursa-pastoris (Brassicaceae)
Source: PLoS Genet. 2019 Feb 15;15(2):e1007949. doi: 10.1371/journal.pgen.1007949 (PMC6395008; doi:10.1371/journal.pgen.1007949)
Supplement: S1 Appendix — (PDF) [file pgen.1007949.s037.pdf]

S1 Appendix. Artificial crosses

Plant material and growth condition

All seeds were surface-sterilised using 10% (vol/vol) chlorine. After sterilization, seeds were plated on Petri dishes with Murashige and Skoog medium (MS). For stratification, seeds were put in darkness for 7 days at 4°C. After the stratification, agar plates were moved to a growth chamber at 22 °C with 16h light and 8h darkness, under the light intensity of 130 lmol/m2/s for germination. After 7 days, germinated seedlings were transferred to soil and grown in the growth chamber under the same condition used for the germination.

Plant crosses

For all crosses (Table A), designated mother plants (individuals of *C. orientalis, C. rubella* and *C. grandiflora*) were emasculated, and 3 days after the emasculation the pistils were pollinated by hand, using pollen from individuals of ASI, EUR, ME populations of *C. bursa-pastoris*. *C. grandiflora* was used as pollen donor only for *C. rubella* plants. Reciprocal crosses were made. For each cross, at least three biological replicates were made, with the exception of crosses between *C. grandiflora* and ME for which only two biological replicates were made. Each biological replicates consisted of four or more siliques.

For each mother plant used, five buds were collected in order to estimate the number of ovules per silique. To count the number of ovules per each gynoecium, buds were manually dissected and observed under a stereomicroscope.

In order to verify the presence of prezygotic barriers that prevent hybridization, we estimated the fertilization rate (number of seeds produced / number of ovules). The abortion rate (number of aborted seeds / number of seeds produced) was estimated to verify the presence of post-zygotic barriers. Lastly, the seed set (number of normally shaped seeds / number of seeds produced) was estimated.

Results

Crosses between *C. rubella* (CR) and ASI, EUR and ME were successful, in both directions, leading to normally shaped seeds (Table B). The low percentage of aborted seeds (0.22-1.18%) suggests the absence of post-zygotic barriers to hybridization at this stage of development. One biological replicate of the cross CR x ASI (female x male) failed and the siliques did not set any seed (likely due to an operator mistake).

Reciprocal crosses between *C. rubella,* and *C. grandiflora* led to mostly aborted seeds (87-98%, Table B) suggesting the presence of postzygotic barriers to prevent gene flow.

Reciprocal crosses between *C. orientalis* and ASI, EUR and ME also resulted in seed abortion. When *C. orientalis* was used as mother plant, the ovule fertilization took place, but led to mainly aborted seeds (92-100%, Table C). The low percentage of normally shaped seeds produced in the crosses CO x ASI, CO x EUR and CO x ME was probably due to cross-contamination. When *C. orientalis* was used as pollen donor, the seeds aborted in an early stage of development, resulting in very tiny aborted seeds. Our results suggest the presence of post-zygotic barriers to hybridization between *C. orientalis* and *C. bursa-pastoris.*

Reciprocal crosses between *C. grandiflora* and ASI, EUR and ASI mostly led to aborted seeds (93-100%, Table D), suggesting the existence of post-zygotic barriers to gene flow. A few normally shaped seeds produced are probably due to cross-contamination. For the cross Cg x ME only two biological replicates were made and further investigations are necessary.

Table A. Number and description of biological replicates per cross.

C. rubella			
CR♀ x ASI ♂	3 biological replicates:<br>2 well-formed<br>1 early aborted seeds	ASI ♀ x CR ♂	3 biological replicates:<br>all well-formed
CR♀ x EUR ♂	4 biological replicates:<br>all well-formed	EUR ♀ x CR ♂	4 biological replicates:<br>all well-formed
CR♀ x ME ♂	5 biological replicates:<br>all well-formed	ME ♀ x CR ♂	5 biological replicates:<br>all well-formed
CR♀ x CG ♂	4 biological replicates:<br>all brownish aborted seeds	Cg ♀ x CR ♂	6 biological replicates:<br>- 5 dark and shrivelled<br>- 1 early aborted seeds
C. orientalis			
CO♀ x ASI ♂	2 biological replicates:<br>- 2 dark and shrivelled	ASI ♀ x CO ♂	2 biological replicates:<br>- 2 early aborted seeds
CO♀ x EUR ♂	3 biological replicates:<br>- 1 early aborted seeds<br>- 2 dark and shrivelled	EUR ♀ x CO ♂	3 biological replicates:<br>- 3 early aborted seeds
CO♀ x ME ♂	4 biological replicates:<br>- 4 dark and shrivelled	ME ♀ x CO ♂	4 biological replicates:<br>- 4 early aborted seeds
C. grandiflora			
CG♀ x ASI ♂	3 biological replicates:<br>all crosses failed	ASI ♀ x CG ♂	3 biological replicates:<br>all dark and shrivelled
CG♀ x EUR ♂	4 biological replicates:<br>- 1 failed<br>- 2 early aborted seeds	EUR ♀ x CG ♂	4 biological replicates:<br>all dark and shrivelled
CG♀ x ME ♂	2 biological replicates:<br>- 1 failed<br>- 1 early aborted seeds	ME ♀ x CG ♂	2 biological replicates:<br>all dark and shrivelled

Table B. Results of the crosses between *C.rubella*, *C.bursa-pastoris* and *C.grandiflora*.

Cross	Aborted seeds	Seeds
CR♀ x Cbp ASI ♂	34.40(32.81)	65.60 (32.81)
Cbp ASI ♀ x CR ♂	0.71 (0.45)	99.29 (0.45)
CR♀ x Cbp EUR ♂	0.22 (0.022)	99.77 (0.22)
Cbp EUR ♀ x CR ♂	1.18 (0.78)	98.82 (0.78)
CR♀ x Cbp ME ♂	0.72 (0.55)	99.28 (0.55)
Cbp ME ♀ x CR ♂	0.75 (0.41)	99.25 (0.42)
CR♀ x CG ♂	98.87 (0.82)	1.13 (0.82)
CG ♀ x CR ♂	87.47 (9.95)	12.51 (9.95)

Cbp, CR and CG are short forms for *C. bursa-pastoris, C. rubella, and C. grandiflora*, respectively. The directionality of the cross is indicated as mother plant (♀) x pollen donor (♂). Percentage of aborted seeds and normally shaped seeds are reported. Standard error is reported in brackets.

Table C. Results of the crosses between *C. orientalis* and *C. bursa-pastoris*.

Cross	Aborted seeds	Seeds
CO ♀ x Cbp ASI ♂	92.45 (6.51)	7.55 (6.51)
Cbp ASI ♀ x CO ♂	100	0
CO ♀ x Cbp EUR ♂	92.53 (7.47)	7.47 (7.47)
Cbp EUR ♀ x CO ♂	100	0
CO ♀ x Cbp ME ♂	99.01 (0.76)	0.98 (0.76)
Cbp ME ♀ x CO ♂	100	0

Cbp and CO are short forms for *C. bursa-pastoris,* and *C. orientalis*, respectively. The directionality of the cross is indicated as mother plant (♀) x pollen donor (♂). Percentage of aborted seeds and normally shaped seeds are reported. Standard error is reported in brackets.

Table D. Results of the crosses between *C. grandiflora* and *C. bursa-pastoris*.

Cross	Aborted seeds	Seeds
CG♀ x Cbp ASI ♂	100	0
Cbp ASI ♀ x CG ♂	100	0
CG♀ x Cbp EUR ♂	93.17 (3.32)	6.82 (3.32)
Cbp EUR ♀ x CG ♂	100	0
CG♀ x Cbp ME ♂	100	0
Cbp ME ♀ x CG ♂	98.78 (1.22)	1.22 (1.22)

Cbp and CG are short forms for *C. bursa-pastoris,* and *C. grandiflora*, respectively. The directionality of the cross is indicated as mother plant (♀) x pollen donor (♂). Percentage of aborted seeds and normally shaped seeds are reported. Standard error is reported in brackets
